# Helveticoside is a biologically active component of the seed extract of *Descurainia sophia* and induces reciprocal gene regulation in A549 human lung cancer cells

**DOI:** 10.1186/s12864-015-1918-1

**Published:** 2015-09-18

**Authors:** Bu-Yeo Kim, Jun Lee, No Soo Kim

**Affiliations:** KM-Convergence Research Division, Korea Institute of Oriental Medicine, 1672 Yuseong-daero, Yuseong-gu Daejeon, 305-811 Republic of Korea; Department of Korean Medicine Life Science and Technology, Korea University of Science and Technology, Daejeon, Republic of Korea

**Keywords:** Connectivity map, *Descurainia sophia*, Helveticoside, Microarray, Reciprocal regulation

## Abstract

**Background:**

Although the pharmacological activities of the seed extract of *Descurainia sophia* have been proven to be useful against cough, asthma, and edema, the biologically active components, particularly at the molecular level, remain elusive. Therefore, we aimed to identify the active component of an ethanol extract of *D. sophia* seeds (EEDS) by applying a systematic genomic approach.

**Results:**

After treatment with EEDS, the dose-dependently expressed genes in A549 cells were used to query the Connectivity map to determine which small molecules could closely mimic EEDS in terms of whole gene expression. Gene ontology and pathway analyses were also performed to identify the functional involvement of the drug responsive genes. In addition, interaction network and enrichment map assays were implemented to measure the functional network structure of the drug-responsive genes. A Connectivity map analysis of differentially expressed genes resulted in the discovery of helveticoside as a candidate drug that induces a similar gene expression pattern to EEDS. We identified the presence of helveticoside in EEDS and determined that helveticoside was responsible for the dose-dependent gene expression induced by EEDS. Gene ontology and pathway analyses revealed that the metabolism and signaling processes in A549 cells were reciprocally regulated by helveticoside and inter-connected as functional modules. Additionally, in an ontological network analysis, diverse cancer type-related genes were found to be associated with the biological functions regulated by helveticoside.

**Conclusions:**

Using bioinformatic analyses, we confirmed that helveticoside is a biologically active component of EEDS that induces reciprocal regulation of metabolism and signaling processes. Our approach may provide novel insights to the herbal research field for identifying biologically active components from extracts.

**Electronic supplementary material:**

The online version of this article (doi:10.1186/s12864-015-1918-1) contains supplementary material, which is available to authorized users.

## Background

*Descurainia sophia* (L.) Webb ex Prantl., also known as Flixweed, belongs to the family Brassicaceae, which is also called Cruciferae. Traditionally, the seeds of *D. sophia* have been used to treat various ailments, including cough, asthma, and edema. We previously isolated diverse compounds showing cytotoxic and anti-inflammatory activities, including glycosides, from the seeds of *D. sophia* [1]. Our previous results are consistent with other reports that *D. sophia* possesses biologically active secondary metabolites, such as cardiac glycosides [2], sulfur glycosides [3], nor-lignan [4], and lactones [5]. We demonstrated that treatment with EEDS up- or down-regulates diverse genes that are closely associated with numerous genome-wide biological functions [6]. However, despite the therapeutic constituents that have been identified thus far in EEDS, the pharmacological effects of EEDS have not been well-characterized, particularly on the molecular level, largely due to the chemical complexity of EEDS.

The difficulty of elucidating molecular mechanisms is a common problem in herbal extract research and significantly influences the course of novel drug development from herbal extracts. The application of genomic and bioinformatic approaches could greatly reduce the time and effort required to identify the mechanisms of pharmacologically active candidate molecules. Therefore, in the present study, we used the Connectivity map, a comprehensive database for chemical genomic information, to identify the biologically active components of EEDS and elucidate its putative pharmacological activity. Genomic expression profiles and network analyses were also applied to identify global regulatory mechanisms.

## Methods

### Preparation of EEDS

Dried seeds of *D. sophia* were commercially obtained from the Kwangmyungdang Medicinal Herbs Co. (Ulsan, Republic of Korea) and identified by Dr. Go Ya Choi at the Korea Institute of Oriental Medicine (KIOM), Daejeon, Republic of Korea. A voucher specimen (KIOM-CRC-5) was deposited at the Cancer Research Center, Herbal Medicine Research Division, KIOM. EEDS was prepared as described in our previous report [6]. EEDS was dissolved in 100 % dimethyl sulfoxide (DMSO, Sigma, St Louis, MO, U.S.A.) at a concentration of 20 mg/mL and stored at −80 °C for further studies.

### Purification of helveticoside from EEDS

Helveticoside, isoquercitrin, quercetin 3-*O-α*-_L_-rhamnopyranosyl-(1 → 2)-*α*-_L_-arabinopyranose, isorhamnetin-3-*O-β*-_D_-glucopyranoside, and drabanemoroside were isolated from EEDS using a chromatographic method and identified by NMR studies as described in our previous study [1].

### Ultra high performance liquid chromatography (UHPLC) analysis

UHPLC analysis was performed using an Agilent UHPLC system (1290 Infinity, Waldbronn, Germany) consisting of a binary pump VL (G4220B), a diode array detector (G4212A, DAD), a sampler (G4226A), a thermostatted column compartment (G1316A), and a thermostat (G1330B). The system was operated by OpenLAB CDS (ChemStation Edition) software (Agilent Technologies, Santa Clara, CA, USA). HPLC grade acetonitrile, methanol, acetic acid (J.T. Baker, Center Valley, PA, USA), and ultrapure water (Millipore RiOs & Milli-Q-Gradient water purification system, Millipore, Bedford, MA, USA) were used for the analyses. A Kinetex C18 column (50 × 2.1 mm, id, 1.7 *μ*m, Agilent) with a mobile phase consisting of acetonitrile and 0.1 % acetic acid in water was used. The mobile phase gradient elution was programmed as follows: acetonitrile 1–5 % (0–7 min), 5–20 % (7–27 min), and 20–60 % (27–40 min). The flow rate of the mobile phase was set to 0.3 mL/min. The sample injection volume was set to 2.0 *μ*L. The column temperature was maintained at 40 °C, and the UV detector was set to 254 and 220 nm. The sample solutions for the UHPLC analyses, including the EEDS (2,000 μg/mL, 80 % methanol), ethyl acetate (EtOAc) fraction (2,000 μg/mL, 100 % methanol), and helveticoside (100 μg/mL 100 % methanol) were filtered (Millex-FG 0.2  μ m, Millipore) prior to the injections.

### Cell culture

A549 human lung cancer cells were directly obtained from the American Type Culture Collection (ATCC, CCL-185, Manassas, VA, USA). Authentication of the cell line was done using a short tandem repeat analysis by Korean Cell Line Bank (Seoul National University College of Medicine, Seoul, Republic of Korea). The cells were grown in RPMI1640 supplemented with 10 % (v/v) fetal bovine serum, 100 U/mL penicillin, and 100 μg/mL streptomycin in 5 % CO_2_ humidified air at 37 °C. All the supplements and basal media used for the cell cultures were purchased from Invitrogen (Carlsbad, CA, USA).

### Microarray experiment

One day before drug treatment A549 cells were seeded and cultured on 100 mm dishes. Next, cells were exposed to increasing concentrations of EEDS (0–20 μg/mL) or helveticoside (0–60 nM) for 24 h. Total RNA was prepared from A549 cells using the Easy-SpinTM total RNA extraction kit (iNtRON Biotechnology, Seoul, Republic of Korea) following the manufacturer’s instructions. The RNA quality was determined using an Agilent 2100 Bioanalyzer (Agilent Technologies, Santa Clara, CA, USA). Only those samples with an RNA integrity number (RIN) greater than 7.0 were included in the microarray analysis. The equal amounts of RNAs from triplicate experiments were pooled to exclude experimental bias. The total RNA was amplified and labeled using a Low RNA Input Linear Amplification kit PLUS (Agilent Technologies, Santa Clara, CA, USA) and then hybridized to a microarray (Agilent Human whole genome 44 K, Agilent Technologies) containing approximately 44,000 probes (approximately 21,600 unique genes) in accordance with the manufacturer’s instructions. The arrays were scanned using an Agilent DNA Microarray Scanner. The dataset is available online at the Gene Expression Omnibus (http://www.ncbi.nlm.nih.gov/geo) under the ID number GSE65413.

### Dose-dependent microarray analyses

The raw signal intensities were extracted from the arrays using Agilent Feature Extraction Software (Agilent Technologies). Only those array elements with signal intensities 1.4-fold higher than the local background were selected and normalized using the quantile method [7]. The ratios from duplicated spots were averaged. The expression ratios were hierarchically clustered with the average linkage method of the Gene Cluster 3.0 program (http://www.eisenlab.org/eisen/). For the identification of dose-dependent patterns of gene expression, the Short Time- series Expression Miner (STEM) program was used. Although STEM was developed for the time series analysis of microarrays, STEM can be applied for the identification of gene expression patterns from non-time-series microarrays [6]. The statistical significance of the resultant expression patterns were calculated as false discovery rates (FDRs) using 1,000 random permutations [8].

### Connectivity map

The dose-dependent genes that were up- or down-regulated in A549 cells after treatment with EEDS or helveticoside were used as up- and down-tags, respectively, for querying the Connectivity map 02 (http://www.broadinstitute.org/cmap/), which is composed of reference microarray data of 6,100 samples from diverse pharmaceutical substances, to perform non-parametric and rank-based pattern-matching algorithms based on the Kolmogorov-Smirnov statistic [9]. The list of genes used for the Connectivity map analysis is shown in Additional file 1 for EEDS and Additional file 2 for helveticoside. The query signature was compared to each rank-ordered list of reference microarray data to determine whether the up- and down-tags tended to appear near the top or bottom of the list, respectively, which yielded a connectivity score for each reference microarray. The statistical significance was then computed based on permutation.

### Gene ontology (GO) and pathway analyses

The Functional Annotation Tool of Database for Annotation, Visualization and Integrated Discovery (DAVID) was used for the identification of enriched GO terms and pathways using the dose-dependently expressed genes, and *p*-values were calculated using the modified Fisher’s exact test and adjusted using the Benjamini-Hochberg procedure [10]. To construct the functional network composed of non-redundant subsets of GO terms, the Reduce and Visualize Gene Ontology (REVIGO) program was used for the significantly enriched GO terms, and the distance between GO terms was based on semantic similarity [11].

For Network Ontology Analysis (NOA), we used a novel GO functional enrichment method (http://app.aporc.org/NOA), which was previously published for the network analysis by considering molecular interaction among gene products [12]. List of interacted genes from the network was used as an input in NOA.

For a systematic pathway analysis allowing for signaling pathway topology, we conducted a Signaling Pathway Impact Analysis (SPIA) in which a pathway is randomly bootstrapped 3,000 times to calculate two statistical values, namely P_NDE_ and P_PERT_, which represent the over‐representation of the input genes in a pathway and the abnormal perturbation of a specific pathway, respectively. Subsequently, the global *p*-value (P_G_) was calculated from P_NDE_ and P_PERT_ for the selection of significant pathways with multiple adjustments (P_GFDR_) [13].

To measure pathway activity, we linearly combined the logarithmic expression values of the genes in each pathway using a weight of −1 for repressors to account for the accumulative effect of all the genes in a pathway. The measured values were divided by the size of the pathway [14]. The statistical significance of the measured activity was estimated using a random permutation-based method (*n* = 1,000) in which the FDR was determined by comparing the original activity value with randomly permutated values [15]. The pathway information was obtained from the Kyoto Encyclopedia of Genes and Genomes (KEGG, http://www.genome.jp/kegg/) database.

### Functional network

A functional interaction network based on individual genes was constructed using the Reactome FI network Cytoscape plugin application (http://www.reactome.org/), which utilizes a database (2013 version) of protein-protein interactions, gene co-expression, protein domain interactions, GO annotations, and text-mined protein interactions [16]. Using the dose-dependently regulated genes with at least two-fold variation as the input, a Markov Cluster Algorithm (MCA) with a default inflation parameter of 5.0 was implemented using the Reactome FI program to cluster the networks. After the MCA step, the modules were selected by applying a default size of *n* = 7 and an average Pearson correlation coefficient of 0.8. The associations of the modules and GO terms were then measured using the REVIGO application [11].

The GO term network structure was also visualized using the Enrichment Map plugin for Cytoscape (http://baderlab.org/Software/EnrichmentMap/), wherein the connections between GO terms are based on the common genes of GO terms [17]. The results from the GO enrichment analysis from DAVID were used as inputs with the parameters *p*-value < 0.001, and default settings (FDR q-value < 0.1 and similarity coefficient cutoff of 0.5). For the implications of the cancer-related genes with enriched GO terms, known cancer genes were obtained from the DiseaseHub database (http://zldev.ccbr.utoronto.ca/~ddong/diseaseHub/), which provides a collection of disease-related genes from various databases, such as Online Mendelian Inheritance in Man (OMIM), Genetic Association Database (GAD), Human Gene Mutation Database (HGMD), Pharmacogenomics Knowledge Base (PharmGKB), Cancer Genome Project (CGP), and Genome Wide Association Studies (GWAS).

## Results

### Connectivity map for the effect of EEDS on A549 human lung cancer cells

**Fig. 1:**
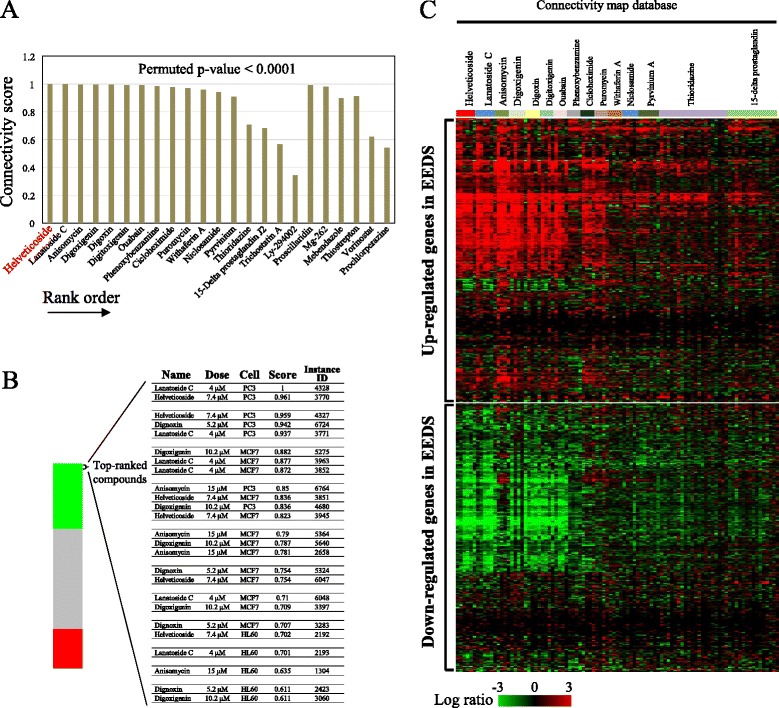
Enrichment analyses for EEDS using the Connectivity map. **a** The Connectivity map was queried using 275 up- and 193 down-regulated genes in A549 cells after EEDS treatment (Additional file 1) as up- and down-tags, respectively. The resultant connectivity scores of the 23 top-ranked chemicals (permutated *p*-value < 0.0001) were plotted in the order of rank. **b** The position of an individual treatment instance with the five top-ranked chemicals is plotted in the bar graph, which was constructed from 6,100 individual chemicals ordered by their corresponding connectivity scores from +1 (top) to −1 (bottom). The green, gray and red colors reflect the positive, null, and negative signs of the scores, respectively. The dose, cell line, connectivity score, and name (instance ID) of each individual instance included in the top five chemicals are shown. **c** A comparison of the EEDS-responsive gene expression profile with that obtained for several top-ranked chemicals is shown. The columns and rows represent individual samples and genes, respectively. The color scale for the expression ratio ranges from red (high) to green (low) as indicated by the scale bar.

We previously observed that EEDS treatment induced two major patterns of gene expression in A549 human lung cancer cells [6]. One pattern was composed of dose-dependently down-regulated genes, and the other pattern was composed of dose-dependently up-regulated genes. The former pattern was primarily involved in metabolic processes, and the latter pattern was primarily involved in signaling processes. However, the pharmacological activity of EEDS could not be clearly identified from this simple gene expression analysis. One way to connect the gene expression results to the potential pharmacology of EEDS would be to compare the gene expression pattern of EEDS to those of the vast majority of drugs with well-known chemical structures and pharmacology. Therefore, we utilized the Connectivity map of microarray data from cultured human cells treated with bioactive small molecules [9]. Fig. 1a shows the top-ranked drugs (ordered by increasing permuted *p*-values) with the most similar expression patterns to EEDS, as determined through an enrichment analysis of the Connectivity map, and helveticoside was ranked highest. Figure 1b presents the positions of an individual instance with the five top-ranked drugs, showing the treatment conditions used in the Connectivity map database For this analysis, we used 275 genes that were up-regulated (over 4-fold) and 193 genes that were down-regulated (under 0.25-fold) by EEDS. The list of genes used for the Connectivity map analysis is shown in Additional file 1. All 5 of the drugs, namely helveticoside, lanatoside C, anisomycin, digoxigenin, and digitoxigenin, showed high connectivity scores (greater than 0.6) under various experimental conditions. A list of the top 15 significantly enriched drugs is shown in Additional file 3. The similarity measurements based on gene expression demonstrated the close relationships between EEDS and the top-ranked chemicals (Fig. 1c). We confirmed that many of the genes that were up- or down-regulated by EEDS were also significantly up- or down-regulated by the top-ranked chemicals, respectively.

### Chemical profiling of EEDS

**Fig. 2:**
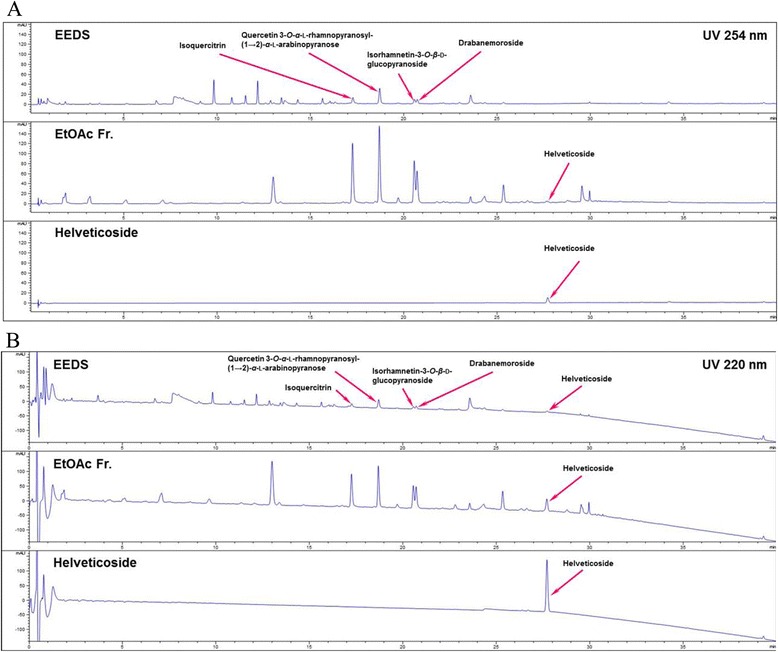
UHPLC chromatograms of EEDS and helveticoside. EEDS (80 % ethanol extract), ethyl acetate fraction of EEDS, and helveticoside were analyzed at (**a**) UV 254 nm and (**b**) 220 nm.

The above results imply the presence of specific chemical components, particularly glycosides, in EEDS. In fact, we have previously isolated diverse types of glycosides having cytotoxic and anti-inflammatory activities from EEDS [1]. Therefore, we investigated whether helveticoside, the top-ranked glycoside from the Connectivity map, was present in EEDS. Figure 2 shows the presence of peak of helveticoside in both fractions of *D. sophia;* 80 % ethanol fraction and EtOAc fraction of EEDS, as measured by UHPLC chromatograms at UV 254 nm (Fig. 2a) and 220 nm (Fig. 2b), respectively. After confirming the presence of helveticoside in EEDS, we then measured the biological activity of helveticoside in comparison with EEDS.

### Gene expression profiles induced by EEDS and helveticoside

**Fig. 3:**
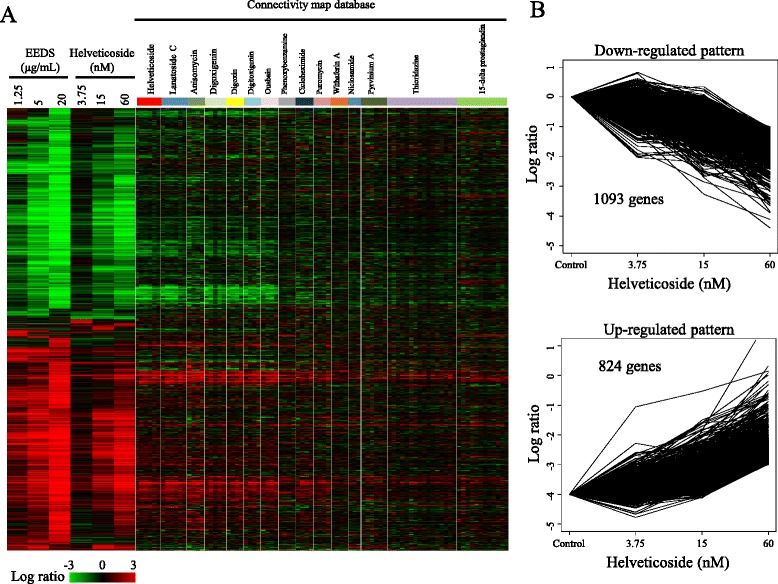
Dose-dependent gene expression after EEDS or helveticoside treatment in A549 cells. **a** Approximately 5,800 genes were differentially expressed over two-fold in at least one sample when compared with the vehicle control group. For comparison, the expression levels of genes induced by the top-ranked 15 chemicals selected from the Connectivity map analysis of EEDS are also displayed in parallel. The columns and rows represent individual samples and genes, respectively. The expression ratio color scale ranges from red (high) to green (low) as indicated by the scale bar. **b** The dose-dependently regulated genes affected by helveticoside treatment were identified with the STEM program (FDR < 0.001). The down-regulated pattern was composed of 1,093 genes, and the up-regulated pattern was composed of 824 genes.

To determine the effects of EEDS and helveticoside on cell growth, A549 human lung cancer cells in exponential growth phase were treated with each serially diluted drug (1.25, 5, and 20 μg/mL for EEDS and 3.75, 15, and 60 nM for helveticoside). The half maximal inhibitory concentrations (IC50s) of EEDS and helveticoside were 4.5 μg/mL and 35 nM, respectively. The overall patterns of gene expression after EEDS- or helveticoside treatment were compared in parallel with the top-ranked drugs from the Connectivity map database and are presented in Fig. 3a. As expected from the enrichment results of Fig. 1, EEDS and helveticoside regulated dose-dependent gene expression in a similar fashion. Two subgroups of genes that were up-regulated and down-regulated in a dose-dependent manner by EEDS and helveticoside were identified. These patterns of gene expression were also evident in the top-ranked drugs downloaded from the Connectivity map database, and these patterns were obtained irrespective of the individual treatment conditions, such as different cell lines, used for the top-ranked drugs from Connectivity map, as shown in Additional file 4. Moreover, the similarity of gene expression between the publicly available data and our experimental data on EEDS and helveticoside increased according to the rank.

**Fig. 4:**
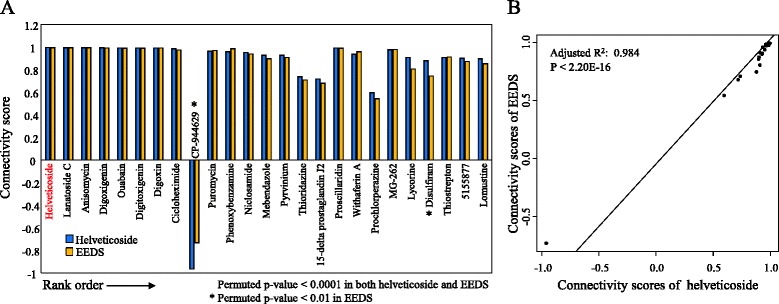
Comparison of connectivity scores between EEDS and helveticoside. **a** The 25 top-ranked drugs (permutated *p*-value < 0.0001) were selected from the Connectivity map analysis of helveticoside-treated A549 cells, in which 126 genes were up-regulated (over 4-fold) and 151 genes were down-regulated (under 0.25-fold). The connectivity scores were then compared with those obtained from the EEDS-treated A549 cells. The list of drugs is ordered according to the rank obtained from the Connectivity map analysis of helveticoside-treated A549 cells. **b** The correlation of the connectivity scores between EEDS and helveticoside was measured.

A quantitative dose-dependency analysis confirmed the presence of two distinctive patterns of gene expression after helveticoside treatment (FDR < 0.001) as follows: down- and up-regulated patterns consisting of 1,093 and 824 genes, respectively (Fig. 3b). As shown in the clustering profile, many of the same genes were observed in the two dose-responsive patterns after treatment with EEDS and helveticoside. Approximately 68.8 % (753/1,093) and 75.8 % (625/824) of the genes in the down-regulated and up-regulated patterns, respectively, of the cells that were treated with helveticoside responded similarly to EEDS treatment as shown in Additional file 5. In addition to the overall gene expression profile induced by helveticoside, we also performed the Connectivity map analysis using the helveticoside-responsive genes. Figure 4a shows the clear similarity between the enriched compounds in the Connectivity map analysis of helveticoside and EEDS. Among top-ranked 25 compounds enriched by helveticoside (permuted *p*-value < 0.0001), 23 compounds were also significantly enriched by EEDS (permuted *p*-value < 0.0001). A plot of the connectivity scores further confirmed the correlation between the results of EEDS and helveticoside (Fig. 4b). Interestingly, helveticoside itself was ranked highest by the Connectivity map analysis of our helveticoside data, which may validate our bioinformatic approach.

### GO analysis

The biological functions of the two expression patterns by treatment with helveticoside were investigated with GO analysis. As shown in Table 1, the down-regulated pattern was enriched with oxidation/reduction GO terms (GO:0055114, *p*-value < 0.001, FDR < 0.001). In contrast, signaling-related GO terms, including ‘apoptosis regulation’, ‘transcription regulation’, and ‘regulation of phosphate metabolic process’, were enriched in the up-regulated pattern (I-value < 0.001 and FDR < 0.001). The top 10 significantly enriched GO terms in the up-regulated pattern are shown in Table 1 (for the full list of enriched GO terms, please see Additional file 6). The GO terms in the up-regulated pattern were composed of hierarchically redundant terms. Thus, we eliminated the redundancy using the REVIGO program and obtained a network structure of non-redundant GO terms. Additional file 7 illustrates the inter-connected biological functions (*p*-value < 0.001 and FDR < 0.01) in the up-regulated pattern network. ‘apoptotic process’, ‘cell cycle’, ‘regulation of phosphate metabolism’, and ‘transcription’ were found to be interrelated. This functional enrichment in the up-regulated pattern was also confirmed by a text-based GO term distribution tree map (Additional file 7) in which related signaling functions, such as apoptosis, proliferation, and transcription, were predominately found in the up-regulated pattern. For the down-regulated pattern, we lowered the input stringency threshold (*p*-value < 0.01 and FDR < 0.1) to increase the number of input GO terms for the REVIGO analysis. The resultant GO term network and tree map showed that the down-regulated pattern is primarily associated with general metabolic processes, such as fatty acid metabolism, heterocycle biosynthesis, and DNA metabolism (Additional file 8). In addition, the overall distribution of all enriched GO terms (FDR < 0.01) across all samples including our EEDS and helveticoside experiments, and 15 top-ranked drugs from the Connectivity map database clearly shows the similar enrichment of GO terms among all datasets (Additional file 9), supporting biological similarity among datasets.

**Table 1:** Top 10 GO terms enriched (FDR < 0.01) by helveticoside

Pattern	GO ID	Name	*p*-value*	FDR^a^
Down-regulation	GO:0055114	Oxidation reduction	4.49E-08	1.13E-04
Up-regulation	GO:0042981	Regulation of apoptosis	9.16E-09	2.56E-05
	GO:0019220	Regulation of phosphate metabolic process	1.29E-08	1.79E-05
	GO:0051174	Regulation of phosphorus metabolic process	1.29E-08	1.79E-05
	GO:0043067	Regulation of programmed cell death	1.41E-08	1.31E-05
	GO:0010941	Regulation of cell death	1.61E-08	1.12E-05
	GO:0006357	Regulation of transcription from RNA polymerase II promoter	5.87E-08	3.28E-05
	GO:0042325	Regulation of phosphorylation	7.44E-08	3.46E-05
	GO:0007167	Enzyme linked receptor protein signaling pathway	1.32E-07	5.26E-05
	GO:0042127	Regulation of cell proliferation	2.59E-07	9.04E-05
	GO:0007242	Intracellular signaling cascade	7.04E-07	2.18E-04

*p-values were calculated using the Fischer’s test

^a^FDR corrections were calculated using the Benjamini-Hochberg procedure

### Pathway analyses

In addition to the GO analysis, we examined the functional involvement of the pathways in the helveticoside dose-responsive patterns using pathway enrichment analyses. Table 2 shows the enriched pathways (FDR < 0.01). Similar to the results of the GO analysis, all of the significantly enriched pathways (*p*-value < 0.001 and FDR < 0.01) were associated with the up-regulated pattern. Several signaling pathways, including MAPK pathway (KEGG 4010), TFG-beta pathway (KEGG 4350), circadian rhythm (KEGG 4710), and apoptosis pathway (KEGG 4210), were regulated by helveticoside. But, no pathways were found to be significantly associated with the down-regulated pattern.

**Table 2:** Pathways enriched (FDR < 0.01) by helveticoside

Pattern	KEGG ID	Name	*p*-value*	FDR^a^
Down-regulation	NA	NA	NA	NA
Up-regulation	hsa4010	MAPK signaling pathway	8.00E-07	1.10E-04
	hsa4350	TGF-beta signaling pathway	4.73E-06	3.25E-04
	hsa4710	Circadian rhythm	2.27E-05	1.03E-03
	hsa4210	Apoptosis	7.98E-05	2.71E-03
	hsa5200	Pathways in cancer	8.00E-05	2.18E-03
	hsa4540	Gap junction	8.86E-05	2.01E-03
	hsa5212	Pancreatic cancer	2.05E-04	3.96E-03
	hsa4060	Cytokine-cytokine receptor interaction	2.50E-04	4.22E-03
	hsa5219	Bladder cancer	3.39E-04	5.07E-03
	hsa4115	p53 signaling pathway	4.73E-04	6.33E-03
	hsa4660	T cell receptor signaling pathway	5.15E-04	6.27E-03
	hsa5221	Acute myeloid leukemia	5.31E-04	5.94E-03
	hsa5211	Renal cell carcinoma	5.74E-04	5.93E-03
	hsa4110	Cell cycle	6.00E-04	5.76E-03
	hsa4621	NOD-like receptor signaling pathway	8.03E-04	7.15E-03
	hsa4662	B cell receptor signaling pathway	9.02E-04	7.52E-03

*p-values were calculated using the Fischer’s test

^a^FDR corrections were calculated using the Benjamini-Hochberg procedure

**Fig. 5:**
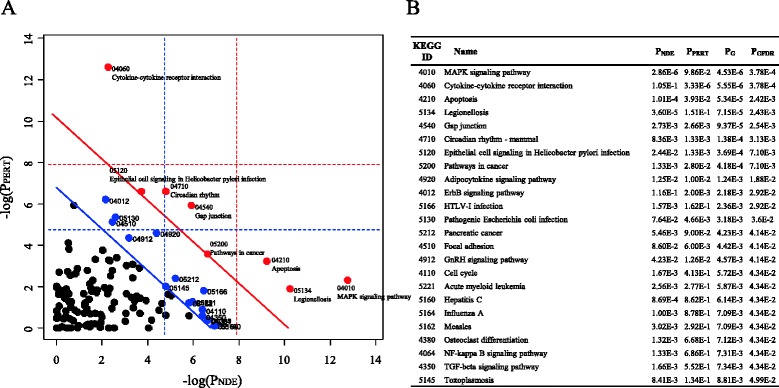
Pathways altered after helveticoside treatment in A549 cells. **a** The pathways involved in the up-regulated pattern (824 genes) and down-regulated pattern (1,093 genes) were analyzed with the SPIA program. The horizontal axis represents pathway over-representation (P_NDE_), while the vertical axis indicates pathway perturbation (P_PERT_). The dotted horizontal and vertical lines represent the corrected thresholds (1 %) of significance (red for Bonferroni and blue for FDR correction) for each axis value. The red and blue circles located to the right of the oblique lines are the significant pathways (red circles for P_GFDR_ < 0.01, and blue circles for P_GFDR_ < 0.05) with the KEGG IDs after the FDR correction of the global *p*-values (P_Gs_)_,_ which were calculated from the combined probabilities of P_NDE_ and P_PERT_. The list of pathways for the red circles (P_GFDR_ < 0.01) is shown in (**b**).

For a more systematic analysis of the pathways, we conducted a SPIA pathway analysis, which calculates the connections of pathways by considering their topology and the expression levels of their genes. Figure 5a shows 8 pathways (red circles) that were significantly enriched (P_G_ < 0.01 and P_GFDR_ < 0.01), including MAPK pathway, cytokine-cytokine receptor interaction pathway, circadian rhythm and apoptosis pathway. The blue circles in Fig. 5a represent marginally significant pathways (P_GFDR_ < 0.05). All of these pathways were related to signaling or diseases, which is consistent with the simple enrichment results shown in Table 2. The statistical significances of these pathways are listed in Fig. 5b.

**Fig. 6:**
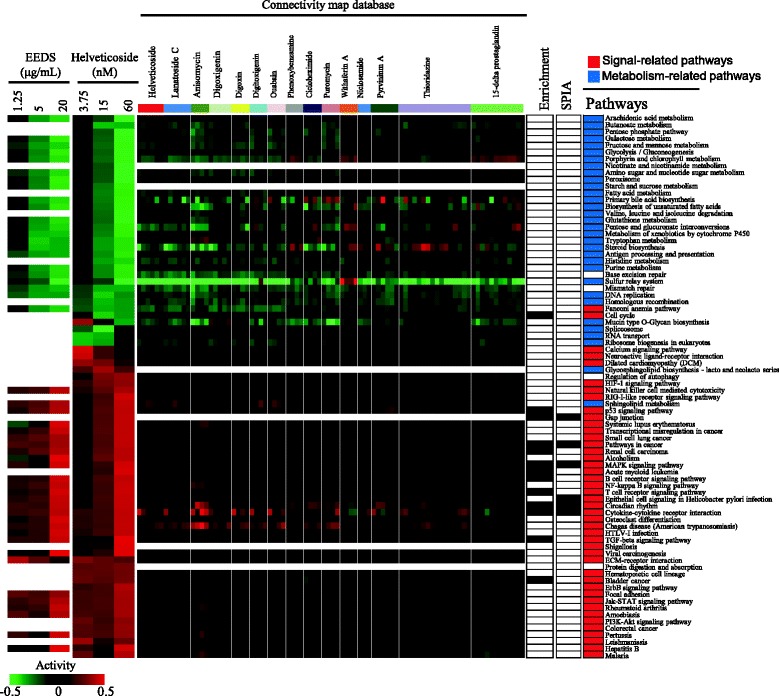
Dose-dependent changes in pathway activity after helveticoside treatment in A549 cells. The pathway activities (FDR < 0.01), which were calculated by linear combinations of gene expression, were hierarchically clustered. For comparison, the statistically significant pathways (FDR < 0.01) after EEDS treatment are shown in parallel. For comparison, the pathway activity obtained for the 15 top-ranked chemicals selected from the Connectivity map analysis of EEDS is also displayed in parallel. The columns represent individual samples, and the rows represent pathways. The red and green colors reflect high and low activity levels, respectively, as indicated by the scale bar with arbitrary units. The pathways that were enriched based on the enrichment analysis (Table 2) and SPIA (Fig. 5) are also indicated in black in the right panel of the pathway activity bar. Signal-related pathways and metabolism-related pathways are colored red and blue, respectively, in the right panel.

In addition to the identification of enriched pathways, we also investigated dose-dependent changes in pathway activity, which were measured by linearly combining the expression values of the genes in each pathway. Figure 6 shows the dose-dependent changes in 80 statistically significant pathways (FDR < 0.01) after helveticoside treatment. Interestingly, similar to the patterns of overall gene expression, the pathways were also divided into two major pattern groups based on their activity and the top-ranked drugs in the Connectivity map also show a similar pattern of pathway activities. As obtained from the analysis of gene expression, the individual treatment conditions, such as different cell lines, used for the analysis of the top-ranked drugs in the connectivity map has no significant effect, as shown in Additional file 10.

Furthermore, the dose-dependently down-regulated pathways were generally composed of general metabolism-related pathways, while the up-regulated pathways were composed of signaling or disease-related pathways. These patterns of pathway activity were also observed after EEDS treatment. We also compared the pathways selected from the SPIA analysis and the simple pathway enrichment analysis with the pathway activities in Fig. 6.

### Network-based functional analyses

**Fig. 7:**
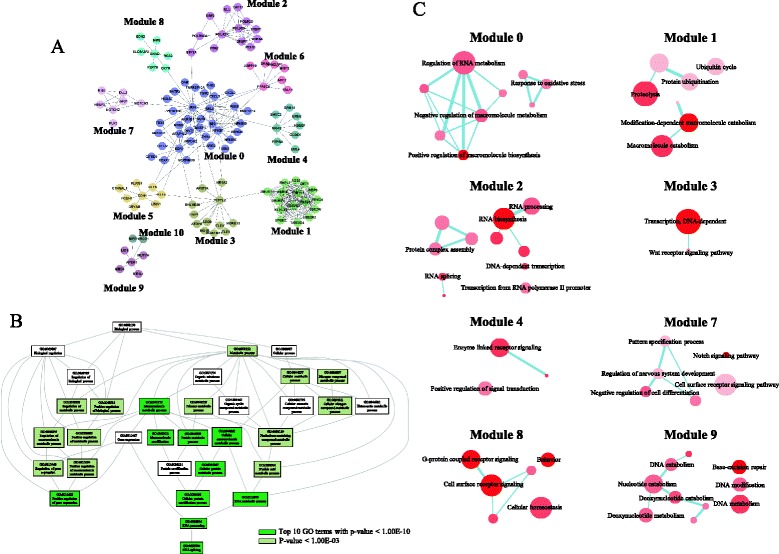
Interaction network of the genes induced by helveticoside in A549 cells. **a** The interaction network was constructed from 1,093 genes with the down-regulated pattern and 824 genes with the up-regulated pattern by implementing the Reactome FI application. In total, 11 modules (as indicated from 0 to 10) are shown in different colors. **b** The GO terms associated with this interaction network were analyzed by NOA, and top 10 enriched GO terms (*p*-value < 1.00E-10) were measured in the subgraph of GO-directed acyclic structure. **c** The network structure of the enriched GO terms in each module (FDR < 0.01) was obtained from the REVIGO program. The node size and color intensity are proportional to the hierarchical status and statistical significance of each node, respectively. The edge thickness between two nodes represents their closeness.

The co-expression of functionally associated genes suggested the presence of an interrelated network of genes that could be induced by external stimuli, such as helveticoside or EEDS. Although the pathway and GO information provided one of these types of networks, we used a more comprehensive functional interaction database (Reactome FI) [16] to obtain functional subgroup networks composed of co-expressed genes induced by helveticoside treatment. Using 1,093 and 824 genes from the down- and up-regulated patterns, respectively, we constructed a network of genes composed of 11 modules (from 0 to 10), shown in Fig. 7a. The GO terms associated with co-expression networks were measured by NOA, which incorporates the interaction status of the gene products for the selection of enriched GO terms. The resulting top 10 enriched GO terms (*p*-value < 10E-10) shows that metabolic processes involving protein metabolism and nucleotide metabolism were significantly enriched in response to helveticoside in the subgraph of GO-directed acyclic structure (Fig. 7b). In addition, various signaling processes, including gene expression regulation and cell development and differentiation, were also significantly enriched. The full list of significantly enriched GO terms in the NOA is shown in Additional file 11, and the detailed GO terms associated with each module are shown in Fig. 7c. In accordance with NOA, signaling functions, such as transcription pathways (module 3), receptor signaling (modules 4 and 8), and the notch signaling pathway (module 7), were significantly associated with each module. In addition to signaling pathways, cellular metabolism functions were also enriched in the modules, including macromolecule biosynthesis (module 0), catabolism (module 1), RNA metabolism (module 2), and nucleotide metabolism (module 9). A text-based GO term distribution tree map for each module is shown in Additional file 12. The individual genes included in each module from the entire network are listed in Additional file 13 along with detailed network characteristics representing the centrality of each node. The module network structures showed that the diverse functions enriched in the GO and pathway analyses were interrelated through modules.

The interrelationship of the biological functions regulated by helveticoside treatment was further verified by the GO term enrichment map. While relationships between GO terms based solely on the GO hierarchy, the network from the enrichment map was based on the genes included in the GO terms. As shown in Additional file 14, the signaling (kinase activity and cell migration) and metabolic functions were interconnected, thus implying the presence of a common response to these two cellular processes after helveticoside treatment. Because a number of cardiac glycosides have been shown to exhibit anti-proliferative effects on tumors [18–20], and because our previous report also showed that EEDS could be used as an anti-cancer agent [6], we measured whether diverse cancer types could be associated with the biological network regulated by helveticoside treatment. Interestingly, the genes related to diverse types of cancers, through modules.including colon cancer, breast cancer, kidney cancer, and gastric cancer, were associated with biological functions that were enriched after helveticoside treatment as shown in Additional file 15, thereby supporting the assumption that helveticoside could be effective against diverse cancer types.

## Discussion

Identification of biologically active components is crucial for the development of novel drugs from herbal extracts. However, determining which component has crucial pharmacological activity requires great effort and a significant time commitment when using conventional approaches. Moreover, it is challenging to infer the pharmacological effect of a component using *in vitro* experiments alone. Therefore, it is important to reduce this time-consuming process in advance and efficiently narrow down the possible candidates for subsequent screening from the complex chemical components of herbal extracts.

The application of genomic approaches is one way to reduce the time required for *in vitro* screening. The Connectivity map resource is a reference collection of gene expression profiles from cultured human cells treated with diverse bioactive small molecules. Using the Connectivity map, the mechanisms of action, physiological processes, and disease associations of unknown substances can be predicted. As evidenced by the results of various experiments, the pattern-match algorithm implemented in the Connectivity map, which adopts a non-parametric, rank-based method using the Kolmogorov-Smirnov statistic (as described in Gene Set Enrichment Analysis (GSEA) [21]), minimizes the effect of different experimental conditions, such as cell type, drug concentration, and treatment period [9].

In the present study, we queried the Connectivity map with a list of genes (293 up-regulated and 275 down-regulated genes treated with EEDS in A549 cells as shown in Additional file 1). Among the top-ranked drugs, many compounds, such as helveticoside, lanatoside, digoxigenin, digoxin, digitoxigenin and ouabain, are classified into a cardiac glycoside family sharing a common chemical structure. These compounds showed highly similar gene expression patterns with EEDS. Interestingly, we identified that helveticoside was one of the cytotoxic components of EEDS. Many of the genes regulated by helveticoside were also regulated by EEDS in the present study, thus implying that helveticoside could be one of the leading biologically active components of EEDS. Moreover, the Connectivity map analysis using helveticoside-responsive genes from our experiment also successfully identified helveticoside as the top-chemical from the Connectivity map database. However, this result does not exclude the possible presence of other cardiac glycosides in EEDS, although we could not identify other cardiac glycosides mentioned above than helveticoside in EEDS.

Helveticoside is a cardiac glycoside that has a similar chemical structure as estrogens. Cardiac glycosides have been used for many years for the treatment of cardiac congestion and arrhythmias [22]. In addition, cardiac glycosides have also been proven to have anti-proliferative activity on tumors [18–20]. The possible mechanisms of their anti-cancer effect include the ability of cardiac glycosides to bind to estrogen receptors or inhibit Na+/K+ ATPase activity [20, 23]. We identified that the biological functions regulated by helveticoside treatment were associated with diverse cancer types in terms of biological function, which implies the potential usefulness of helveticoside and/or EEDS as anti-cancer agents.

As evidenced by the GO and pathway analyses, the down-regulated genes in helveticoside-treated cells were associated with metabolic processes, and the up-regulated genes in helveticoside-treated cells were involved in signaling processes, which is consistent with our previous results with EEDS [6]. This reciprocal regulatory mechanism may provide clues for understanding the growth inhibitory mechanism of EEDS and helveticoside in A549 cancer cells. For example, metabolic processes, such as the pentose phosphate pathway and the excision repair pathway, which were enriched by EEDS, can regulate lung cancer cells and are associated with lung cancer risks [24, 25]. Furthermore, these pathways can modulate the effectiveness of chemotherapy in lung cancer patients [26]. In addition, a recent study has suggested that the regulation of oxidation/reduction pathways, which were the same pathways associated with down-regulated genes after helveticoside treatment in our study, is a promising systemic target for cancer treatments [27], thus supporting a possible role for helveticoside as an anti-cancer agent. Additionally, several signaling pathways, such as the apoptosis and p53 pathways, are the targets of herbal-derived anti-lung cancer drugs [28–30].

In the present study, the reciprocal regulation between metabolic and signaling processes was more evident in the pathway activity analysis (Fig. 6). The dose-dependently down-regulated pathways were predominantly composed of metabolism pathways, and the up-regulated pathways were exclusively composed of signaling or disease-related pathways. Interestingly, the tight linkage between metabolism and signaling is becoming increasingly clear in a variety of cellular conditions in which protein modification by acetylation, glycosylation, and phosphorylation is thought to play an important role during reciprocal regulation [31, 32]. However, a functionally reciprocal response induced by drug treatment has not been previously reported. Moreover, our present results showed that two reciprocally regulated biological processes are connected in a functional network structure, thus signifying a possible linkage between metabolic and signaling processes. The biological significance of these functional network associations must be further verified in terms of the pharmacological effects of helveticoside.

## Conclusions

In summary, using the Connectivity map, we have identified that helveticoside induced a reciprocal regulation of genes and biological functions in A549 cells and that it could be the foremost biologically active component of EEDS.

### Availability of supporting data

The data sets supporting the results of this article are included within the article and its additional files. Microarray data are available in the Gene Expression Omnibus (http://www.ncbi.nlm.nih.gov/geo) under accession number GSE65413.

## Additional files

Additional file 1:
**List of genes used in the Connectivity map analysis for EEDS.** (PDF 112 kb) Additional file 2:
**Full list of genes in each pattern after treatment with helveticoside.** (PDF 370 kb) Additional file 3:
**Top 15 chemicals with high connectivity scores in the analysis of EEDS.** (PDF 67 kb) Additional file 4:
**Comparison of gene expression profiles.** The gene expression profiles obtained with our EEDS and helveticoside experiments and for the seven top-ranked drugs selected from the Connectivity map database were compared. For the seven top-ranked drugs, the cell lines and drug concentrations used in the experiment are indicated with different colors. (PDF 145 kb) Additional file 5:
**Comparison of the dose-dependently regulated genes after treatment with helveticoside and EEDS.** (PDF 53 kb) Additional file 6:
**GO terms enriched (FDR < 0.01) by helveticoside.** (PDF 69 kb) Additional file 7:
**Enriched GO terms in the up-regulated pattern after treatment of A549 cells with helveticoside.** (A) The network structure was constructed with non-redundant GO terms that were enriched in the up-regulated pattern (FDR < 0.01) after helveticoside treatment using the REIVGO program. The node color indicates the statistical significance, and the node size is proportional to the frequency of the GO term in the underlying GOA database. Highly correlated GO terms are linked by edges, the thickness of which indicates the degree of similarity. (B) Tree map structures composed of non-redundant GO terms (FDR < 0.01) enriched in the up-regulated pattern after helveticoside treatment were constructed. In the tree structures, closely related terms share the same color. The size of each GO term is proportional to its level of statistical significance. (PDF 88 kb) Additional file 8:
**Enriched GO terms in the down-regulated pattern after treatment of A549 cells with helveticoside.** The network structure was constructed from the non-redundant GO terms enriched in the down-regulated pattern after helveticoside treatment using the REIVGO program. We lowered the input stringency threshold to *p*-value < 0.01 to increase the number of GO input terms. The node size and color intensity are proportional to the hierarchical status and statistical significance of each node, respectively. The edge thickness between nodes represents the closeness of the two nodes. In the tree structure, closely related terms share the same color. The size of each GO term is proportional to its level of statistical significance. (PDF 89 kb) Additional file 9:
**Distribution of enriched GO terms (FDR < 0.01) across all of the samples, including our EEDS and helveticoside experiments and the 15 top-ranked drugs from the Connectivity map database.** (PDF 86 kb) Additional file 10:
**Dose-dependent changes in pathway activity.** (PDF 105 kb) Additional file 11:
**Full list of significantly enriched GO terms determined through Network Ontology Analysis (NOA).** (PDF 77 kb) Additional file 12:
**Tree map structure of the GO terms enriched in each module of Fig.** **7c** **.** Closely related GO terms share the same color. The size of each GO term is proportional to its level of statistical significance. (PDF 143 kb) Additional file 13:
**Genes included in each module of network structure.** (PDF 121 kb) Additional file 14:
**Enrichment map analysis of the genes induced by helveticoside treatment in A549 cells.** The network structure of the GO terms was constructed by implementing the Enrichment map plugin for Cytoscape. The simple GO enrichment results from 1,093 genes with the down-regulated pattern and 824 genes with the up-regulated pattern were used as input data. Each node represents a GO term sized proportionally to the number of genes encompassed by that GO term. The edge thickness signifies the closeness of the two nodes measured by their number of common genes. The representative biological functions for the groups of nodes are shown. (PDF 713 kb) Additional file 15:
**Association of GO terms with the cancer-related genes in the Enrichment map.** The association of GO terms with the cancer-related genes is shown, and the blue diamond in the center represents the cancer-related genes obtained from the DiseaseHub database. A yellow node indicates a GO term with cancer-related genes, and the thickness of the purple edge is proportional to the number of genes. (PDF 6877 kb)

## Abbreviations

DAVID: Database for Annotation, Visualization, and Integrated Discovery
EEDS: Ethanol extract of *D. sophia* seeds
EtOAc: Ethyl acetate
FDR: False discovery rate
GO: Gene Ontology
KEGG: Kyoto Encyclopedia of Genes and Genomes
NOA: Network Ontology Analysis
REVIGO: Reduce and Visualize Gene Ontology
SPIA: Signaling Pathway Impact Analysis
STEM: Short Time-series Expression Miner
UHPLC: Ultra high performance liquid chromatography
