# Supplementary material for: Helveticoside is a biologically active component of the seed extract of Descurainia sophia and induces reciprocal gene regulation in A549 human lung cancer cells
Source: BMC Genomics. 2015 Sep 18;16(1):713. doi: 10.1186/s12864-015-1918-1 (PMC4575430; doi:10.1186/s12864-015-1918-1)
Supplement: Additional file 1: — List of genes used in the Connectivity map analysis for EEDS. (PDF 112 kb) [file 12864_2015_1918_MOESM1_ESM.pdf]

Additional file 1. List of genes used in the Connectivity map analysis for EEDS.

Up-regulated genes by EEDS			Down-regulated genes by EEDS		
**Symbol**	**Entrez ID**	**Fold (Log2)**	**Symbol**	**Entrez ID**	**Fold (Log2)**
EGR1	1958	10.0	HOXB13	10481	-5.0
FOSB	2354	7.8	ST6GAL1	6480	-4.8
EGR2	1959	6.4	GPRIN2	9721	-4.7
ATF3	467	5.6	SALL2	6297	-4.7
PPP1R15A	23645	5.5	CBR3	874	-4.5
IL8	3576	5.5	FANCF	2188	-4.5
MAFF	23764	5.0	CCR7	1236	-4.4
C3orf52	79669	4.9	VAV3	10451	-4.3
DDIT3	1649	4.9	C14orf93	60686	-4.3
FOS	2353	4.8	NTHL1	4913	-4.3
LTB	4050	4.8	VASH1	22846	-4.2
GADD45B	4616	4.8	TCEA2	6919	-4.2
ARC	23237	4.7	HSPA8	3312	-4.2
PER1	5187	4.6	B4GALNT1	2583	-4.1
SERPINE1	5054	4.5	DIXDC1	85458	-4.0
HBEGF	1839	4.4	NUDT18	79873	-4.0
TNFAIP3	7128	4.3	SYT17	51760	-4.0
FOSL1	8061	4.3	ACOX2	8309	-3.9
LCAT	3931	4.3	C3orf18	51161	-3.9
DLX2	1746	4.2	SMO	6608	-3.8
KLF4	9314	4.1	ELAC1	55520	-3.8
GREM1	26585	4.1	NNMT	4837	-3.8
BIRC3	330	4.1	MAGEH1	28986	-3.7
C10orf110	55853	4.0	BCAS1	8537	-3.7
AOC3	8639	4.0	CNPY2	10330	-3.7
GADD45A	1647	4.0	MAP2K6	5608	-3.6
KDM6B	23135	3.9	MTMR4	9110	-3.6

CYR61	3491	3.8	KRCC1	51315	-3.6
STX3	6809	3.8	SETBP1	26040	-3.5
EREG	2069	3.8	FHOD3	80206	-3.5
DNAJB2	3300	3.8	B3GALT4	8705	-3.5
CYP1A1	1543	3.7	HGD	3081	-3.5
PDGFB	5155	3.7	EHMT2	10919	-3.4
CSF2	1437	3.7	THNSL1	79896	-3.4
UGCG	7357	3.7	PDE8B	8622	-3.4
DAAM1	23002	3.6	EPB41L4A	64097	-3.4
EDN2	1907	3.6	HPS6	79803	-3.4
AEN	64782	3.6	TST	7263	-3.3
MCTP1	79772	3.6	HS3ST1	9957	-3.3
RASSF1	11186	3.6	CTDSPL	10217	-3.2
NFKBIE	4794	3.6	MANSC1	54682	-3.2
TNFRSF12A	51330	3.5	SMARCAL1	50485	-3.2
SMOX	54498	3.5	PIK3C2B	5287	-3.2
MAP3K14	9020	3.5	IDH1	3417	-3.2
GEM	2669	3.5	PDE7B	27115	-3.2
MXD1	4084	3.4	DOK1	1796	-3.2
CCL20	6364	3.4	RHOBTB1	9886	-3.2
CCRN4L	25819	3.4	PBX1	5087	-3.2
ASMT	438	3.4	MORC4	79710	-3.2
PLA2G4C	8605	3.4	C17orf108	201229	-3.2
BBC3	27113	3.3	ADAT1	23536	-3.1
AREG	374	3.3	RNF43	54894	-3.1
IL11	3589	3.3	KCNJ6	3763	-3.1
ETS1	2113	3.3	ZC4H2	55906	-3.1
JUN	3725	3.3	SYT12	91683	-3.1
BTG2	7832	3.3	ZNF362	149076	-3.1
TXNL4B	54957	3.3	TRIM45	80263	-3.0
ANKRD1	27063	3.3	CAPN5	726	-3.0

TMEM156	80008	3.3	MGAT3	4248	-3.0
EPHA2	1969	3.3	PCYT2	5833	-3.0
HAS2	3037	3.2	ABCA3	21	-3.0
ADM	133	3.2	PLCD1	5333	-3.0
CDKN1A	1026	3.2	HSPA2	3306	-3.0
SOCS3	9021	3.2	PRIM1	5557	-2.9
TNFRSF10D	8793	3.1	ZNF32	7580	-2.9
OXTR	5021	3.1	DENND2D	79961	-2.9
NFKB2	4791	3.1	LARGE	9215	-2.9
DUSP10	11221	3.1	LXN	56925	-2.9
C1orf69	200205	3.1	SNPH	9751	-2.9
FAM193B	54540	3.0	RASSF4	83937	-2.9
LOC157562	157562	3.0	THYN1	29087	-2.9
CCNL1	57018	3.0	FAM55C	91775	-2.8
C19orf61	56006	3.0	LRRC20	55222	-2.8
ARID3B	10620	3.0	SLC24A6	80024	-2.8
SDC4	6385	3.0	NUDT7	283927	-2.8
MAPK8IP3	23162	3.0	PNMAL1	55228	-2.8
GABARAPL1	23710	3.0	FAM134B	54463	-2.8
KPNA5	3841	3.0	GOLSYN	55638	-2.8
IL1RAP	3556	3.0	C16orf42	115939	-2.8
EMP1	2012	2.9	CYB5B	80777	-2.8
THAP3	90326	2.9	GNAZ	2781	-2.8
CXCL1	2919	2.9	GMPPA	29926	-2.8
ADRB2	154	2.9	KIAA0485	57235	-2.8
TRAF1	7185	2.9	PRMT7	54496	-2.7
CTSK	1513	2.9	C16orf59	80178	-2.7
TBX21	30009	2.9	HOXA6	3203	-2.7
CTGF	1490	2.9	C5orf54	63920	-2.7
CLCF1	23529	2.9	FBXO4	26272	-2.7
IL32	9235	2.9	STAT6	6778	-2.7

LYST	1130	2.8	GPR162	27239	-2.7
KLF10	7071	2.8	THAP7	80764	-2.7
GATA6	2627	2.8	JMJD4	65094	-2.7
SERPINB8	5271	2.8	SYTL2	54843	-2.7
RIT1	6016	2.8	ARRB1	408	-2.6
REL	5966	2.8	CYP2U1	113612	-2.6
RSRC2	65117	2.8	RGL1	23179	-2.6
FOXD1	2297	2.8	HOXC4	3221	-2.6
RUNX1	861	2.8	SLC22A3	6581	-2.6
PLK3	1263	2.8	CBR1	873	-2.6
NFKBIB	4793	2.8	GEMIN6	79833	-2.6
MAP2K3	5606	2.8	KHK	3795	-2.6
PTHLH	5744	2.8	ADRA1D	146	-2.6
ZNF484	83744	2.8	IL22RA1	58985	-2.6
NAB2	4665	2.7	MYLK	4638	-2.6
RELB	5971	2.7	USP9X	8239	-2.6
CRY1	1407	2.7	CCDC106	29903	-2.6
ZNF136	7695	2.7	IFIT1	3434	-2.6
JUNB	3726	2.7	SAC3D1	29901	-2.5
PPP1R13L	10848	2.7	ZMYND8	23613	-2.5
STK19	8859	2.7	MIIP	60672	-2.5
C14orf138	79609	2.7	MUTYH	4595	-2.5
RSC1A1	6248	2.7	LCMT2	9836	-2.5
ARHGAP19	84986	2.7	MSTO1	55154	-2.5
LIF	3976	2.6	TRADD	8717	-2.5
STC1	6781	2.6	APBA2	321	-2.5
IGF2BP2	10644	2.6	BFSP1	631	-2.5
YRDC	79693	2.6	RNASEL	6041	-2.5
IFRD1	3475	2.6	HS6ST1	9394	-2.5
JMJD1C	221037	2.6	DET1	55070	-2.5
TAF1A	9015	2.6	IMPA2	3613	-2.5

TLE4	7091	2.6	USP18	11274	-2.5
POFUT2	23275	2.6	PRKCDBP	112464	-2.5
OVGP1	5016	2.6	MTUS1	57509	-2.5
PKP2	5318	2.6	MAGED4B	81557	-2.5
C10orf118	55088	2.6	LRFN3	79414	-2.5
SLC19A2	10560	2.6	ZNF232	7775	-2.5
ELL	8178	2.6	FAM102A	399665	-2.5
PPM1D	8493	2.6	RIBC2	26150	-2.5
RLF	6018	2.6	C7orf68	29923	-2.5
CHD2	1106	2.6	RHOBTB2	23221	-2.5
UPF3B	65109	2.6	EHD3	30845	-2.5
PFKFB4	5210	2.6	FZD4	8322	-2.4
IER5	51278	2.5	QARS	5859	-2.4
GUCA1B	2979	2.5	PYCARD	29108	-2.4
HRK	8739	2.5	AZI1	22994	-2.4
CCNT2	905	2.5	FUT8	2530	-2.4
FBXL12	54850	2.5	OSBPL7	114881	-2.4
STARD13	90627	2.5	HPGD	3248	-2.4
DOHH	83475	2.5	RDBP	7936	-2.4
SLMO1	10650	2.5	KRT4	3851	-2.4
TICAM1	148022	2.5	ATP13A2	23400	-2.4
ZNF654	55279	2.5	CFB	629	-2.4
EFNA1	1942	2.5	TBC1D16	125058	-2.4
GJB3	2707	2.5	FBXO9	26268	-2.4
CDKN2AIP	55602	2.5	NDRG3	57446	-2.4
DUSP1	1843	2.5	HEATR3	55027	-2.4
ZNF143	7702	2.5	SPATA7	55812	-2.4
IER2	9592	2.5	MMP15	4324	-2.4
TUBA4A	7277	2.5	PIR	8544	-2.4
KLHL28	54813	2.5	MPI	4351	-2.4
PRDM1	639	2.4	TTC12	54970	-2.4

ACVR2A	92	2.4	DPYSL3	1809	-2.4
ZNF335	63925	2.4	KCNMB4	27345	-2.3
PI4K2A	55361	2.4	TTLL1	25809	-2.3
ID2	3398	2.4	SIDT2	51092	-2.3
PRPF39	55015	2.4	IMPACT	55364	-2.3
FERMT2	10979	2.4	MAP4K2	5871	-2.3
GADD45G	10912	2.4	FCHO1	23149	-2.3
KLF6	1316	2.4	LOC644450	644450	-2.3
MORC3	23515	2.4	UBAC1	10422	-2.3
TUBA8	51807	2.4	TENC1	23371	-2.3
VEGFA	7422	2.4	TREX1	11277	-2.3
EFNB2	1948	2.4	PTPN18	26469	-2.3
MOSPD1	56180	2.4	RBBP9	10741	-2.3
GNLY	10578	2.4	MYH10	4628	-2.3
C6orf35	729515	2.4	RGS14	10636	-2.3
RBBP6	5930	2.4	PLCH1	23007	-2.3
GDF15	9518	2.4	SLC9A3R2	9351	-2.3
RRAD	6236	2.4	APITD1	378708	-2.3
DDX3Y	8653	2.4	CPOX	1371	-2.3
TUBA3C	7278	2.4	ZDHHC6	64429	-2.3
CRY2	1408	2.4	PACS2	23241	-2.3
TOP1	7150	2.4	RPL23AP32	56969	-2.3
NFAT5	10725	2.4	INTS3	65123	-2.3
CCKBR	887	2.4	NRG2	9542	-2.3
ROR1	4919	2.4	NT5M	56953	-2.3
CLDN1	9076	2.4	ITPKB	3707	-2.3
SSH1	54434	2.4	RSAD1	55316	-2.2
C1orf63	57035	2.4	PTDSS1	9791	-2.2
TERF2IP	54386	2.4	NAT1	9	-2.2
FOXD3	27022	2.3	NEURL	9148	-2.2
TUFT1	7286	2.3	PIK3R2	5296	-2.2

ABL2	27	2.3	SUOX	6821	-2.2
ID4	3400	2.3	RGS10	6001	-2.2
TNFRSF10B	8795	2.3	ZNF839	55778	-2.2
OTUD3	23252	2.3	RGS19	10287	-2.2
GPRC5B	51704	2.3	MPST	4357	-2.2
KIAA0895	23366	2.3	PCYOX1L	78991	-2.2
AKAP12	9590	2.3	ACADSB	36	-2.2
HMGCS1	3157	2.3	MRPS34	65993	-2.2
CRABP2	1382	2.3	CUL9	23113	-2.2
PIM1	5292	2.3	GSDMD	79792	-2.2
HIVEP1	3096	2.3	GAB2	9846	-2.2
RHBDF1	64285	2.3	FLJ11710	79904	-2.2
STRN3	29966	2.3	KAT2A	2648	-2.2
TSC22D2	9819	2.3	TUB	7275	-2.2
AMOTL2	51421	2.3	DGKG	1608	-2.2
ARID3A	1820	2.3	LHX2	9355	-2.2
SLC35F2	54733	2.3	TMEM143	55260	-2.2
SLC7A6	9057	2.3	PDCD2	5134	-2.2
VPS37B	79720	2.3	FERMT1	55612	-2.2
ZFP36	7538	2.3	FLJ11235	54508	-2.2
C1GALT1	56913	2.3	DOK4	55715	-2.2
BTG1	694	2.3	ARHGEF9	23229	-2.2
LONRF3	79836	2.2	F12	2161	-2.1
CPEB3	22849	2.2	UBE2D4	51619	-2.1
ZNF529	57711	2.2	C8orf55	51337	-2.1
CDC42SE1	56882	2.2	RWDD2B	10069	-2.1
IRF1	3659	2.2	PLD2	5338	-2.1
NR1D2	9975	2.2	TUBBP5	643224	-2.1
THAP9	79725	2.2	KIAA0100	9703	-2.1
DUSP6	1848	2.2	RAB15	376267	-2.1
NEK7	140609	2.2	ZCWPW1	55063	-2.1

SCML2	10389	2.2	TMC5	79838	-2.1
PRKAB1	5564	2.2	OSBPL1A	114876	-2.1
GABPB1	2553	2.2	TBCD	6904	-2.1
CXCL3	2921	2.2	NARG2	79664	-2.1
C6orf145	221749	2.2	CLDN3	1365	-2.1
CHIC2	26511	2.2	AMDHD2	51005	-2.1
TEF	7008	2.2	F8A1	8263	-2.1
THAP1	55145	2.2	GALNT4	8693	-2.1
UNC84B	25777	2.2	WDR4	10785	-2.1
F3	2152	2.2	C19orf54	284325	-2.1
MAFK	7975	2.2	GINS4	84296	-2.1
SKIL	6498	2.2	FANCG	2189	-2.1
UFM1	51569	2.2	ARHGAP22	58504	-2.1
NPR2	4882	2.2	BDH1	622	-2.1
CAPRIN2	65981	2.2	XRCC1	7515	-2.1
RFPL3S	10737	2.2	SMARCD3	6604	-2.1
SNIP1	79753	2.2	ANXA4	307	-2.1
FST	10468	2.2	CDON	50937	-2.1
PPL	5493	2.2	CCDC71	64925	-2.1
PVR	5817	2.2	MLPH	79083	-2.1
ZCCHC6	79670	2.2	ETV6	2120	-2.1
ZEB2	9839	2.1	C8orf51	78998	-2.1
TLR6	10333	2.1	C14orf133	63894	-2.1
ZNF460	10794	2.1	HOXB5	3215	-2.1
IP6K2	51447	2.1	ZNF358	140467	-2.1
LINS1	55180	2.1	ANKRD36B	57730	-2.1
REV1	51455	2.1	P2RY6	5031	-2.0
SLC38A2	54407	2.1	PNPO	55163	-2.0
IKZF5	64376	2.1	ADCK2	90956	-2.0
CXCL2	2920	2.1	CASP6	839	-2.0
CREB5	9586	2.1	ECHDC2	55268	-2.0

CLK4	57396	2.1	C22orf36	388886	-2.0
CLK1	1195	2.1	MAB21L2	10586	-2.0
TNNC2	7125	2.1	RPP40	10799	-2.0
DCP1A	55802	2.1	MAP2K5	5607	-2.0
TBC1D15	64786	2.1	ABCA7	10347	-2.0
WDR47	22911	2.1	HMBS	3145	-2.0
RIOK3	8780	2.1	ARHGAP26	23092	-2.0
ABTB2	25841	2.1	RAD51L3	5892	-2.0
HTR1A	3350	2.1	CHST12	55501	-2.0
FGFR1OP	11116	2.1	METTL13	51603	-2.0
SMURF2	64750	2.1	EIF2B3	8891	-2.0
TNFRSF9	3604	2.1	MAPT	4137	-2.0
DDX10	1662	2.1	LIG1	3978	-2.0
CSGALNACT2	55454	2.1	IMPDH2	3615	-2.0
VNN2	8875	2.0	PDK2	5164	-2.0
WBP4	11193	2.0	PALB2	79728	-2.0
CTNNAL1	8727	2.0	C9orf125	84302	-2.0
DCP2	167227	2.0	MPP2	4355	-2.0
TDG	6996	2.0	B9D1	27077	-2.0
FSTL3	10272	2.0	MREG	55686	-2.0
YOD1	55432	2.0	ALX1	8092	-2.0
ATAD2B	54454	2.0	QDPR	5860	-2.0
CDCA4	55038	2.0	UROS	7390	-2.0
HIC2	23119	2.0	PTPRA	5786	-2.0
KIRREL	55243	2.0	JUP	3728	-2.0
BTN2A1	11120	2.0	POP5	51367	-2.0
PRPF38B	55119	2.0	ZNF688	146542	-2.0
MTSS1L	92154	2.0	AMACR	23600	-2.0
JHDM1D	80853	2.0	N6AMT1	29104	-2.0
STX1A	6804	2.0	RFX5	5993	-2.0
SEMA3C	10512	2.0	MUC5B	727897	-2.0

TBC1D22B	55633	2.0
THUMPD2	80745	2.0
SGPP1	81537	2.0
FBXO3	26273	2.0
CCNL2	81669	2.0
EGFR	1956	2.0
TNFAIP2	7127	2.0
COQ10B	80219	2.0
CCNH	902	2.0
SYNJ1	8867	2.0
GOLGA8A	23015	2.0
SMG5	23381	2.0
TIPARP	25976	2.0
FBXW7	55294	2.0
BCL10	8915	2.0
RAGE	5891	2.0
ZBTB11	27107	2.0
INTS6	26512	2.0
