# Supplementary material for: Helveticoside is a biologically active component of the seed extract of Descurainia sophia and induces reciprocal gene regulation in A549 human lung cancer cells
Source: BMC Genomics. 2015 Sep 18;16(1):713. doi: 10.1186/s12864-015-1918-1 (PMC4575430; doi:10.1186/s12864-015-1918-1)
Supplement: Additional file 2: — Full list of genes in each pattern after treatment with helveticoside. (PDF 370 kb) [file 12864_2015_1918_MOESM2_ESM.pdf]

Additional file 2. Full list of genes in each pattern after treatment with helveticoside.

Down-regulated pattern					Up-regulated pattern				
Symbol	GeneID	Helveticoside (nM)			Symbol	GeneID	Helveticoside (nM)		
		3.75	15	60			3.75	15	60
CLDN2	9075	-0.11	-2.71	-4.41	EGR1	1958	1.17	2.03	6.28
HSPA8	3312	-1.2	-3.27	-4.12	GADD45B	4616	0.55	1.21	4.31
CSRP2BP	57325	-1.26	-2.11	-3.9	SMOX	54498	2.94	3.47	4.14
MFSD3	113655	-0.08	-0.86	-3.86	CYR61	3491	0.79	1.7	4.1
NNMT	4837	0.01	-1.82	-3.66	IL8	3576	1.07	2.41	4.02
SCARA5	286133	0.08	-1.22	-3.62	IL11	3589	0.47	1.92	3.81
HLA-DMB	3109	0.1	-0.71	-3.59	PPP1R15A	23645	0.53	1.56	3.7
GPRIN2	9721	-1.78	-2.86	-3.52	MAFF	23764	0.8	1.96	3.67
HEXIM2	124790	-0.24	-1.38	-3.48	FOSB	2354	-0.04	0.61	3.51
TMEM37	140738	-0.07	-2.06	-3.47	C3ORF52	79669	0.46	1.23	3.42
GPX2	2877	-0.01	-2	-3.46	LY6K	54742	0.62	2.5	3.37
NTHL1	4913	-0.22	-1.38	-3.45	GADD45A	1647	-0.13	1.09	3.36
CNPY2	10330	-0.68	-1.51	-3.42	FOSL1	8061	0.58	1.61	3.36
BAAT	570	-0.86	-2.53	-3.39	EREG	2069	1.13	2.42	3.31
MORC4	79710	-0.71	-2.07	-3.32	BIRC3	330	1.14	2.29	3.29
B4GALNT1	2583	-0.67	-2.48	-3.31	LOC387763	387763	1.37	2.13	3.24
FOXS1	2307	0.79	-1.18	-3.18	EMP1	2012	0.81	2.39	3.19
HSPA1A	3303	-1.01	-2.55	-3.15	ADM	133	-0.2	0.85	3.13
SMO	6608	-0.39	-1.73	-3.14	GREM1	26585	0.5	1.47	3.13
HOXB13	10481	0.1	-1.22	-3.09	SERPINE1	5054	0.09	1.57	3.11
LXN	56925	-0.38	-1.75	-3.05	C1ORF133	574036	0.73	2	3.04
LOC285178	285178	-1.39	-2.5	-3.03	CD274	29126	0.71	1.65	3.04
HSPA2	3306	-0.29	-2.21	-3.01	DNAJB2	3300	0.01	1.56	3.03
C20ORF94	128710	-1	-1.71	-3	RASSF1	11186	0.21	1.57	2.98
NICN1	84276	-0.27	-1.38	-2.99	IRAK2	3656	1.09	1.81	2.98
C3ORF18	51161	-0.38	-1.89	-2.94	GEM	2669	1.33	1.63	2.96

SALL2	6297	-0.74	-1.95	-2.93	CTGF	1490	0.26	1.37	2.94
ACOX2	8309	0.21	-1.6	-2.86	UGCG	7357	0.77	1.26	2.89
RIMBP3	85376	-1.45	-1.73	-2.86	BBC3	27113	0.84	2.16	2.89
MARVELD1	83742	-0.26	-1.45	-2.85	GABARAPL1	23710	0.45	1.76	2.87
EEF2K	29904	-1.13	-2.06	-2.83	NOG	9241	0.01	1.37	2.87
TST	7263	-0.22	-1.38	-2.78	MAP3K14	9020	-0.28	1.41	2.83
MTMR4	9110	-0.68	-1.34	-2.77	RELB	5971	0.96	1.97	2.83
HPS6	79803	-0.46	-1.48	-2.72	DUSP1	1843	0.78	1.68	2.82
LOC645249	645249	-0.51	-1.57	-2.72	AREG	374	0.84	2	2.75
IDH1	3417	-0.11	-0.72	-2.7	SDC4	6385	0.5	1.62	2.72
NUDT18	79873	-0.48	-1.71	-2.7	PPP1R13L	10848	0.13	1.08	2.69
C12ORF27	283460	-0.37	-1.86	-2.69	AEN	64782	0.64	1.58	2.69
EPHX2	2053	0.03	-1.7	-2.68	VASN	114990	0.98	1.71	2.69
SNORA12	677800	-0.96	-2.32	-2.68	PER1	5187	1.72	1.33	2.68
BCAS1	8537	-0.08	-1.71	-2.67	SOCS3	9021	1.39	1.52	2.68
ST6GAL1	6480	-0.43	-1.63	-2.64	C5ORF41	153222	0.81	1.94	2.65
GABRB3	2562	-0.13	-1.45	-2.64	ARL5B	221079	0.17	0.7	2.63
PNMAL1	55228	-0.79	-1.73	-2.63	JUN	3725	0.69	1.47	2.59
SDSL	113675	-0.08	-1.01	-2.62	CSRNP1	64651	0.84	1.29	2.59
CDNF	441549	-2.03	-2.2	-2.61	NFKBIE	4794	-0.06	0.87	2.58
HSPA1B	3304	-0.84	-2.28	-2.59	GOLT1A	127845	0.18	1.64	2.58
LOC400099	400099	-0.56	-1.41	-2.59	NIPAL4	348938	0.24	1.62	2.57
CTDSPL	10217	-0.52	-1.13	-2.58	PAGE2	203569	0.72	0.83	2.57
FANCF	2188	-0.74	-1.1	-2.58	EPHA2	1969	0.74	1.51	2.56
HPGD	3248	-0.08	-1.3	-2.57	ZNF341	84905	0.92	1.8	2.56
DACT2	168002	-1.02	-2.13	-2.57	RHCG	51458	0.96	2.41	2.54
CBR3	874	-0.59	-1.79	-2.55	FOXD1	2297	0.3	1.06	2.53
CCDC106	29903	-0.15	-1.3	-2.55	TNFRSF10A	8797	0.45	1.63	2.52
TRIM16L	147166	-0.89	-1.3	-2.55	SERTAD1	29950	0.82	0.93	2.52
TCEA2	6919	-0.19	-1.34	-2.54	CLDN1	9076	0.21	1.45	2.5
C1QTNF6	114904	0.32	-0.9	-2.54	PIM1	5292	0.56	1.43	2.49

FBXO16	157574	-0.77	-1.64	-2.52	PIM3	415116	0.9	1.45	2.49
HEXDC	284004	-0.61	-1.37	-2.52	FST	10468	0.59	2.09	2.48
HCG_2003663	728061	-0.21	-1.38	-2.5	TXNIP	10628	0.58	1.78	2.46
C14ORF93	60686	0.06	-1.1	-2.44	EDN2	1907	0.37	1.21	2.44
FBXO9	26268	-1.17	-2	-2.43	PTHLH	5744	1.4	1.59	2.42
QARS	5859	-0.03	-0.79	-2.43	TNFRSF10D	8793	1.13	2.2	2.42
ABCA3	21	-0.25	-1.33	-2.4	TUBA8	51807	0.36	1.37	2.42
NCRNA00094	266655	-0.9	-2.18	-2.39	NUAK2	81788	0.21	0.47	2.41
C3ORF31	132001	0	-1.07	-2.39	TUBA3C	7278	0.32	1.32	2.4
THSD7A	221981	-0.21	-1.56	-2.38	LCAT	3931	0.13	0.98	2.39
C17ORF97	400566	-0.46	-1.84	-2.38	REM2	161253	0.37	1.34	2.38
B3GALT6	126792	-0.36	-0.85	-2.38	ATF3	467	0.48	1.45	2.37
SMARCAL1	50485	-0.68	-1.2	-2.36	KDM6B	23135	0.38	1.3	2.37
JMJD4	65094	-1.94	-1.97	-2.36	TUBA4A	7277	0.26	1.25	2.36
HNRNPA1L2	144983	-0.09	-1.05	-2.36	SLC38A2	54407	0.88	1.66	2.35
SYT17	51760	-0.53	-1.23	-2.35	TMEM156	80008	0.47	1.32	2.34
SELENBP1	8991	0.01	-1.72	-2.34	AMOTL2	51421	0.21	1.48	2.33
PGD	5226	-0.16	-1.17	-2.33	B3GNT5	84002	0.27	1.2	2.33
LARGE	9215	-0.09	-1.43	-2.33	LOC399959	399959	0.54	1.68	2.33
EPB41L4A	64097	-1	-1.73	-2.33	SLC19A2	10560	0.51	1.26	2.32
C6ORF176	90632	-0.36	-1.35	-2.33	METRNL	284207	-0.18	1.1	2.32
NDRG4	65009	-0.47	-2.15	-2.32	SERTAD4	56256	0.35	1.01	2.32
ZFP90	146198	-0.99	-1.52	-2.32	TNFRSF10B	8795	0.57	1.57	2.3
DPYSL5	56896	-0.46	-2	-2.32	BTG2	7832	0.19	0.76	2.29
SNORD116-19	727708	-0.58	-1.67	-2.31	PLK3	1263	1.2	1.74	2.28
MAGEH1	28986	-0.09	-0.96	-2.3	ROR1	4919	0.52	1.47	2.28
LOC100288418	100288418	-0.36	-1.29	-2.3	PDGFB	5155	0.08	0.93	2.26
VAV3	10451	-0.06	-1.66	-2.29	MAFK	7975	0.5	1.69	2.26
ANXA13	312	0.17	-0.76	-2.26	FRMD6	122786	0.15	0.79	2.26
KAT2A	2648	-0.2	-1.21	-2.25	SLC22A5	6584	0.29	1.42	2.25
HGD	3081	-0.21	-0.57	-2.25	SESN2	83667	0.81	1.97	2.24

RHOBTB1	9886	-0.66	-1.36	-2.25	ARID3A	1820	0.49	1.18	2.23
C5ORF58	133874	0.04	-0.93	-2.25	MAP2K3	5606	0.5	1.16	2.23
RBM45	129831	-0.66	-1.36	-2.25	YRDC	79693	0.59	0.95	2.22
APITD1	378708	-0.48	-1.37	-2.24	EFNA1	1942	0.61	1.05	2.21
AZI1	22994	-0.7	-1.15	-2.24	KLF10	7071	0.23	0.9	2.2
PTPRA	5786	-0.03	-0.95	-2.23	RBM24	221662	0.13	1.26	2.19
C17ORF108	201229	-0.37	-1.13	-2.22	JUNB	3726	0.73	1.18	2.17
CR848007.8	653436	-1.9	-1.8	-2.22	SLMO1	10650	0.09	1.37	2.17
AKR1B15	441282	0.03	-0.89	-2.21	MCTP1	79772	0.24	1.58	2.17
TMEM149	79713	0.09	-1.64	-2.2	FSTL3	10272	0.16	1.27	2.16
TM4SF20	79853	0.22	-1.82	-2.2	TSPAN5	10098	0.24	0.99	2.15
RPUSD1	113000	-0.64	-1.33	-2.2	NR1D1	9572	0.64	0.66	2.15
THYN1	29087	-0.05	-1.2	-2.19	PEA15	8682	0.12	1.06	2.14
NT5M	56953	0.1	-0.5	-2.19	LRRC8A	56262	0.36	1.2	2.14
C11ORF71	54494	0.46	-0.87	-2.18	PRR24	255783	0.55	1.83	2.14
GALM	130589	-0.01	-1.26	-2.18	CDC42EP1	11135	0.39	1.35	2.12
B3GALT4	8705	-0.01	-1.28	-2.17	ATAD1	84896	0.36	1.38	2.12
CBR4	84869	-0.08	-0.79	-2.17	BTG1	694	0.41	1.07	2.11
ANXA4	307	-0.18	-1.33	-2.16	GPRC5B	51704	0.02	0.39	2.11
CCR7	1236	-0.11	-0.58	-2.16	GATA6	2627	0.8	0.95	2.11
SPDEF	25803	0.16	-1.74	-2.16	KLF4	9314	0.57	0.99	2.11
C6ORF153	88745	-0.88	-1.63	-2.15	NCRNA00152	112597	0.22	1.2	2.1
PTDSS1	9791	-0.17	-1.22	-2.14	TNFAIP2	7127	0.5	1.34	2.08
GEMIN6	79833	-0.38	-1.15	-2.14	H1F0	3005	0.33	1.54	2.08
ZNF18	7566	-0.36	-1.13	-2.14	NFKBIB	4793	0.2	1.04	2.08
SPSB2	84727	-0.35	-0.94	-2.14	IL1RAP	3556	0.57	1.46	2.07
STAT6	6778	-0.7	-1.25	-2.13	YIPF4	84272	0.56	1.62	2.07
DIXDC1	85458	-0.32	-0.93	-2.13	STX3	6809	0.11	0.95	2.06
SLFN11	91607	-0.75	-1.95	-2.13	ARID3B	10620	0.23	0.76	2.06
NIPSNAP3A	25934	0.14	-1.31	-2.13	ITPRIP	85450	0.74	1.36	2.06
KRCC1	51315	-0.97	-1.47	-2.12	LATS2	26524	0.43	1.22	2.06

PCYOX1L	78991	0.01	-1.44	-2.12	EHD1	10938	0.23	1.19	2.05
MEX3A	92312	-0.93	-1.68	-2.12	IER5	51278	0.92	1	2.04
NMRAL1	57407	-0.31	-1.15	-2.12	PDRG1	81572	-0.39	0.65	2.04
ZNF575	284346	-0.4	-1.3	-2.12	CRY1	1407	-0.3	0.82	2.01
CYP2R1	120227	-0.04	-0.57	-2.1	ZNF335	63925	0.76	1.37	2.01
C16ORF42	115939	-0.11	-1.04	-2.1	UPF1	5976	-0.43	0.67	2
ZNF362	149076	-0.43	-1.21	-2.1	PLD6	201164	-0.23	0.9	2
CAT	847	0	-1.45	-2.09	IER2	9592	0.9	1.06	1.99
CD14	929	0.39	-1.21	-2.09	CCDC29	728788	0.12	1.28	1.99
HDDC3	374659	0	-0.81	-2.09	STK40	83931	0.59	1.58	1.98
TRADD	8717	-0.38	-1.14	-2.08	C1ORF77	26097	0.27	1.38	1.97
CD24	100133941	-0.25	-2	-2.08	NAB2	4665	0.4	0.4	1.97
PDE8B	8622	-0.75	-1.45	-2.08	ODC1	4953	0.18	1.18	1.96
NAMPT	10135	-0.18	-1.9	-2.08	AOC3	8639	0.2	1.12	1.95
C11ORF35	256329	-0.28	-1.59	-2.08	NMS	129521	-0.33	-0.04	1.95
RECQL4	9401	-0.6	-1.75	-2.06	LOC285441	285441	-0.19	-0.1	1.94
LOC100134259	100134259	0.05	-1.02	-2.06	TNPO2	30000	0.34	1.09	1.93
SCARNA9	619383	-0.71	-1.18	-2.06	PRDM1	639	0.63	0.99	1.93
SNORA77	677843	-2.02	-1.73	-2.06	CDC42SE1	56882	0.41	1.1	1.93
RDBP	7936	-0.02	-1.03	-2.05	ASB10	136371	0.49	1.64	1.93
RAB15	376267	-0.41	-1.75	-2.05	NCEH1	57552	0.55	1.16	1.93
AKR1B1	231	0.03	-0.95	-2.04	CTSK	1513	0.63	1.37	1.92
CAPN5	726	0.09	-0.46	-2.04	VEGFA	7422	0.48	1.57	1.92
RGS10	6001	0.03	-1.04	-2.03	YOD1	55432	0.63	1.47	1.92
PRIM1	5557	-0.9	-1.53	-2.03	TUFT1	7286	-0.2	0.86	1.91
PIR	8544	-0.07	-1.05	-2.03	ABL2	27	0.07	0.57	1.91
SCARNA13	677768	-0.38	-0.87	-2.03	PI4K2A	55361	0.28	0.9	1.91
PIK3R2	5296	-0.15	-0.8	-2.02	MXD1	4084	0.54	1.13	1.9
LRRC20	55222	-0.43	-0.82	-2.02	KRT10	3858	0.28	1.13	1.9
DPYSL3	1809	-0.03	-1.39	-2	TBX2	6909	0.63	0.99	1.89
OSBPL7	114881	-0.42	-1.02	-2	MARCH4	57574	0.19	1.16	1.89

MAGED4B	81557	-0.35	-1.44	-2	UBE2S	27338	0.44	1.13	1.88
UROS	7390	-0.29	-1.21	-1.99	SLC35F2	54733	0.3	1.12	1.88
F12	2161	-0.08	-1.07	-1.99	CLCF1	23529	0.43	1.04	1.88
C1ORF93	127281	-0.88	-1.05	-1.99	CCNH	902	0.18	0.53	1.87
AKR1B10	57016	0.04	-0.8	-1.97	DUSP6	1848	0.81	1.02	1.87
SYT12	91683	-0.29	-1.15	-1.97	ZNF143	7702	0.34	0.93	1.87
SLC40A1	30061	-0.05	-1	-1.97	AMD1	262	0.21	1.31	1.86
SNAPIN	23557	-0.35	-1.08	-1.97	MAP1LC3B	81631	0.39	1.25	1.86
MYLK	4638	-0.27	-1.5	-1.96	YPEL5	51646	0.5	1.09	1.86
LRFN3	79414	-0.07	-0.66	-1.96	LOC100131209	100131209	1.38	1.54	1.86
ANXA6	309	-0.26	-1.6	-1.95	DDX3Y	8653	0.36	0.9	1.85
SETBP1	26040	-0.36	-1.42	-1.95	NFAT5	10725	0.14	0.92	1.85
B9D1	27077	-0.17	-1.28	-1.94	PFKFB4	5210	0.53	1.14	1.84
MUTYH	4595	-0.42	-1.07	-1.94	AVPI1	60370	0.44	0.92	1.84
CCDC53	51019	-0.12	-1.25	-1.94	ADCY10	55811	0.2	0.47	1.83
FAM175A	84142	-0.34	-1.28	-1.94	PHC2	1912	0.6	1.07	1.82
TTC30B	150737	-0.64	-1.16	-1.94	ELFN2	114794	0.41	0.79	1.82
LIG1	3978	-0.09	-1.27	-1.93	ORAOV1	220064	-0.36	0.53	1.82
MTUS1	57509	-0.42	-1.11	-1.93	ID2	3398	0.59	0.98	1.81
C16ORF59	80178	-0.48	-0.91	-1.93	TNFAIP3	7128	0.14	0.42	1.81
BTBD6	90135	-0.82	-1.37	-1.93	SEMA3C	10512	0.39	1.01	1.81
C20ORF108	116151	-0.3	-1.41	-1.93	TLE4	7091	0.26	0.77	1.81
LOC729680	729680	-0.98	-1.5	-1.93	DCP1A	55802	0.41	1	1.81
RHOV	171177	0.29	-0.9	-1.93	ARHGAP15	55843	0.36	0.28	1.8
ZNF793	390927	-1.99	-1.72	-1.93	CHIC2	26511	0.43	0.82	1.8
OAZ1	4946	-0.06	-1.06	-1.92	LOC652191	652191	0.62	1.29	1.8
PYCARD	29108	0.13	-0.55	-1.92	ANKRD1	27063	0.52	1.01	1.79
NDUFB10	4716	-0.02	-0.55	-1.91	CDCA4	55038	0.75	0.69	1.79
NOTCH3	4854	0.39	-0.92	-1.9	PRKCE	5581	0.59	1.43	1.78
PLD2	5338	-0.07	-1.03	-1.9	PKP2	5318	-0.18	0.3	1.78
DET1	55070	-1.04	-1.05	-1.9	PDE12	201626	0.37	0.62	1.78

SFRS13B	135295	-1.46	-1.67	-1.9	C7ORF53	286006	0.81	1.38	1.78
PRKCDBP	112464	-0.04	-0.83	-1.89	LOC392288	392288	0.52	1.27	1.78
NUDT7	283927	0.1	-0.81	-1.89	TERF2IP	54386	0.81	1.08	1.77
EXD2	55218	-1.03	-1.43	-1.89	COL4A1	1282	0.33	0.98	1.77
LOC375190	375190	-0.42	-1.21	-1.89	GDF6	392255	0.01	0.37	1.77
TSTA3	7264	-0.18	-1.6	-1.88	MCL1	4170	0.94	1.32	1.76
DOK1	1796	-0.31	-0.95	-1.88	TAP1	6890	0.56	1.57	1.76
HCG_1776007	146713	0.42	0.08	-1.88	NR4A3	8013	1.21	1.08	1.76
TBCD	6904	-0.1	-1.24	-1.87	VPS37B	79720	0.14	0.72	1.76
RPP40	10799	-0.26	-1.16	-1.87	MGC12916	84815	0.76	0.48	1.76
SIDT1	54847	0.31	-0.99	-1.87	CCDC64B	146439	-0.01	0.43	1.76
ZC4H2	55906	-0.25	-1.3	-1.87	EXOC3L2	90332	-0.17	0.32	1.76
PHKB	5257	-0.24	-0.97	-1.86	ADRB1	153	0.34	1.14	1.75
NETO2	81831	-0.04	-0.96	-1.86	LYPD1	116372	0.24	1.18	1.75
TNS4	84951	0.39	-0.35	-1.86	TXNL4B	54957	-0.34	0.33	1.75
CXXC5	51523	0.02	-0.97	-1.86	TOP1P2	7152	0.42	0.9	1.75
ANKRD16	54522	-0.72	-0.9	-1.86	PRKAB1	5564	0.19	0.72	1.74
LIPC	3990	-1.2	-1.33	-1.85	NPC1	4864	0.2	1.04	1.74
ELAC1	55520	-1.13	-1.46	-1.85	RIT1	6016	-0.01	0.92	1.74
MPI	4351	-0.19	-0.96	-1.84	VGF	7425	0.51	1.08	1.73
BDKRB2	624	0.62	-0.14	-1.84	ELK3	2004	0.42	0.94	1.73
CUL9	23113	-0.1	-0.51	-1.84	HAS2	3037	0.4	0.57	1.73
LOC283482	283482	-1.17	-1.55	-1.84	PHLDA2	7262	0.93	0.88	1.73
ALDH3A1	218	0.19	-1.03	-1.83	TICAM1	148022	0.6	1.1	1.73
MAP2K6	5608	-0.57	-1.76	-1.83	RSC1A1	6248	0.19	0.37	1.73
KRT4	3851	-0.06	-1.25	-1.83	HOXC13	3229	0.5	1.09	1.73
SEPX1	51734	-0.27	-1.31	-1.83	C19ORF61	56006	0.07	1.01	1.73
NTNG2	84628	-0.19	-1.3	-1.83	DAGLB	221955	0.38	0.95	1.73
LOC554202	554202	-0.04	-1.19	-1.83	C3ORF59	151963	0.16	0.36	1.73
KRT19	3880	0.08	-1.05	-1.82	IER3	8870	0.88	1.18	1.72
NTRK3	4916	-0.15	-1.7	-1.82	EIF1B	10289	0.85	1.46	1.72

CYB5A	1528	0.1	-1.05	-1.82	PMAIP1	5366	0.66	1.3	1.72
MYH10	4628	-0.17	-1.05	-1.82	PLEK2	26499	0.14	0.99	1.72
CCDC80	151887	-0.21	-1.11	-1.82	C1GALT1	56913	0.42	0.92	1.72
DDIT4L	115265	-0.72	-1.08	-1.82	CHST11	50515	0.41	1.44	1.71
CDH1	999	0.17	-1.13	-1.81	PHLDB3	653583	-0.23	0.66	1.71
MPST	4357	0.01	-0.5	-1.81	DDX10	1662	0.5	1.18	1.7
CGNL1	84952	-0.31	-1.71	-1.81	DUSP5	1847	0.97	1.67	1.7
RAB37	326624	-0.12	-1.06	-1.8	UPF3B	65109	0.15	0.97	1.7
UBAC1	10422	-0.33	-0.88	-1.79	KCTD11	147040	-0.12	0.39	1.7
MVK	4598	-0.26	-1.36	-1.79	KCNJ14	3770	0.73	1.21	1.69
LOC375295	375295	-0.35	-1.21	-1.79	LOC286109	286109	0.26	0.76	1.69
BTBD11	121551	0.11	-0.03	-1.79	GSTTP2	653399	-0.16	0.24	1.69
HNRNPA1	3178	0.13	-0.93	-1.78	TGM2	7052	0.27	0.76	1.68
MTIF2	4528	-0.4	-0.51	-1.78	TFAP2C	7022	0.56	1.27	1.68
PIK3C2B	5287	-0.32	-0.49	-1.78	PPP1R2P9	80316	0.32	0.08	1.68
OCEL1	79629	0.49	-0.53	-1.78	CLDND1	56650	0.33	0.99	1.68
LOC644189	644189	-0.59	-1.29	-1.78	TM4SF1	4071	0.11	0.73	1.67
RHPN1	114822	-0.61	-1.08	-1.78	EAF1	85403	0.31	0.75	1.67
EIF2B3	8891	-0.16	-0.69	-1.77	FAM72A	729533	0.74	1.24	1.67
LOC439949	439949	-0.07	-0.93	-1.77	LOC388965	388965	0.28	1.11	1.67
ZBTB42	100128927	-0.39	-1.17	-1.77	FBXL21	26223	0.46	0.66	1.66
OAS1	4938	0.16	-0.84	-1.76	CDK6	1021	-0.36	0.39	1.65
SQRDL	58472	-0.15	-0.82	-1.76	ID4	3400	1.11	0.7	1.65
USP9X	8239	-0.53	-1.4	-1.75	MAPK8IP3	23162	0.12	0.81	1.65
IMPA2	3613	-0.25	-0.8	-1.75	KLHL28	54813	0.61	0.95	1.65
PBX1	5087	-0.29	-0.78	-1.75	MYO1G	64005	0.82	0.82	1.65
RHOBTB2	23221	-0.05	-0.41	-1.75	CTNNAL1	8727	0.18	1.06	1.64
WDR4	10785	-0.97	-1.01	-1.75	MITF	4286	0.39	1.18	1.64
LOC100132733	100132733	-0.62	-1.26	-1.75	TIPARP	25976	0.3	1	1.64
SLC25A29	123096	-0.36	-1.6	-1.75	LOC157562	157562	0.38	1.17	1.64
CCNB3	85417	-1.23	-1.41	-1.75	PPM1D	8493	0.37	0.86	1.63

SAC3D1	29901	-0.42	-0.9	-1.74	REL	5966	0.42	0.8	1.63
SNRNP25	79622	-0.12	-1.41	-1.74	AKAP12	9590	-0.28	0.69	1.63
ECHDC2	55268	-0.4	-1.42	-1.74	ORAI1	84876	0.44	0.82	1.63
C4ORF42	92070	-0.71	-1.2	-1.74	JAZF1	221895	0.42	0.74	1.63
ADSSL1	122622	0.27	-0.74	-1.74	TNFSF9	8744	1.12	1.21	1.62
POLR3GL	84265	0.16	-0.65	-1.74	KCNF1	3754	0.37	1.11	1.62
BFSP1	631	0.13	-0.47	-1.73	CRY2	1408	0.19	-0.03	1.62
LOC729983	729983	-0.34	-0.34	-1.73	IGF2BP2	10644	0.12	0.84	1.62
PDCD2L	84306	-0.41	-0.8	-1.73	CITED4	163732	0.24	1.16	1.62
OMA1	115209	-0.1	-1.28	-1.73	BTBD10	84280	0.13	0.57	1.62
RIMKLA	284716	-0.15	-1.49	-1.73	DDX6	1656	0.22	1.12	1.61
FAM102A	399665	0.09	-0.37	-1.72	PDP1	54704	0.46	1.14	1.61
SPATA7	55812	-0.32	-1.01	-1.72	PLOD2	5352	0.08	0.83	1.6
XRCC6BP1	91419	-0.23	-1.09	-1.72	FGFR1OP	11116	0.07	0.67	1.6
MDP1	145553	0.18	-0.66	-1.72	CORO1C	23603	0.08	0.47	1.6
ROS1	6098	-1.47	-1.11	-1.71	FNIP1	96459	0.14	0.73	1.6
DOK4	55715	0.06	-0.55	-1.71	HIVEP1	3096	-0.05	0.44	1.59
TPCN1	53373	-0.29	-1.05	-1.71	TOP1	7150	0.38	0.82	1.59
SLC25A10	1468	-0.33	-1.35	-1.71	DMPK	1760	0	0.7	1.59
KLHDC9	126823	-0.05	-1.24	-1.71	FAM83G	644815	0.55	0.93	1.59
STARD7	56910	-1	-1.48	-1.7	LETM2	137994	0.12	1.03	1.59
HS6ST1	9394	0.05	-1.02	-1.7	LRRC8C	84230	0.11	0.64	1.59
DHRS1	115817	0.03	-1.02	-1.7	BCAR3	8412	0.61	1.28	1.58
FAM162A	26355	0.02	-0.96	-1.7	ZBTB11	27107	0.43	0.75	1.58
LIPT2	387787	-0.04	-0.79	-1.7	RANGAP1	5905	-0.13	0.47	1.58
SLC9A3R1	9368	-0.01	-1.45	-1.69	WEE1	7465	0.75	0.52	1.58
BCKDK	10295	-0.12	-0.93	-1.69	LRRC8E	80131	-0.03	0.35	1.58
EHMT2	10919	-0.4	-0.79	-1.69	FLJ46120	647008	0.08	0.29	1.58
KIAA0101	9768	0.03	-0.93	-1.69	GABPB1	2553	-0.22	0.45	1.57
FCGBP	8857	0.13	-1.13	-1.69	TAF13	6884	0.27	0.77	1.57
P2RY6	5031	-0.13	-1.01	-1.69	DR1	1810	0.56	1.15	1.57

CBR1	873	-0.12	-0.71	-1.69	ZFR2	23217	-0.17	-0.12	1.57
DLL3	10683	0.22	-0.6	-1.69	LOC285697	285697	0.21	0.29	1.57
RNF213	57674	-0.41	-1.45	-1.69	CD55	1604	0.62	1.36	1.56
A2LD1	87769	-0.17	-1.4	-1.69	THAP1	55145	0.41	0.87	1.56
KIAA0100	9703	-0.35	-0.86	-1.68	CORIN	10699	-0.06	-0.03	1.56
TREX1	11277	-0.1	-0.85	-1.68	ZSWIM4	65249	0.45	0.88	1.56
ZNF33B	7582	-0.88	-1.03	-1.68	RELT	84957	0.39	0.44	1.56
RSAD1	55316	-0.27	-0.83	-1.68	TRAM2	9697	-0.29	0.59	1.55
LYPLAL1	127018	-0.11	-0.93	-1.68	CSNK1D	1453	0.07	0.9	1.55
LOC440047	440047	-1.45	-1.17	-1.68	PPAP2B	8613	0.5	0.66	1.55
ACADSB	36	-0.31	-1.05	-1.67	MORC3	23515	0.42	0.73	1.55
GALNT10	55568	-0.39	-1.58	-1.67	SAR1B	51128	0.21	0.9	1.55
SGCD	6444	0.01	-1.07	-1.67	PAIP2	51247	0.32	1	1.55
PNPO	55163	-0.19	-1.04	-1.67	TRIML2	205860	0.16	0.93	1.55
UBE2D4	51619	-0.17	-0.88	-1.67	ZNF805	390980	0.4	1.03	1.55
NSMCE1	197370	-0.07	-0.93	-1.67	CBY3	646019	0.3	0.59	1.55
HCG_1659830	728111	0.14	-0.2	-1.67	BAK1	578	0.52	1.13	1.54
LOC100130111	100130111	0	-0.54	-1.67	DNMBP	23268	0.36	0.96	1.54
BAMBI	25805	0.05	-1.16	-1.66	GPN2	54707	0.04	0.36	1.54
FUT8	2530	0.08	-0.56	-1.66	CCL14	6358	-0.14	0	1.53
SMARCD3	6604	-0.26	-1.15	-1.66	HIC2	23119	0.34	0.77	1.53
QDPR	5860	0.26	-0.53	-1.66	UBE2W	55284	0.36	0.92	1.53
SLC9A3R2	9351	-0.13	-1.29	-1.66	LOC100133180	100133180	0.32	0.71	1.53
GMPPA	29926	-0.3	-0.52	-1.66	TGIF2LY	90655	0.28	0.58	1.53
C7ORF68	29923	-0.61	-0.84	-1.66	FAM126B	285172	-0.06	0.44	1.53
C16ORF74	404550	-0.44	-1.11	-1.66	SLC25A17	10478	0.25	0.78	1.52
C8ORF40	114926	-0.02	-0.67	-1.66	LAMC1	3915	0.24	1.05	1.51
OSTBETA	123264	0.29	-0.95	-1.66	BHLHE40	8553	1.31	1	1.51
SNORA62	6044	-1.03	-1.47	-1.66	PEX13	5194	0.24	0.75	1.51
IMPDH2	3615	0.08	-0.45	-1.65	GTPBP1	9567	0.56	0.63	1.51
EBAG9	9166	-0.37	-0.64	-1.65	FOSL2	2355	0.59	0.47	1.51

SEPT6	23157	-0.07	-0.82	-1.65	SH2D3A	10045	0.38	1.02	1.51
PDE7B	27115	-1.38	-1.43	-1.65	LEMD2	221496	0.08	0.68	1.51
DCDC2	51473	-0.48	-1.48	-1.65	LOC93444	93444	0.14	0.35	1.51
FLJ90757	440465	-0.48	-1.31	-1.65	CHMP4C	92421	-0.06	0.61	1.51
C16ORF35	8131	-0.6	-0.94	-1.64	PCMT1	5110	0.2	0.6	1.5
GNAZ	2781	0.14	-0.45	-1.64	ZNF609	23060	0.34	0.99	1.5
ARHGAP26	23092	-0.04	-0.82	-1.64	TMEM194A	23306	0.04	0.67	1.5
RBBP9	10741	-0.33	-1.02	-1.64	C6ORF145	221749	0.14	0.89	1.5
WDR54	84058	-0.27	-1.26	-1.64	COTL1	23406	0.13	0.98	1.5
CYB5B	80777	-0.29	-0.89	-1.63	SLC7A6OS	84138	0.32	1.02	1.5
GRB14	2888	-0.13	-0.66	-1.63	SLC25A25	114789	0.47	0.64	1.5
ZNF643	65243	-0.36	-1.03	-1.63	BTN2A1	11120	0.13	0.72	1.49
C6ORF62	81688	-0.85	-1.57	-1.63	RASGRP1	10125	-0.29	0.11	1.49
ZNF658	26149	-0.66	-0.84	-1.63	SERPINB8	5271	-0.02	0.71	1.49
PTGFRN	5738	0.22	-0.98	-1.63	TAF1A	9015	0.32	0.79	1.49
LOC286161	286161	-0.9	-1.32	-1.63	ARRDC4	91947	0.24	0.81	1.49
HS3ST1	9957	0.52	-0.47	-1.62	KIAA0182	23199	0.58	0.84	1.48
ALDH3B1	221	-0.29	-0.47	-1.62	RRP12	23223	-0.22	0.63	1.48
RHOQ	23433	-0.22	-0.65	-1.62	TRIAP1	51499	0.25	0.95	1.48
KIAA0528	9847	-0.36	-1.03	-1.62	COQ10B	80219	0.5	0.9	1.48
FCHO1	23149	0.12	-0.73	-1.62	PKD1L1	168507	0.29	0.49	1.48
EML4	27436	-0.54	-1.47	-1.62	DAPK3	1613	-0.46	0.34	1.47
LOC283352	283352	0.15	-0.35	-1.62	RRAS2	22800	0.43	0.72	1.47
HNRPDL	9987	-0.12	-1.6	-1.61	SMURF1	57154	0.36	0.82	1.47
POP5	51367	-0.09	-0.69	-1.61	RND3	390	0.56	1.07	1.47
MAGED1	9500	0.17	-1.13	-1.61	PHLDA1	22822	0.84	1.14	1.47
AKR1C3	8644	-0.06	-0.67	-1.61	SGPP1	81537	0.5	0.84	1.47
FHOD3	80206	-0.52	-1.12	-1.61	SBDS	51119	0.17	0.79	1.47
ARHGEF10L	55160	-0.23	-1.07	-1.61	TMEM196	256130	-0.12	-0.02	1.47
C10ORF114	399726	-0.22	-0.65	-1.61	ITGA5	3678	0.47	1.09	1.46
C3ORF1	51300	-0.19	-0.62	-1.61	INPP1	3628	0.52	1.13	1.46

DPYSL2	1808	0.21	-0.03	-1.6	CLK4	57396	1	1.03	1.46
CLDN3	1365	-0.12	-1.24	-1.6	RUSC2	9853	0.18	0.84	1.46
KCNJ6	3763	-0.23	-0.55	-1.6	ARL8B	55207	-0.02	0.62	1.46
TP53I3	9540	-0.31	-1.16	-1.6	REC8	9985	0.36	1.09	1.46
TTC12	54970	-0.5	-1.56	-1.6	CCBE1	147372	0.4	0.83	1.46
MRPS34	65993	0.08	-0.56	-1.6	CXCL1	2919	0.36	0.36	1.45
AMZ2	51321	-0.12	-0.72	-1.6	STC1	6781	0.87	0.86	1.45
LRRC8D	55144	-1.01	-1.24	-1.6	PFDN2	5202	0.31	0.91	1.45
POPDC3	64208	0.26	-0.98	-1.6	ZNF654	55279	0.3	0.51	1.45
FBXO4	26272	-0.3	-1.07	-1.6	LIN52	91750	0.36	0.76	1.45
DENND2D	79961	-0.47	-1.14	-1.6	MGC16703	113691	0.2	0.65	1.45
CCDC125	202243	-1.03	-1.17	-1.6	TMEM88	92162	0.06	0.74	1.45
LOC401431	401431	-1.3	-1.48	-1.6	IL32	9235	0.09	0.79	1.44
DBN1	1627	-0.53	-1.37	-1.59	LIF	3976	0.27	0.93	1.44
STK16	8576	0.07	-0.4	-1.59	SERINC1	57515	0.05	0.86	1.44
ADAT1	23536	-0.75	-1.05	-1.59	MICA	4276	0.46	1.1	1.44
TMEM143	55260	0.14	-0.91	-1.59	HCN3	57657	0.28	0.57	1.44
RNASEL	6041	-0.55	-0.83	-1.59	ATP1B1	481	0.5	0.89	1.43
GLRX	2745	-0.01	-0.57	-1.58	MRPL49	740	0.16	0.76	1.43
ZMYND8	23613	-0.43	-0.8	-1.58	STX1A	6804	0.24	0.9	1.43
HOXA6	3203	-0.61	-0.65	-1.58	DPH3B	100132911	0.18	0.82	1.43
WFDC8	90199	-1.39	-1.17	-1.58	LOC221710	221710	-0.08	0.45	1.43
PRMT7	54496	-0.66	-1.09	-1.58	ATXN2	6311	0.13	0.66	1.42
OSGEPL1	64172	-0.29	-0.33	-1.58	HMGCS1	3157	0.09	-0.01	1.42
THAP8	199745	-0.22	-0.91	-1.58	PLAUR	5329	0.58	0.98	1.42
C20ORF141	128653	0.16	-0.24	-1.58	PRKAB2	5565	-0.35	0.51	1.42
LOC388242	388242	0.18	-1.07	-1.58	TMEM167B	56900	0.3	0.97	1.42
LPGAT1	9926	-0.13	-1.09	-1.57	FGD4	121512	-0.26	0.42	1.42
HOXB6	3216	0.27	-0.87	-1.57	GDI1	2664	0.37	1.07	1.41
PACS2	23241	-0.35	-0.73	-1.57	NEU1	4758	0.31	0.79	1.41
MEGF8	1954	-0.19	-0.73	-1.57	FERMT2	10979	0.02	0.32	1.41

CHST12	55501	0.15	-0.9	-1.57	AKAP8L	26993	0.54	0.98	1.41
ACCS	84680	-0.21	-0.77	-1.57	UFM1	51569	0.2	0.91	1.41
MRPS26	64949	-0.03	-0.64	-1.57	RHBDF1	64285	-0.22	0.66	1.41
C9ORF23	138716	-0.12	-0.6	-1.57	BCOR	54880	0.6	0.88	1.41
C9ORF150	286343	0.42	-0.27	-1.57	MED15	51586	0.3	0.87	1.41
PACSIN1	29993	-0.24	-1.28	-1.57	ATG4D	84971	0.47	1	1.41
THRA	7067	-0.13	-1.17	-1.56	PPP1R15B	84919	0.11	0.79	1.41
SNAP25	6616	-0.1	-1.27	-1.56	SMAD7	4092	0.37	0.77	1.4
HEXIM1	10614	-0.05	-0.44	-1.56	ARID1A	8289	-0.04	0.5	1.4
LQK1	642946	-0.14	-0.91	-1.56	KLF6	1316	0	0.12	1.4
RNF135	84282	-0.24	-0.54	-1.56	GPATCH8	23131	0.23	0.86	1.4
PTPN18	26469	-0.08	-0.83	-1.55	MOSPD1	56180	0.32	0.81	1.4
CA12	771	0.13	-0.6	-1.55	SOCS2	8835	-0.2	0.16	1.39
TOP2B	7155	-0.2	-0.79	-1.55	RAB32	10981	0.29	0.86	1.39
DCTPP1	79077	-0.37	-1.1	-1.55	PHLDA3	23612	0.1	0.77	1.39
TMBIM4	51643	0.01	-1.16	-1.55	KLK12	43849	0.05	0.14	1.39
FLJ37798	401264	-0.21	-1.13	-1.55	OR5M1	390168	0.06	0.33	1.39
LOC93622	93622	-0.18	-0.92	-1.55	SAMD8	142891	0.22	0.57	1.39
SNORD123	100113384	-1.27	-0.94	-1.55	ARF4	378	0.1	0.44	1.38
CPOX	1371	0.22	-0.57	-1.54	HSPA13	6782	0.38	1.13	1.38
RGS19	10287	0	-0.75	-1.54	SERTAD2	9792	0.48	0.4	1.38
VDAC3	7419	-0.23	-0.93	-1.54	SP2	6668	0.2	0.61	1.38
LRP4	4038	0.27	-0.97	-1.54	OXTR	5021	-0.03	0.21	1.38
C8ORF55	51337	-0.03	-0.37	-1.54	CXCL2	2920	0.42	0.33	1.38
ALKBH2	121642	-0.36	-0.5	-1.54	RCL1	10171	-0.08	0.38	1.38
RILPL2	196383	-0.23	-0.54	-1.54	CDKN2AIP	55602	0.43	0.61	1.38
FABP5	2171	-0.21	-1.1	-1.53	CPEB2	132864	0.35	0.7	1.38
NEURL	9148	0.11	-0.25	-1.53	NIPA1	123606	0.45	0.84	1.38
HOXC4	3221	0.12	-0.17	-1.53	PLCXD2	257068	0.29	0.85	1.38
CASP6	839	-0.73	-0.87	-1.53	TMEM189	387521	0.4	0.97	1.38
C22ORF36	388886	-0.08	-0.68	-1.53	ANXA5	308	0.23	0.38	1.37

SLC24A6	80024	-0.02	-0.59	-1.53	NPTX1	4884	0.1	0.65	1.37
RAB26	25837	-0.22	-1.25	-1.53	CDC25A	993	0.12	0.16	1.37
ANAPC4	29945	-0.68	-1.17	-1.53	SLC35D1	23169	0.47	1.19	1.37
C6ORF203	51250	-0.55	-0.78	-1.53	GALNT2	2590	0.38	0.76	1.37
CD99L2	83692	-0.22	-1.08	-1.53	RBM22	55696	0.3	0.62	1.37
LOC100293090	100293090	-0.16	-0.74	-1.53	CSGALNACT2	55454	0.26	0.87	1.37
MIB1	57534	-0.1	-0.54	-1.53	LOC727847	727847	0.54	0.59	1.37
PELI3	246330	-1.19	-1.05	-1.53	CKS2	1164	0.42	0.32	1.36
NIPSNAP1	8508	0.13	-0.63	-1.52	DOCK4	9732	-0.04	0.67	1.36
TMEM80	283232	0.12	-1.13	-1.52	PPP3CC	5533	0.46	0.75	1.36
C20ORF96	140680	-0.53	-0.94	-1.52	MTSS1L	92154	0.02	0.57	1.36
LOC100131564	100131564	-0.36	-0.27	-1.52	FAM184A	79632	0.42	0.25	1.36
LOC158863	158863	-0.71	-0.63	-1.52	ANGPTL4	51129	1.04	1.35	1.36
GSTA4	2941	-0.01	-0.95	-1.51	C7ORF20	51608	-0.3	0.48	1.36
CDK5	1020	0.21	-0.64	-1.51	TDG	6996	0.22	0.54	1.35
ASB9	140462	0.18	-1.1	-1.51	LIMA1	51474	0.03	0	1.35
NSMCE4A	54780	-0.11	-0.99	-1.51	SLC25A37	51312	0.48	1.02	1.35
MOCS2	4338	-0.42	-0.76	-1.51	TMEM22	80723	0.28	0.89	1.35
ZDHHC4	55146	-0.25	-0.51	-1.51	CCNL1	57018	0.22	0.45	1.35
ZCWPW1	55063	-0.16	-0.9	-1.51	SBDSP	155370	0.31	0.89	1.35
TMEM117	84216	-0.4	-0.97	-1.51	DDI2	84301	0.1	0.49	1.35
LOC100133923	100133923	-0.71	-0.82	-1.51	LOC100131254	100131254	-0.05	-0.01	1.35
FANCG	2189	-0.15	-0.97	-1.5	THEM5	284486	-0.09	-0.04	1.35
MTMR15	22909	-0.26	-0.67	-1.5	FOXF2	2295	0.03	0.6	1.34
PPOX	5498	-0.02	-0.35	-1.5	PLA2G4C	8605	0.12	0.84	1.34
LHX2	9355	-0.2	-1.38	-1.5	MTO1	25821	0.31	0.85	1.34
PHKA2	5256	-0.08	-0.92	-1.5	SSH1	54434	0.13	0.52	1.34
C14ORF133	63894	-0.69	-1.1	-1.5	PEAR1	375033	0.2	0.82	1.34
NOL12	79159	-0.92	-1.39	-1.5	APCDD1L	164284	0.45	0.39	1.34
C17ORF90	339229	-0.56	-0.59	-1.5	PSMC3IP	29893	-0.16	0.66	1.33
ANKS6	203286	-0.51	-0.54	-1.5	SKIL	6498	0.78	0.88	1.33

PPIL3	53938	0.23	-0.86	-1.5	ELOVL1	64834	0.36	0.86	1.33
HSD17B4	3295	-0.04	-1.19	-1.49	PAPOLG	64895	0.33	0.7	1.33
XRCC1	7515	-0.29	-0.88	-1.49	FJX1	24147	-0.01	0.12	1.33
MC1R	4157	-0.19	-0.98	-1.49	CNNM3	26505	0.54	1.08	1.33
GPS2	2874	-0.56	-1.1	-1.49	STAMBPL1	57559	0.42	1.08	1.33
C9ORF125	84302	-0.64	-1.17	-1.49	MTHFD1L	25902	0.16	0.97	1.33
NDRG3	57446	-0.28	-0.52	-1.49	PHF5A	84844	0.39	0.95	1.33
RNF43	54894	-0.41	-0.72	-1.49	PWWP2A	114825	0.53	0.52	1.33
FERMT1	55612	-0.49	-0.64	-1.49	PPP2CB	5516	0.08	0.57	1.32
MNS1	55329	-0.4	-1.37	-1.49	RIOK3	8780	-0.27	0.55	1.32
C12ORF52	84934	-0.33	-1.11	-1.49	SRF	6722	-0.15	0.59	1.32
ZNF30	90075	-0.5	-0.71	-1.49	MKLN1	4289	0.28	0.76	1.32
TARBP1	6894	0.09	-1	-1.48	NUPL1	9818	0.16	0.62	1.32
RFX5	5993	0	-0.58	-1.48	NFKB2	4791	0.33	0.62	1.32
HMBS	3145	-0.2	-0.63	-1.48	MYEOV	26579	0.72	0.98	1.32
OXSM	54995	-0.46	-0.53	-1.48	HBEGF	1839	-0.01	0.35	1.31
C18ORF22	79863	0.01	-0.59	-1.48	TSC22D2	9819	0.36	0.37	1.31
ITFG3	83986	0.25	-0.3	-1.48	NR4A2	4929	1.03	0.74	1.31
MBLAC2	153364	-0.18	-0.14	-1.48	TCF7L2	6934	0.44	0.8	1.31
OAZ2	4947	0	-1.2	-1.47	CDKN1C	1028	0.55	0.81	1.31
DHCR7	1717	0.21	-1.31	-1.47	LOC100130721	100130721	0.39	1.18	1.31
ACAA1	30	-0.05	-0.62	-1.47	LOC100130996	100130996	0.18	0.61	1.31
CSK	1445	-0.47	-0.64	-1.47	SETD7	80854	-0.02	0.63	1.31
LOC644450	644450	-0.43	-0.48	-1.47	LOC387647	387647	-0.11	0.4	1.31
DPH5	51611	0	-0.33	-1.47	GATA3	2625	0.47	0.77	1.3
RTN4IP1	84816	-0.23	-0.61	-1.47	MT2A	4502	0.48	1.23	1.3
ATP6V1E2	90423	-0.08	-0.09	-1.47	OTUD7B	56957	0.4	0.73	1.3
PAFAH1B3	5050	-0.05	-0.92	-1.46	BOD1	91272	0.34	0.95	1.3
MYO5A	4644	-0.55	-1.23	-1.46	MED30	90390	0.41	0.85	1.3
SOX21	11166	0.47	-0.74	-1.46	TP53INP2	58476	0.25	0.73	1.3
CREB3L4	148327	-0.15	-0.92	-1.46	FLJ42969	441374	0.33	0.39	1.3

NAPEPLD	222236	0.14	-0.73	-1.46	SPEN	23013	0.12	0.65	1.29
FAM78A	286336	-0.19	-1.22	-1.46	NRAS	4893	0.4	0.85	1.29
C20ORF118	140711	-1.11	-1.22	-1.46	PSEN1	5663	0.12	0.89	1.29
SNPH	9751	0.26	-0.36	-1.45	GOLGA2	2801	0.08	0.57	1.29
CRYAB	1410	0.43	-1.02	-1.45	TLE3	7090	0.31	0.81	1.29
MIIP	60672	-0.43	-0.58	-1.45	DOHH	83475	0.22	0.65	1.29
FAM59A	64762	-0.69	-0.88	-1.45	QSOX2	169714	0.44	1	1.29
FBXO25	26260	-0.75	-1.28	-1.45	ZSWIM6	57688	0.07	0.27	1.29
METTL12	751071	-0.32	-0.52	-1.45	LOC645978	645978	-0.16	0.42	1.29
NCRNA00173	100287569	-0.35	-0.55	-1.45	MNT	4335	0.8	0.9	1.28
HSP90AB5P	442083	-0.09	-1.39	-1.45	RFX1	5989	0.78	0.78	1.28
GCLC	2729	-0.58	-1.18	-1.44	CXCL11	6373	-0.08	0.2	1.28
LTA4H	4048	0.08	-0.53	-1.44	CLK1	1195	0.9	0.97	1.28
ZC3H4	23211	-0.8	-0.74	-1.44	ZIC5	85416	0.49	0.89	1.28
NAT1	9	-0.59	-0.83	-1.44	CPNE8	144402	0.49	0.59	1.28
RASSF4	83937	-0.2	-0.6	-1.44	EIF2C2	27161	0.21	0.53	1.28
MTIF3	219402	0.06	-0.5	-1.44	WIPF1	7456	0.25	0.82	1.27
PEBP1	5037	0.02	-0.79	-1.43	SS18	6760	0.04	0.44	1.27
BPHL	670	-0.15	-1	-1.43	RYK	6259	0.05	0.71	1.27
POLDIP2	26073	-0.27	-0.83	-1.43	ARHGEF17	9828	0.06	0.72	1.27
MLPH	79083	-0.78	-0.78	-1.43	SPAG9	9043	0.15	0.37	1.27
ZNF771	51333	-0.29	-0.69	-1.43	PINK1	65018	0.36	1.04	1.27
IFT172	26160	-0.28	-0.45	-1.43	ATAD2B	54454	0.14	0.57	1.27
VSIG10L	147645	0.13	-0.94	-1.43	C1ORF69	200205	-0.25	0.21	1.27
TIGD2	166815	-0.74	-0.85	-1.43	C16ORF61	56942	0.32	0.82	1.27
LCMT2	9836	-1.2	-0.92	-1.42	MIS12	79003	-0.04	0.66	1.27
DZIP1	22873	-0.44	-1.01	-1.42	KLC3	147700	0.01	0.39	1.27
SLC4A11	83959	-0.62	-1.14	-1.42	VDR	7421	0.61	0.8	1.26
STOX2	56977	-0.76	-1.07	-1.42	BDNF	627	0.35	0.72	1.26
CACYBP	27101	-0.19	-1.15	-1.41	SMTN	6525	-0.02	0.32	1.26
KIAA0196	9897	-0.32	-0.84	-1.41	MCAM	4162	0.21	0.18	1.26

GPR162	27239	-0.02	-1.02	-1.41	FAF2	23197	-0.08	0.57	1.26
CPLX2	10814	-0.15	-0.74	-1.41	E2F5	1875	0.11	0.64	1.26
HYAL2	8692	0.31	-0.85	-1.41	C9ORF21	195827	0.28	0.87	1.26
DBNL	28988	-0.53	-1.02	-1.41	HIST4H4	121504	0.2	0.81	1.26
CLDN23	137075	-0.66	-0.66	-1.41	LOC728868	728868	0.33	0.61	1.26
PRSS36	146547	0.33	-0.2	-1.41	LOC730167	730167	0.21	0.96	1.26
ADCK5	203054	-0.25	-1.15	-1.41	SLC36A4	120103	0.51	1.18	1.26
GTF3A	2971	0.07	-0.43	-1.4	ZNRD1	30834	0.34	0.95	1.26
GALT	2592	-0.22	-0.76	-1.4	LOC389831	389831	0.17	0.36	1.26
CCDC6	8030	-0.51	-0.81	-1.4	ATG9A	79065	0.15	0.8	1.25
MSTO1	55154	-0.76	-1.24	-1.4	PCF11	51585	-0.28	0.45	1.25
NUBPL	80224	-0.1	-0.78	-1.4	RBBP6	5930	0.14	0.51	1.25
EMILIN2	84034	-0.42	-0.86	-1.4	DRD4	1815	0.56	1.09	1.25
DNAJC30	84277	-0.62	-0.93	-1.4	DLC1	10395	0.1	0.87	1.25
FAM81A	145773	0.02	-0.59	-1.4	UBTD1	80019	0.4	0.59	1.25
C11ORF74	119710	0.23	-0.44	-1.4	LOC57399	57399	0.55	0.95	1.25
NCOA7	135112	0.13	-0.32	-1.4	LOC100129110	100129110	0.14	0.29	1.25
FASTK	10922	-0.21	-0.67	-1.39	RPRD1B	58490	-0.34	0.48	1.25
TNFRSF1A	7132	0.55	-0.4	-1.39	DDX3X	1654	0.3	0.61	1.24
CETN3	1070	0.15	-0.28	-1.39	ZFP36	7538	0.8	0.53	1.24
TMEM159	57146	0.12	-0.56	-1.39	RYBP	23429	0.37	0.62	1.24
MRPL20	55052	-0.19	-1.04	-1.39	PLEKHO2	80301	0.07	0.18	1.24
LOC80154	80154	-0.01	-0.76	-1.39	ACYP1	97	0.7	1.23	1.24
CCT3	7203	-0.19	-1.22	-1.38	CBL	867	0.13	0.53	1.24
C5ORF13	9315	-0.17	-1.09	-1.38	CREM	1390	0.37	0.78	1.24
ARSE	415	-0.15	-0.51	-1.38	POGZ	23126	0.18	0.51	1.24
STX2	2054	-0.82	-1.13	-1.38	C20ORF4	25980	0.19	0.48	1.24
RARS2	57038	-0.34	-1.16	-1.38	CNNM4	26504	0.42	1	1.24
APBA2	321	-0.18	-0.72	-1.38	LINS1	55180	0.16	0.32	1.24
IFT81	28981	-0.02	-0.88	-1.38	FBXL12	54850	0.38	0.49	1.24
LOC100288578	100288578	-0.39	-0.69	-1.38	C5ORF32	84418	0.42	0.94	1.24

BBS2	583	0.28	-0.14	-1.38	PGM5P1	653394	-0.21	0.32	1.24
LOC100293352	100293352	-1.44	-1.29	-1.38	BECN1L1	441925	0.88	0.96	1.24
ADD3	120	-0.13	-0.85	-1.37	SHOC2	8036	0.2	0.57	1.23
DDX28	55794	-0.73	-0.85	-1.37	RAB30	27314	-0.26	0.36	1.23
ITGB4	3691	0.01	-0.05	-1.37	TUBB6	84617	0.08	0.28	1.23
ANKRD7	56311	-1.19	-1.01	-1.37	STARD13	90627	0.13	0.29	1.23
TSKU	25987	-0.18	-0.39	-1.37	ARPP19	10776	0.2	0.66	1.23
C14ORF132	56967	0.08	-0.74	-1.37	KIAA1644	85352	0.08	0.61	1.23
LOC100170939	100170939	-0.55	-0.59	-1.37	LOC100129138	100129138	0.46	0.8	1.23
MAPKAPK3	7867	-0.29	-0.93	-1.36	WDR20	91833	0.44	0.59	1.23
ACOT13	55856	0.15	-0.5	-1.36	IGF2BP1	10642	-0.04	0.35	1.23
HMMR	3161	-0.23	-0.72	-1.36	TMEM184B	25829	0.22	0.63	1.22
FSD1	79187	0.03	-0.52	-1.36	DGCR2	9993	0.46	0.62	1.22
HSPC157	29092	0.19	-0.52	-1.36	GSK3A	2931	0.38	0.48	1.22
C15ORF38	348110	-0.2	-0.79	-1.36	WBP4	11193	-0.36	0.6	1.22
EME1	146956	-0.53	-0.9	-1.36	TAPT1	202018	0.04	0.63	1.22
NOTCH2	4853	-0.34	-0.93	-1.35	KLB	152831	0.18	0.75	1.22
LEPREL2	10536	0.06	-0.43	-1.35	MIER1	57708	0.28	0.58	1.22
AGBL5	60509	-0.42	-0.88	-1.35	LOC100133862	100133862	0.11	0.26	1.22
FAM82B	51115	0.01	-0.43	-1.35	CKS1B	1163	0.23	0.91	1.21
FAM120AOS	158293	-0.71	-1.16	-1.35	CAV2	858	0.4	0.88	1.21
RFXANK	8625	-0.28	-0.7	-1.34	ELL	8178	0.22	0.36	1.21
B3GNT1	11041	0.55	0.19	-1.34	ADRB2	154	0.14	0.68	1.21
SLC27A2	11001	-0.01	-1.03	-1.34	NFKB1	4790	0.1	0.49	1.21
SCNM1	79005	-0.58	-0.98	-1.34	PARD6B	84612	0.39	0.76	1.21
PAAF1	80227	-0.22	-1.04	-1.34	DCP2	167227	0.01	0.56	1.21
SLC35E3	55508	-0.35	-0.73	-1.34	MAP3K1	4214	-0.03	0.45	1.21
LOH12CR1	118426	-0.12	-0.69	-1.34	TM9SF3	56889	0.22	0.71	1.21
RP5-1022P6.2	56261	-0.47	-0.78	-1.34	SLC39A1	27173	0.35	1.08	1.21
C9ORF140	89958	-0.31	-0.18	-1.34	KLHL15	80311	0.56	0.63	1.21
ZBED3	84327	-0.74	-0.5	-1.34	FAM19A3	284467	-0.13	0.09	1.21

F8A1	8263	-0.27	-0.61	-1.33	POLR2D	5433	-0.09	0.35	1.2
FEZ1	9638	-1.08	-1.19	-1.33	PCDH1	5097	0.59	1.01	1.2
AGGF1	55109	-0.9	-1.22	-1.33	JAG1	182	0.63	1	1.2
CCNI	10983	-0.19	-0.57	-1.33	HMGXB3	22993	-0.02	0.12	1.2
ORC3L	23595	-0.38	-0.92	-1.33	ETNK1	55500	0.74	0.52	1.2
TMEM97	27346	-0.33	-1.21	-1.33	SEMA4C	54910	0.35	0.62	1.2
FAM55C	91775	-0.19	-0.74	-1.33	GFM1	85476	0.13	0.8	1.2
EHD3	30845	-0.36	-0.51	-1.33	MYEF2	50804	0.01	0.65	1.2
ODF3L2	284451	-0.07	-0.6	-1.33	C10ORF35	219738	0.39	1.03	1.2
ZNF25	219749	-0.31	-0.84	-1.33	IKBIP	121457	0.32	0.81	1.2
FLJ45248	401472	0.14	-0.89	-1.33	C5ORF40	408263	0.54	0.61	1.2
PLEKHG4	25894	-0.02	-0.89	-1.33	LOC550112	550112	-0.6	0.52	1.2
IGBP1	3476	0.08	-0.77	-1.32	MAP2K1	5604	0.28	0.77	1.19
CDC16	8881	-0.72	-0.74	-1.32	RARA	5914	0.66	0.81	1.19
SFRS5	6430	-0.9	-0.85	-1.32	VNN2	8875	0.09	0.65	1.19
AKR1C1	1645	-0.16	-0.48	-1.32	EIF2B5	8893	0.03	0.68	1.19
APC2	10297	-0.02	-0.73	-1.32	RC3H1	149041	0.34	0.7	1.19
PNMA2	10687	-0.49	-0.66	-1.32	DUSP10	11221	0.19	0.56	1.19
ACAT2	39	0.13	-1.1	-1.32	BRF2	55290	0.36	0.74	1.19
PLA2G4A	5321	0.22	-0.64	-1.32	CA10	56934	0.24	0.21	1.19
GGTLC1	92086	-0.14	-0.49	-1.32	SH2D5	400745	-0.24	0.21	1.19
KIAA0485	57235	-0.1	-0.53	-1.32	BNIP1	662	0.14	0.82	1.18
MRPS33	51650	0.01	-0.46	-1.32	SCML2	10389	0.19	0.59	1.18
ARMCX1	51309	-0.41	-0.54	-1.32	DLX2	1746	0.14	0.05	1.18
NPEPL1	79716	-0.07	-0.38	-1.32	DDAH1	23576	0.05	0.19	1.18
MREG	55686	-1.16	-0.9	-1.32	CCDC28A	25901	0.36	0.7	1.18
WNT3	7473	-0.08	-0.99	-1.32	AVL9	23080	0.14	0.82	1.18
LCMT1	51451	0	-0.63	-1.32	SATB2	23314	0.52	0.69	1.18
GANC	2595	-0.25	-0.98	-1.32	GGNBP2	79893	0.36	0.52	1.18
FILIP1	27145	-0.14	-0.54	-1.32	ALG14	199857	0.34	0.72	1.18
TCEAL8	90843	0.12	-0.53	-1.32	ANKRD33B	651746	0.42	0.8	1.18

LOC643988	643988	0.03	-1.29	-1.32	ZCCHC3	85364	0.32	0.68	1.18
STAT1	6772	0.24	-0.64	-1.31	ARL5C	390790	0.03	0.31	1.18
RASSF2	9770	-0.26	-0.95	-1.31	C21ORF121	150142	0.06	0.25	1.18
HISPPD1	23262	-0.02	-0.39	-1.31	CTDSPL2	51496	0.01	0.46	1.18
NDUFAF3	25915	-0.01	-0.98	-1.31	VKORC1L1	154807	0.28	0.53	1.18
ZNF688	146542	-0.43	-0.98	-1.31	PXN	5829	0.31	0.7	1.17
ARRB1	408	-0.46	-1.19	-1.31	KIAA0408	9729	0.18	0.79	1.17
C8ORF30A	51236	-0.41	-0.72	-1.31	CRLF1	9244	0.28	0.79	1.17
ZNF839	55778	-0.55	-0.91	-1.31	ANKLE2	23141	-0.17	0.05	1.17
THNSL1	79896	-0.8	-1.02	-1.31	PHLPP2	23035	0.07	0.36	1.17
PPA2	27068	0.02	-0.48	-1.31	PTRH2	51651	0.01	0.34	1.17
SCARNA17	677769	-0.48	-0.81	-1.31	EDA2R	60401	0.06	0.61	1.17
LOC100133487	100133487	0.56	-0.48	-1.31	DPH3	285381	0.1	0.77	1.17
NAV1	89796	0.58	-0.29	-1.31	RRM2B	50484	0.39	0.91	1.17
PLCE1	51196	-0.08	-0.74	-1.3	ZNF697	90874	0.3	0.67	1.17
PECR	55825	0.07	-0.58	-1.3	WWTR1	25937	0.62	0.36	1.16
SIRT3	23410	0.05	-0.54	-1.3	E2F4	1874	-0.09	0.63	1.16
C7ORF70	84792	-0.34	-0.62	-1.3	SMAD3	4088	0.2	0.69	1.16
C9ORF103	414328	-0.4	-0.71	-1.3	ZNF136	7695	-0.16	0.48	1.16
DTNBP1	84062	-0.53	-0.7	-1.3	GAR1	54433	-0.06	0.45	1.16
LOC222070	222070	-0.35	-0.75	-1.3	C10ORF110	55853	-0.67	0.42	1.16
PRELID2	153768	-0.29	-0.72	-1.3	ARL8A	127829	0.3	0.72	1.16
ZBTB47	92999	-0.22	-0.43	-1.3	KSR2	283455	0.42	1	1.16
PDCD2	5134	-0.41	-0.49	-1.29	LIX1L	128077	0.59	0.96	1.16
HOXB5	3215	-0.3	-0.81	-1.29	SKA2	348235	0.23	0.83	1.16
SLC3A1	6519	-0.46	-1.08	-1.29	ELOVL7	79993	0.2	0.34	1.16
HOXA7	3204	-0.28	-0.44	-1.29	LOC100127961	100127961	0.28	0.31	1.16
CDON	50937	0.06	-0.66	-1.29	NHS	4810	-0.22	0.24	1.16
C17ORF62	79415	-0.46	-1.13	-1.29	OR4E2	26686	-0.09	-0.06	1.16
ZDHHC6	64429	-0.43	-1.19	-1.29	HCCS	3052	0.07	0.68	1.15
TFCP2L1	29842	-0.92	-1.21	-1.29	LARS2	23395	0.31	0.46	1.15

BAT4	7918	-0.61	-0.87	-1.29	SCG2	7857	0.52	0.95	1.15
VPS36	51028	-0.11	-0.74	-1.29	WDR45L	56270	-0.18	0.42	1.15
PYDC1	260434	-0.09	-0.44	-1.29	ZFP36L1	677	0.4	0.56	1.15
LOC729421	729421	-0.18	-0.01	-1.29	PTPLB	201562	0.16	0.68	1.15
PEX11B	8799	-0.21	-0.66	-1.28	LIN7B	64130	0.43	0.37	1.15
SEMA3B	7869	-0.27	-0.74	-1.28	DOLPP1	57171	0.35	0.67	1.15
SLC22A3	6581	-0.41	-1.15	-1.28	TMEM126A	84233	0.15	0.57	1.15
PCYT2	5833	-0.09	-1.12	-1.28	B3GAT2	135152	0.15	0.39	1.15
SEC14L4	284904	-0.88	-1.15	-1.28	KLHL21	9903	-0.59	0.42	1.14
IL22RA1	58985	-0.44	-1.01	-1.28	RAMP3	10268	0.55	0.75	1.14
LOC283861	283861	-0.56	-0.98	-1.28	GFRA1	2674	0.34	0.52	1.14
SCARNA12	677777	-0.16	-0.9	-1.28	CFLAR	8837	0.03	0.53	1.14
LOC147727	147727	0.2	-0.53	-1.28	EHD4	30844	0.14	0.6	1.14
RDM1	201299	0.23	-0.95	-1.28	FAM18B	51030	0.3	0.78	1.14
MRPL40	64976	0	-0.71	-1.27	SLC25A22	79751	0.28	0.59	1.14
GALK1	2584	0.1	-0.38	-1.27	GLTP	51228	0.27	0.66	1.14
BRE	9577	-0.15	-0.75	-1.27	HSPBAP1	79663	0.25	0.77	1.14
GABRA5	2558	-0.15	-0.7	-1.27	C1ORF128	57095	-0.03	0.49	1.14
TELO2	9894	-0.62	-1.18	-1.27	FBXO33	254170	0.33	0.62	1.14
GINS4	84296	-0.46	-0.62	-1.27	ZBTB2	57621	0.17	0.51	1.14
FZD8	8325	0.55	0.27	-1.27	ZNF776	284309	-0.04	0.5	1.14
RNASEN	29102	-0.3	-0.72	-1.27	FAM161B	145483	-0.25	-0.06	1.14
NARS2	79731	-0.04	-0.22	-1.27	OR5J2	282775	-0.28	0.04	1.14
CISH	1154	0.05	-0.83	-1.27	BZW1	9689	0.48	0.34	1.13
SBK1	388228	-0.34	-1.08	-1.27	NCBP2	22916	0.12	0.64	1.13
SCRN2	90507	-0.02	-0.61	-1.27	LYST	1130	-0.09	0.05	1.13
HOXD8	3234	0.24	-0.26	-1.27	LSM1	27257	0.24	0.74	1.13
KRTAP19-2	337969	0.32	-0.12	-1.27	ACVR2A	92	0.22	0.45	1.13
LOC100131829	100131829	0.2	-0.64	-1.27	FCER2	2208	-0.25	-0.1	1.13
FAM20C	56975	0.13	-0.19	-1.27	GAL	51083	0.54	0.86	1.13
FLJ27352	145788	0.15	-0.13	-1.27	PMP22	5376	0.48	0.54	1.13

LOC120364	120364	0.1	-0.71	-1.27	KBTBD2	25948	0.26	0.55	1.13
ZNF252	286101	-1.02	-0.82	-1.27	COL4A3BP	10087	0.08	0.5	1.13
MPG	4350	0.04	-0.27	-1.26	LOC390595	390595	0.25	0.88	1.13
TUB	7275	-0.17	-0.57	-1.26	PTBP1	5725	0.15	0.68	1.12
FLJ40330	645784	-0.52	-0.57	-1.26	TP53BP2	7159	-0.17	0.17	1.12
SIDT2	51092	-0.04	-1.14	-1.26	CDKN2D	1032	0.68	0.89	1.12
C1ORF35	79169	0.12	-0.41	-1.26	RBM9	23543	0.19	0.62	1.12
DTWD2	285605	-0.3	-0.28	-1.26	HECA	51696	0.18	0.76	1.12
LOC344887	344887	-0.41	-0.29	-1.26	CHD7	55636	-0.22	0.41	1.12
ACAD9	28976	-0.28	-1.25	-1.26	MOBKL2B	79817	0.01	0.7	1.12
DOCK11	139818	-0.2	-1	-1.26	SERTAD3	29946	0.66	0.66	1.12
LOC100132850	100132850	-1.09	-1.01	-1.26	B3GALTL	145173	0.5	0.71	1.12
AIMP2	7965	-0.43	-0.56	-1.25	FAM177A1	283635	0.05	0.68	1.12
EPS8	2059	-0.34	-0.89	-1.25	CCDC39	339829	0.2	0.32	1.12
NSDHL	50814	-0.26	-1.03	-1.25	BIRC2	329	0.24	0.5	1.11
C9ORF3	84909	-0.23	-1.18	-1.25	KLF9	687	0.73	0.99	1.11
C14ORF159	80017	0.02	-0.91	-1.25	JUND	3727	0.78	0.73	1.11
FAM108A1	81926	-0.18	-0.83	-1.25	DDX52	11056	-0.02	0.51	1.11
C19ORF54	284325	-0.54	-0.73	-1.25	ORC6L	23594	-0.26	0.16	1.11
TUBBP5	643224	0.44	0	-1.25	MYL12B	103910	0.12	0.57	1.11
C1ORF59	113802	-0.15	-0.58	-1.25	MIDN	90007	0.34	0.31	1.11
LRRK2	120892	-0.13	-0.64	-1.25	DKFZP686D0853	401613	-0.31	0.08	1.11
PTGR1	22949	0.09	-0.65	-1.25	TES	26136	-0.04	0.51	1.1
COQ5	84274	-0.08	-0.42	-1.25	AHR	196	0.03	0.18	1.1
LOC400987	400987	-0.44	-0.46	-1.25	LMNA	4000	0.14	0.44	1.1
LOC390282	390282	0.02	-0.2	-1.25	CRCP	27297	-0.22	0.2	1.1
GTF2I	2969	-0.29	-1.02	-1.24	POFUT2	23275	-0.18	0.27	1.1
PYGB	5834	-0.16	-0.46	-1.24	CAV3	859	0.35	0.62	1.1
AHSA1	10598	-0.31	-1.05	-1.24	SPATA2L	124044	0.48	0.67	1.1
ARHGAP1	392	-0.21	-1	-1.24	GK2	2712	0.47	0.77	1.1
PDK2	5164	-0.08	-0.88	-1.24	TOLLIP	54472	0.06	0.55	1.1

MMD	23531	0	-0.71	-1.24	C9ORF167	54863	0.49	1.02	1.1
CAST	831	-0.28	-1.04	-1.24	C12ORF73	728568	0.45	0.71	1.1
METTL7A	25840	0.07	-0.7	-1.24	FAM196B	100131897	0.29	0.69	1.1
ANKRD36	375248	-0.63	-0.59	-1.24	MIER3	166968	0.19	0.45	1.1
EVL	51466	0.19	-0.63	-1.24	C7ORF40	285958	-0.38	-0.11	1.1
FLJ10357	55701	-0.34	-0.9	-1.24	LOC642635	642635	0.82	0.77	1.1
FRAS1	80144	-0.44	-1.12	-1.24	NFYC	4802	0.07	0.62	1.09
C17ORF70	80233	-0.25	-0.66	-1.24	TMED1	11018	0.42	0.98	1.09
ZNF324B	388569	-0.35	-0.78	-1.24	CUGBP1	10658	0.16	0.41	1.09
C7ORF27	221927	-0.26	-0.6	-1.24	LSM6	11157	0.23	0.74	1.09
KRBA1	84626	-0.55	-0.88	-1.24	ARHGEF15	22899	0.31	0.52	1.09
LOC100131000	100131000	-0.15	-0.81	-1.24	ZC3H11A	9877	-0.03	0.47	1.09
LOC100128563	100128563	-0.27	-0.51	-1.24	SERPINB9	5272	0.63	0.71	1.09
SSBP4	170463	-0.28	-0.52	-1.24	KIAA0090	23065	0.32	0.79	1.09
LOC100133008	100133008	0.25	-0.1	-1.24	MFAP3	4238	-0.13	0.53	1.09
MYO10	4651	-0.33	-0.77	-1.23	WBP11	51729	0.37	0.39	1.09
TUSC4	10641	-0.25	-0.71	-1.23	GOLT1B	51026	0.12	0.56	1.09
GOLIM4	27333	0.12	-0.5	-1.23	C10ORF88	80007	0.2	0.72	1.09
MYL5	4636	-0.25	-1.11	-1.23	CCDC50	152137	-0.04	0.53	1.09
CYB561D2	11068	-0.09	-0.81	-1.23	SRPR	6734	0.32	0.77	1.08
FGD2	221472	-1.05	-0.94	-1.23	MYH11	4629	0.37	0.2	1.08
MRPL24	79590	0.07	-0.46	-1.23	RELA	5970	0.03	0.62	1.08
PGLS	25796	0.01	-0.49	-1.23	PHF1	5252	0.62	0.88	1.08
GDAP1	54332	-0.73	-1.16	-1.23	ID3	3399	0.27	0.23	1.08
BIVM	54841	-0.3	-0.85	-1.23	SLC12A4	6560	0.01	0.35	1.08
TCAG7.1196	728743	-0.25	-0.74	-1.23	FAM168B	130074	0.01	0.5	1.08
FLJ33630	644873	-0.65	-0.51	-1.23	PCID2	55795	-0.11	0.35	1.08
FAM127C	441518	0.03	-0.46	-1.23	KIRREL	55243	-0.03	0.36	1.08
BTBD16	118663	0.11	-0.71	-1.23	LOC100128130	100128130	0.45	0.62	1.08
FBXO15	201456	0.04	-0.93	-1.23	SYS1	90196	0.23	0.62	1.08
FRG1B	284802	-0.15	-0.93	-1.23	TCAM1	146771	0.5	0.41	1.08

RNASEH2A	10535	0.03	-0.69	-1.22	LOC374890	374890	0.64	0.8	1.08
SURF1	6834	-0.23	-0.74	-1.22	M6PR	4074	0.07	0.48	1.07
SLC12A2	6558	0.07	-0.83	-1.22	CYTH1	9267	-0.13	0.52	1.07
ICK	22858	-0.64	-0.9	-1.22	SMURF2	64750	0.44	0.88	1.07
DDC	1644	-0.5	-1.19	-1.22	PTTG3P	26255	0.44	0.68	1.07
DZIP3	9666	-0.29	-0.42	-1.22	STX6	10228	0.13	0.31	1.07
MXD4	10608	-0.11	-0.89	-1.22	FBXL18	80028	-0.31	0.43	1.07
DARS2	55157	-0.01	-0.77	-1.22	CPSF7	79869	0.32	0.54	1.07
MUS81	80198	-0.74	-1.18	-1.22	NOP10	55505	0.21	0.6	1.07
FAM134B	54463	-0.53	-0.97	-1.22	C12ORF35	55196	0.05	0.68	1.07
THTPA	79178	-0.04	-0.92	-1.22	TMEM39A	55254	0.18	0.37	1.07
MSRB2	22921	-0.24	-0.63	-1.22	C14ORF138	79609	0.02	0.39	1.07
AMDHD2	51005	-0.16	-0.6	-1.22	THAP9	79725	0.23	0.58	1.07
RNF141	50862	-0.28	-0.47	-1.22	AP1S3	130340	0.4	1.02	1.07
SYTL2	54843	-0.16	-0.91	-1.22	RG9MTD2	93587	0.4	0.72	1.07
LOC91450	91450	-0.4	-0.74	-1.22	SLC30A7	148867	0.05	0.45	1.07
NT5DC1	221294	-0.11	-0.85	-1.22	FIGN	55137	-0.03	0.29	1.07
ARV1	64801	-0.32	-0.42	-1.22	RNF41	10193	0.49	0.84	1.06
BTBD12	84464	-0.39	-0.37	-1.22	MEF2D	4209	0.48	0.77	1.06
MMAB	326625	-0.72	-0.81	-1.22	PDGFA	5154	0.3	0.91	1.06
EIF3F	8665	-0.1	-0.23	-1.21	BTG3	10950	0.3	0.45	1.06
HSPD1	3329	-0.18	-1.17	-1.21	DEGS1	8560	0.32	0.54	1.06
TMEM66	51669	0.02	-0.72	-1.21	STAT3	6774	0.24	0.33	1.06
JUP	3728	-0.05	-0.47	-1.21	TGFB2	7042	0.27	0.34	1.06
LANCL1	10314	0.09	-0.73	-1.21	B4GALT3	8703	0.41	0.87	1.06
ACY1	95	0.05	-0.92	-1.21	ZEB1	6935	0.06	0.41	1.06
MYOM2	9172	-0.3	-0.88	-1.21	TOR1AIP1	26092	0.38	0.66	1.06
CHMP6	79643	-0.25	-0.56	-1.21	PPP1R14B	26472	0.13	0.55	1.06
TMEM160	54958	0.13	-0.58	-1.21	C16ORF80	29105	-0.15	0.4	1.06
TRIM45	80263	-0.1	-0.52	-1.21	CAPRIN2	65981	0.23	0.43	1.06
C14ORF126	112487	-0.06	-0.58	-1.21	DRAM1	55332	0.36	0.56	1.06

LOC100129596	100129596	0.16	-0.19	-1.21	VPS37C	55048	-0.19	0.42	1.06
LOC727916	727916	0.14	-0.53	-1.21	ING5	84289	0.27	0.8	1.06
MLKL	197259	-0.71	-0.61	-1.21	NIPAL1	152519	-0.06	0.53	1.06
AP2M1	1173	-0.09	-0.91	-1.2	CWC22	57703	0.17	0.43	1.06
FIBP	9158	-0.26	-0.62	-1.2	EIF1AD	84285	0.01	0.08	1.06
SC65	10609	-0.06	-0.31	-1.2	GNAQ	2776	0.13	0.49	1.05
SPINK1	6690	-1.01	-1.1	-1.2	JAKMIP2	9832	0.19	0.41	1.05
MSH2	4436	-0.41	-1.01	-1.2	PCTK2	5128	0.25	0.4	1.05
ZNF32	7580	-0.26	-0.92	-1.2	PTS	5805	0.2	0.74	1.05
TBC1D2B	23102	-0.68	-1.09	-1.2	VEGFC	7424	0.45	0.93	1.05
SFRS2B	10929	-0.69	-1	-1.2	ARHGAP19	84986	0.22	0.28	1.05
DERA	51071	-0.17	-0.52	-1.2	KRT35	3886	0.89	1.02	1.05
NRN1	51299	-0.63	-0.83	-1.2	FA2H	79152	0.67	0.88	1.05
IFT52	51098	-0.19	-0.68	-1.2	ASFMR1	100126270	0.5	0.73	1.05
ABCA7	10347	-0.18	-0.98	-1.2	ZNF398	57541	0.36	0.86	1.05
PGAP3	93210	-0.14	-0.98	-1.2	PEX19	5824	0.21	0.75	1.04
ZNF512B	57473	-0.43	-0.72	-1.2	RGS2	5997	0.55	0.32	1.04
S1PR3	1903	0	-0.71	-1.2	STK3	6788	0.48	0.54	1.04
TC2N	123036	-0.01	-0.59	-1.2	NEK7	140609	0.14	0.45	1.04
ZCCHC17	51538	-0.27	-0.83	-1.2	SLC36A1	206358	-0.06	0.46	1.04
LOC388588	388588	0.24	-0.52	-1.2	ETS1	2113	0.17	0	1.04
IRAK1BP1	134728	-0.16	0.04	-1.2	AKIRIN1	79647	0.1	0.48	1.04
CENPBD1	92806	-0.7	-0.9	-1.2	FREQ	23413	0.47	0.75	1.04
MATN2	4147	0.22	-0.6	-1.19	COMMD10	51397	0.47	0.59	1.04
GATM	2628	-0.04	-0.96	-1.19	GMEB1	10691	0.21	0.75	1.04
NLE1	54475	-0.33	-0.87	-1.19	MAP3K2	10746	0.41	0.66	1.04
CEBPA	1050	-0.67	-0.81	-1.19	AMMECR1L	83607	0.31	0.59	1.04
FGB	2244	-0.17	-1.18	-1.19	C10ORF53	282966	-0.12	0.07	1.04
STEAP1	26872	-0.35	-0.68	-1.19	C15ORF57	90416	-0.05	0.24	1.04
MKL2	57496	-0.32	-0.93	-1.19	EGFR	1956	0.36	0.99	1.03
C14ORF169	79697	-0.39	-0.77	-1.19	KIAA0247	9766	0.38	0.44	1.03

CYB5R2	51700	0.08	-1	-1.19	APOE	348	0.32	0.67	1.03
LOC282997	282997	0.08	-0.93	-1.19	ZNF134	7693	-0.42	0.09	1.03
C10ORF58	84293	-0.11	-0.81	-1.19	HBP1	26959	0.43	0.52	1.03
HAUS1	115106	-0.13	-0.74	-1.19	KLHL25	64410	-0.66	-0.03	1.03
HEATR5A	25938	-0.05	-0.61	-1.19	AKT3	10000	0.22	0.82	1.03
LOC728739	728739	-0.27	-0.45	-1.19	LOC144438	144438	0.45	0.57	1.03
ZNF618	114991	-0.23	-0.38	-1.19	ADAM6	8755	0.44	0.45	1.03
LOC100130711	100130711	0.26	-0.16	-1.19	ARRDC2	27106	-0.1	0.39	1.03
TMEM116	89894	-0.05	-0.35	-1.19	NEURL3	93082	-0.04	0.03	1.03
LOC100129532	100129532	-0.95	-1.14	-1.19	S100A11	6282	0.2	0.78	1.02
ALDH3A2	224	-0.33	-0.55	-1.18	SLC16A1	6566	0.08	0.69	1.02
TPD52L1	7164	-0.17	-0.6	-1.18	POLR2K	5440	0.19	0.61	1.02
C17ORF75	64149	-0.16	-0.61	-1.18	SOX9	6662	1.04	0.89	1.02
BDKRB1	623	0.83	-0.08	-1.18	SBNO2	22904	0.31	0.66	1.02
SHMT1	6470	-0.14	-0.79	-1.18	ZNF592	9640	0.46	0.94	1.02
BCKDHB	594	0.06	-0.78	-1.18	ALDH3B2	222	0.43	0.48	1.02
BRMS1	25855	-0.32	-0.38	-1.18	FOXN2	3344	0.48	0.63	1.02
ASNSD1	54529	-0.36	-0.81	-1.18	DPY19L1	23333	0.35	0.86	1.02
TMEM121	80757	-0.28	-1.02	-1.18	RSBN1	54665	0.5	0.57	1.02
C11ORF86	254439	0.23	0.34	-1.18	GUCA1B	2979	0.3	0.9	1.02
C4ORF33	132321	0.06	-0.75	-1.18	TNKS2	80351	0.21	0.54	1.02
INTS3	65123	-0.4	-1.01	-1.17	C6ORF35	729515	-0.16	0.44	1.02
PDE4B	5142	0.32	-0.74	-1.17	LOC643371	643371	0.39	0.57	1.02
TBC1D9B	23061	-0.45	-1.01	-1.17	MRPL42P5	359821	-0.01	0.51	1.02
PFKM	5213	-0.17	-0.69	-1.17	ZC3H12C	85463	-0.05	0.5	1.02
RBL2	5934	-0.23	-0.73	-1.17	ZFYVE1	53349	0.05	0.24	1.02
TBCK	93627	-0.31	-0.76	-1.17	ZNF462	58499	-0.77	-0.1	1.02
CPNE4	131034	0.28	-0.11	-1.17	FBXO22OS	692224	0.72	0.86	1.02
LOC647086	647086	0.08	-0.38	-1.17	LOC400027	400027	0.57	0.84	1.02
SLITRK6	84189	0.46	-0.09	-1.17	MET	4233	-0.08	0.48	1.01
FAM69B	138311	-0.05	-0.29	-1.17	ATG12	9140	0.11	0.5	1.01

ZFP14	57677	-0.23	-0.09	-1.17
DHFR	1719	-0.11	-1.11	-1.16
TTC37	9652	-0.05	-0.36	-1.16
FAM189B	10712	0.06	-0.34	-1.16
NDST2	8509	0.09	-0.34	-1.16
PTPN13	5783	-0.41	-0.69	-1.16
EPB49	2039	-0.04	-0.64	-1.16
PSIP1	11168	-0.34	-0.84	-1.16
APEX1	328	-0.06	-0.58	-1.16
TNFRSF21	27242	-0.34	-0.96	-1.16
MRS2	57380	-0.38	-0.66	-1.16
SLC27A5	10998	0.34	-0.78	-1.16
TBC1D16	125058	-0.03	-0.49	-1.16
GLI4	2738	-0.24	-0.56	-1.16
ATOH8	84913	-0.4	-0.22	-1.16
LRFN1	57622	-0.06	-0.37	-1.16
LOC151162	151162	0.12	-0.24	-1.16
VPS26B	112936	0.08	-0.49	-1.16
ADPRHL1	113622	0.15	-0.84	-1.16
KIF26A	26153	-0.08	-0.87	-1.16
CYP4X1	260293	-1.04	-0.81	-1.16
WDR31	114987	-0.65	-0.69	-1.16
LOC100147773	100147773	-0.96	-1.04	-1.16
UBXN1	51035	-0.17	-0.78	-1.15
CD44	960	0.07	-0.58	-1.15
ADCY3	109	-0.05	-0.63	-1.15
CACNB3	784	-0.06	-0.92	-1.15
THAP7	80764	-0.2	-0.42	-1.15
DAK	26007	0.12	-0.56	-1.15
ASCC1	51008	-0.06	-0.15	-1.15
ANKRD36B	57730	-0.43	-0.43	-1.15
NMT2	9397	-0.28	-0.06	1.01
STK17B	9262	0.63	0.51	1.01
MMP25	64386	0.36	0.35	1.01
PTRF	284119	-0.1	0.23	1.01
PTTG2	10744	0.57	0.6	1.01
B3GNT2	10678	0.32	0.58	1.01
C13ORF37	440145	0.49	0.7	1.01
FLJ16734	641928	0.4	0.67	1.01
LOC100286937	100286937	0.18	0.55	1.01
C6ORF150	115004	0.59	0.54	1.01
LOC100128950	100128950	0.75	0.75	1.01
SLC39A7	7922	0.38	0.57	1
RNF24	11237	0.19	0.48	1
ALG13	79868	0.14	0.72	1
GRIN2D	2906	0.86	0.78	1
POU5F1	5460	0.43	0.77	1
CLIC1	1192	-0.05	0.47	1
TMEM87A	25963	0.12	0.58	1
MAPKSP1	8649	0.11	0.45	1
RNF111	54778	0.32	0.67	1
SLC24A3	57419	0.22	0.19	1
MYLIP	29116	0.4	0.88	1
UBE2R2	54926	0.43	0.75	1

AXIN2	8313	-0.41	-0.93	-1.15
MND1	84057	0.1	-0.6	-1.15
ST6GAL2	84620	-0.08	-0.43	-1.15
ZBTB45	84878	-0.18	-0.47	-1.15
PABPC4L	132430	-0.64	-0.62	-1.15
DAB2	1601	-0.8	-0.88	-1.14
EFNB1	1947	-0.2	-0.68	-1.14
SKP2	6502	0.26	-0.42	-1.14
PRKX	5613	-0.26	-0.5	-1.14
NAP1L1	4673	-0.02	-0.49	-1.14
SLC22A18	5002	0.05	-0.47	-1.14
PLCD1	5333	0.17	-0.76	-1.14
PRKCQ	5588	-0.56	-0.93	-1.14
BDH1	622	-0.08	-0.78	-1.14
TMCC1	23023	-0.52	-0.96	-1.14
TEX264	51368	0.02	-0.16	-1.14
KDELC1	79070	0.02	-0.81	-1.14
C2ORF74	339804	-0.48	-0.64	-1.14
DTD1	92675	0.01	-0.52	-1.14
LOC91948	91948	0.03	-0.63	-1.14
POLR3H	171568	-0.55	-0.82	-1.14
LOC653113	653113	-0.49	-0.58	-1.14
PLCXD3	345557	0.23	-0.41	-1.14
RCCD1	91433	-0.14	-0.1	-1.14
LOC100008588	100008588	0.34	-0.76	-1.14
PDK3	5165	-0.2	-0.73	-1.13
METTL13	51603	-0.37	-0.57	-1.13
MPP2	4355	-0.44	-1.05	-1.13
PAPSS1	9061	-0.15	-0.85	-1.13
TGFB1I1	7041	-0.15	-0.67	-1.13
FAM192A	80011	-0.28	-0.84	-1.13

MYO19	80179	0.05	-0.36	-1.13
LOC153684	153684	-0.5	-0.77	-1.13
MIF4GD	57409	-0.08	-0.55	-1.13
C1ORF220	400798	-0.42	-0.52	-1.13
SH3PXD2B	285590	-0.12	-0.34	-1.13
C6ORF154	221424	0.24	-0.34	-1.13
CACNG6	59285	0.21	-0.09	-1.13
DKFZP434H168	26077	0.11	-0.87	-1.13
C1ORF211	148645	-0.82	-0.98	-1.13
HNRNPD	3184	-0.23	-0.88	-1.12
WFS1	7466	0.24	-0.3	-1.12
ACD	65057	-0.51	-0.86	-1.12
F2RL2	2151	-0.28	-0.7	-1.12
OSGEP	55644	-0.11	-0.88	-1.12
MAB21L2	10586	0.42	0	-1.12
NIPA2	81614	-0.66	-0.87	-1.12
ALDH1A1	216	-0.07	-0.77	-1.12
GGCT	79017	-0.05	-0.59	-1.12
CCDC90B	60492	-0.17	-0.5	-1.12
ACN9	57001	0.26	0.02	-1.12
MYBBP1A	10514	-0.23	-0.69	-1.12
ZNF232	7775	-0.58	-0.55	-1.12
CEP63	80254	-0.04	-0.76	-1.12
MAVS	57506	-0.02	-0.07	-1.12
C17ORF72	92340	-0.5	-1.07	-1.12
CCDC77	84318	-0.1	-0.78	-1.12
CPSF3	51692	-0.42	-0.92	-1.12
LOC729088	729088	-0.27	-0.4	-1.12
CCDC104	112942	0.12	-0.62	-1.12
VSTM2L	128434	0	-0.74	-1.12
KIAA0317	9870	-0.13	-0.76	-1.11

SLC37A4	2542	0.15	-0.24	-1.11
CORO2A	7464	0.49	0.02	-1.11
FXN	2395	-0.17	-0.53	-1.11
LRRC23	10233	-0.31	-0.71	-1.11
SLC16A5	9121	-0.13	0.04	-1.11
PLA2G16	11145	-0.16	-0.62	-1.11
N6AMT1	29104	-0.41	-0.65	-1.11
MLST8	64223	-0.22	-0.36	-1.11
C6ORF120	387263	-0.08	-0.2	-1.11
LOC100128108	100128108	-0.56	-0.77	-1.11
TCHP	84260	-0.63	-0.68	-1.11
BOK	666	-0.79	-1.06	-1.11
USO1	8615	-0.06	-0.53	-1.1
HIRIP3	8479	-0.03	-0.36	-1.1
DLG3	1741	-0.03	-0.12	-1.1
PDLIM1	9124	0.09	-0.75	-1.1
C18ORF10	25941	0.06	-0.84	-1.1
C21ORF59	56683	-0.19	-0.49	-1.1
RNASEH1	246243	-0.57	-0.94	-1.1
SDPR	8436	-0.33	-0.13	-1.1
ELL3	80237	-0.55	-1.05	-1.1
PLK1S1	55857	-0.75	-0.7	-1.1
UBE2Q2	92912	-0.38	-0.74	-1.1
LOC100290344	100290344	0.23	-0.1	-1.1
NUDT22	84304	-0.11	-0.23	-1.1
SLC5A11	115584	0.06	-1.02	-1.1
KIAA1908	114796	-0.8	-0.97	-1.1
KLK9	284366	-0.92	-0.79	-1.1
SLCO2B1	11309	0.03	-0.99	-1.09
MID1	4281	-0.35	-0.73	-1.09
APEX2	27301	-0.36	-0.91	-1.09

PDE8A	5151	-0.56	-0.95	-1.09
LOC100289097	100289097	-0.13	-0.59	-1.09
HEATR3	55027	-0.4	-0.72	-1.09
C22ORF40	150383	-0.36	-0.58	-1.09
FUK	197258	0.03	-0.5	-1.09
RABL2A	11159	-0.45	-0.64	-1.09
MAF1	84232	-0.11	-0.37	-1.09
TSEN54	283989	0.32	-0.05	-1.09
TMEM129	92305	0.19	-0.01	-1.09
PLAT	5327	-0.05	-0.86	-1.08
SYNE2	23224	-0.43	-0.97	-1.08
TBL1Y	90665	-0.34	-0.23	-1.08
ZNF395	55893	-0.41	-0.67	-1.08
SCARA3	51435	0.07	-0.66	-1.08
MANSC1	54682	0.05	-0.39	-1.08
RNFT2	84900	-0.12	-0.49	-1.08
AFAP1L2	84632	-0.47	-0.85	-1.08
C8ORF47	203111	-0.37	-0.82	-1.08
TSHZ1	10194	0.1	-0.4	-1.08
WDR24	84219	-0.04	0.04	-1.08
TMEM141	85014	-0.03	-1.02	-1.08
USP40	55230	-0.53	-0.65	-1.08
ZNF19	7567	-0.62	-0.76	-1.08
SNX17	9784	-0.16	-0.45	-1.07
MMP15	4324	0.38	-0.16	-1.07
TTC3	7267	-0.15	-0.77	-1.07
ABCC5	10057	0.24	-0.3	-1.07
MBD4	8930	-0.41	-0.8	-1.07
DCLRE1A	9937	-0.81	-0.98	-1.07
NBEAL2	23218	-0.18	-0.21	-1.07
ZNF248	57209	-0.45	-0.58	-1.07

FAM179B	23116	-0.49	-1.01	-1.07
PLCH1	23007	-0.33	-0.7	-1.07
DSCC1	79075	-0.19	-0.65	-1.07
C20ORF27	54976	0.03	-0.37	-1.07
TMC5	79838	-0.04	-0.65	-1.07
ACOXL	55289	0.02	-0.46	-1.07
PAOX	196743	-0.31	-0.69	-1.07
C14ORF72	145200	-0.13	-0.49	-1.07
HEJ1	94236	-0.5	-1.06	-1.07
DPRXP4	503645	0.24	-0.04	-1.07
CCDC122	160857	-0.51	-0.59	-1.07
LOC100133915	100133915	-1.29	-1.01	-1.07
HADH	3033	0.1	-0.55	-1.06
AKR1A1	10327	0.04	-0.02	-1.06
RFC5	5985	-0.36	-1	-1.06
HOXC6	3223	-0.34	-0.84	-1.06
RAD51L3	5892	-0.35	-0.82	-1.06
TENC1	23371	-0.3	-0.72	-1.06
MEGF9	1955	-0.05	-0.63	-1.06
MAN1A2	10905	-0.21	-0.46	-1.06
AAAS	8086	0.23	-0.54	-1.06
LGR4	55366	0.05	0.22	-1.06
MYO5C	55930	-0.24	-0.8	-1.06
C14ORF101	54916	-0.28	-0.65	-1.06
ESYT2	57488	-0.01	-0.56	-1.06
SLC29A4	222962	-0.58	-0.9	-1.06
TIGD5	84948	-0.45	-0.62	-1.06
C12ORF69	440087	-0.51	-0.26	-1.06
LOC143286	143286	-0.15	-0.38	-1.06
LOC339803	339803	-0.33	-0.11	-1.06
KRTAP19-8	728299	0.12	-0.56	-1.06

LOC401022	401022	-0.11	-0.82	-1.06
NCRNA00107	283981	-0.62	-0.52	-1.06
ARPC1B	10095	-0.09	-0.3	-1.05
PRPSAP1	5635	-0.35	-0.56	-1.05
IFIT1	3434	-0.7	-0.84	-1.05
C16ORF62	57020	-0.03	-0.67	-1.05
HMGB3	3149	-0.04	-0.89	-1.05
PALM	5064	-0.05	-0.91	-1.05
MAP2K5	5607	0.15	-0.1	-1.05
NR2F6	2063	0.03	-0.58	-1.05
CBX5	23468	-0.36	-0.76	-1.05
BTN3A2	11118	0.03	-0.39	-1.05
PRICKLE2	166336	-0.57	-0.85	-1.05
STEAP2	261729	-0.26	-0.7	-1.05
F8A2	474383	-0.08	-0.32	-1.05
PAR-SN	347746	-0.3	-0.48	-1.05
LOC100270746	100270746	0.47	0.02	-1.05
SUMF1	285362	0.06	-0.93	-1.05
UFSP1	402682	-0.59	-0.88	-1.05
WDR81	124997	-0.61	-0.51	-1.05
TMED6	146456	-1.05	-0.93	-1.05
ADSL	158	-0.29	-0.67	-1.04
C21ORF33	8209	-0.11	-0.37	-1.04
NAE1	8883	-0.51	-0.87	-1.04
SCO2	9997	-0.22	-0.6	-1.04
ADD2	119	-0.02	-0.37	-1.04
SP110	3431	-0.59	-0.78	-1.04
HSPA5	3309	-0.51	-0.73	-1.04
PEG10	23089	0.26	-0.8	-1.04
TNNT1	7138	-0.11	-0.72	-1.04
C11ORF2	738	0.12	-0.27	-1.04

RSRC1	51319	-0.16	-0.79	-1.04
NLRX1	79671	-0.46	-0.55	-1.04
SLC2A6	11182	-0.36	-0.89	-1.04
C5ORF54	63920	-0.51	-0.66	-1.04
FARSB	10056	-0.32	-0.63	-1.04
SPATA17	128153	-0.21	-0.73	-1.04
C1ORF131	128061	-0.49	-0.42	-1.04
GATS	352954	-0.38	-0.46	-1.04
TERC	7012	-0.27	-0.48	-1.04
DIRC1	116093	0.24	-0.06	-1.04
EEF2	1938	-0.1	-0.35	-1.03
RRBP1	6238	-0.14	-0.48	-1.03
PHB2	11331	-0.06	-0.54	-1.03
PYGL	5836	0.03	-0.56	-1.03
LTK	4058	-0.41	-0.35	-1.03
ZNF189	7743	-0.89	-0.96	-1.03
POLR2I	5438	-0.26	-0.63	-1.03
NTAN1	123803	-0.06	-0.38	-1.03
TRIM66	9866	-0.32	-0.54	-1.03
CDK5RAP3	80279	-0.24	-0.7	-1.03
ANKRD30A	91074	-0.33	-0.62	-1.03
FLJ40504	284085	-0.4	-0.69	-1.03
LOC442249	442249	-0.37	-0.7	-1.03
C5ORF24	134553	-0.26	-0.37	-1.03
CAMK2N2	94032	-0.05	-0.9	-1.03
ZNF70	7621	-0.64	-0.78	-1.03
SEPHS2	22928	-0.12	-0.58	-1.02
DARS	1615	-0.43	-0.78	-1.02
CTSL1	1514	0	-0.18	-1.02
U2AF1	7307	-0.29	-1	-1.02
ICAM3	3385	0.18	0	-1.02

PHTF1	10745	-0.42	-0.88	-1.02
WDR18	57418	-0.24	-0.45	-1.02
NIPAL3	57185	-0.11	-0.37	-1.02
KLRG1	10219	-0.27	-0.6	-1.02
CCDC51	79714	-0.28	-0.42	-1.02
FAM176B	55194	-0.77	-0.72	-1.02
BATF3	55509	-0.32	-1.01	-1.02
AGTRAP	57085	-0.14	-0.67	-1.02
ARRDC1	92714	-0.18	-0.56	-1.02
LOC100130264	100130264	-0.34	-0.91	-1.02
DHFRL1	200895	-0.48	-0.38	-1.02
LOC440104	440104	-0.59	-0.85	-1.02
MTERFD3	80298	-0.7	-0.78	-1.02
KIF3C	3797	0.04	-0.42	-1.01
PRKAR2B	5577	0.2	-0.72	-1.01
CDC7	8317	-0.07	-0.5	-1.01
LGALS2	3957	-0.2	-0.99	-1.01
ALDH7A1	501	-0.02	-0.92	-1.01
KIAA0146	23514	-0.2	-0.81	-1.01
PON3	5446	-0.29	-0.94	-1.01
PGAP2	27315	0.2	-0.59	-1.01
NLK	51701	-0.14	-0.74	-1.01
ARMC7	79637	0.06	-0.28	-1.01
TFB1M	51106	-0.24	-0.14	-1.01
MRPL17	63875	-0.08	-0.37	-1.01
LOC100128077	100128077	-0.49	-0.66	-1.01
LOC100129791	100129791	-0.42	-0.76	-1.01
LOC100130506	100130506	0.06	-0.51	-1.01
OR52B6	340980	-0.32	-0.6	-1.01
OR5L2	26338	0.05	-0.46	-1.01
WBSCR27	155368	-0.2	-0.69	-1.01

ZNF581	51545	-0.28	-0.52	-1.01
BTBD8	284697	-0.33	-0.38	-1.01
LOC653391	653391	-0.51	-0.39	-1.01
PPAPDC2	403313	-0.16	-0.34	-1.01
STK33	65975	-0.33	-0.34	-1.01
PGM2	55276	-0.14	-0.93	-1.01
FNTB	2342	-0.38	-0.9	-1
ANXA8L2	244	-0.25	-0.5	-1
WDR61	80349	-0.1	-0.71	-1
BRCC3	79184	-0.67	-0.98	-1
FZD4	8322	0.01	-0.28	-1
USP18	11274	-0.05	-0.26	-1
FDPSL2A	619190	-0.17	-0.77	-1
