# Supplementary material for: Helveticoside is a biologically active component of the seed extract of Descurainia sophia and induces reciprocal gene regulation in A549 human lung cancer cells
Source: BMC Genomics. 2015 Sep 18;16(1):713. doi: 10.1186/s12864-015-1918-1 (PMC4575430; doi:10.1186/s12864-015-1918-1)
Supplement: Additional file 3: — Top 15 chemicals with high connectivity scores in the analysis of EEDS. (PDF 67 kb) [file 12864_2015_1918_MOESM3_ESM.pdf]

Additional file 3. Top 15 chemicals with high connectivity scores in the analysis of EEDS.

Rank[1]	Name[2]	Mean score[3]	N[4]	Enrichment[5]	p-value[6]	Specificity[7]	% non-null[8]
1	Helveticoside	0.839	6	0.995	0	0	100
2	Lanatoside C	0.850	6	0.995	0	0	100
3	Anisomycin	0.764	4	0.994	0	0.0155	100
4	Digoxigenin	0.765	5	0.993	0	0	100
5	Digoxin	0.753	4	0.993	0	0	100
6	Digitoxigenin	0.726	4	0.991	0	0	100
7	Ouabain	0.737	4	0.989	0	0	100
8	Phenoxybenzamine	0.532	4	0.983	0	0.0594	100
9	Cicloheximide	0.568	4	0.975	0	0.0113	100
10	Puromycin	0.504	4	0.97	0	0.0337	100
11	Withaferin A	0.402	4	0.959	0	0.0316	100
12	Niclosamide	0.411	5	0.938	0	0.0052	100
13	Pyrvinium	0.428	6	0.907	0	0.0093	100
14	Thioridazine	0.360	20	0.708	0	0.0274	95
15	15-delta prostaglandin J2	0.317	15	0.679	0	0.0447	86

[1]Rank is based upon estimates of the likelihood that the enrichment of a set of instances in the ordered list of all instances for that result would be observed by chance.
[2]Name given to a perturbagen.
[3]The arithmetic mean of the connectivity scores for those instances.
[4]The number of those instances.
[5]A measure of the enrichment of those instances in the order list of all instances.
[6]A permutation p-value for that enrichment score.
[7]The specificity of that enrichment.
[8]The non-null percentage.
