# Supplementary material for: Helveticoside is a biologically active component of the seed extract of Descurainia sophia and induces reciprocal gene regulation in A549 human lung cancer cells
Source: BMC Genomics. 2015 Sep 18;16(1):713. doi: 10.1186/s12864-015-1918-1 (PMC4575430; doi:10.1186/s12864-015-1918-1)
Supplement: Additional file 4: — Comparison of gene expression profiles. The gene expression profiles obtained with our EEDS and helveticoside experiments and for the seven top-ranked drugs selected from the Connectivity map database were compared. For the seven top-ranked drugs, the cell lines and drug concentrations used in the experiment are indicated with different colors. (PDF 145 kb) [file 12864_2015_1918_MOESM4_ESM.pdf]

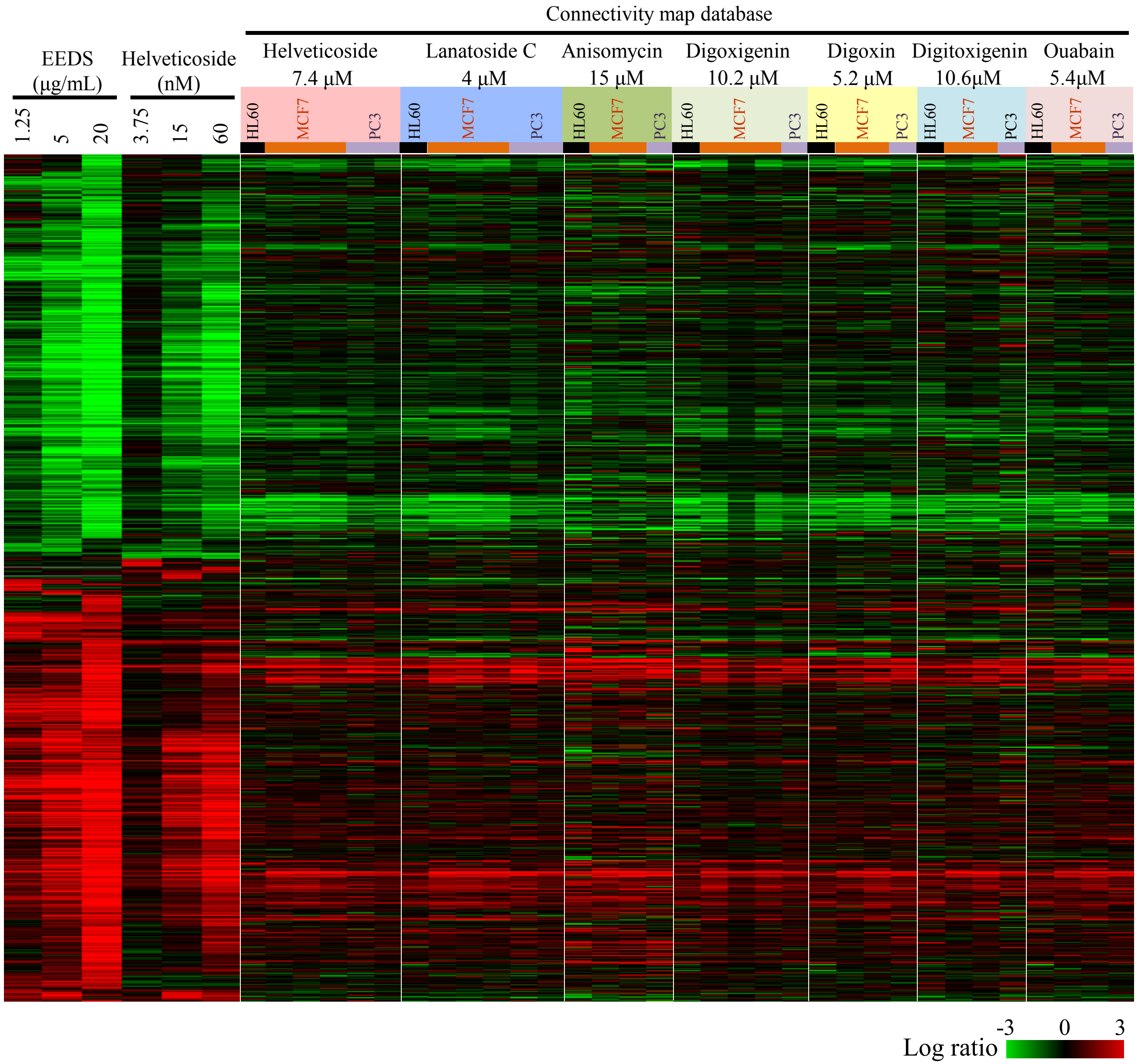

Additional file 4. Comparison of gene expression profiles. The gene expression profiles obtained with our EEDS and helveticoside experiments and for the seven top-ranked drugs selected from the connectivity map database were compared. For the seven top-ranked drugs, the cell line and drug concentration used in the experiment are indicated with different colors.
