# Supplementary material for: Helveticoside is a biologically active component of the seed extract of Descurainia sophia and induces reciprocal gene regulation in A549 human lung cancer cells
Source: BMC Genomics. 2015 Sep 18;16(1):713. doi: 10.1186/s12864-015-1918-1 (PMC4575430; doi:10.1186/s12864-015-1918-1)
Supplement: Additional file 5: — Comparison of the dose-dependently regulated genes after treatment with helveticoside and EEDS. (PDF 53 kb) [file 12864_2015_1918_MOESM5_ESM.pdf]

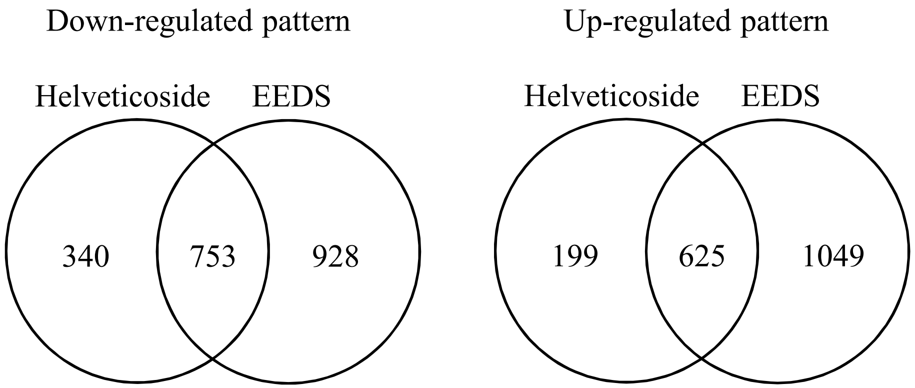

Additional file 5. Comparison of the dose-dependently regulated genes after treatment with helveticoside and EEDS.
