# Supplementary material for: Helveticoside is a biologically active component of the seed extract of Descurainia sophia and induces reciprocal gene regulation in A549 human lung cancer cells
Source: BMC Genomics. 2015 Sep 18;16(1):713. doi: 10.1186/s12864-015-1918-1 (PMC4575430; doi:10.1186/s12864-015-1918-1)
Supplement: Additional file 6: — GO terms enriched (FDR < 0.01) by helveticoside. (PDF 69 kb) [file 12864_2015_1918_MOESM6_ESM.pdf]

Additional file 6. GO terms enriched (FDR<0.01) by helveticoside.

Pattern	GO ID	Name	p-value[1]	FDR[2]
Down-regulated pattern	GO:0055114	Oxidation reduction	4.49E-08	1.13E-04
Up-regulated pattern	GO:0042981	Regulation of apoptosis	9.16E-09	2.56E-05
	GO:0019220	Regulation of phosphate metabolic process	1.29E-08	1.79E-05
	GO:0051174	Regulation of phosphorus metabolic process	1.29E-08	1.79E-05
	GO:0043067	Regulation of programmed cell death	1.41E-08	1.31E-05
	GO:0010941	Regulation of cell death	1.61E-08	1.12E-05
	GO:0006357	Regulation of transcription from RNA polymerase II promoter	5.87E-08	3.28E-05
	GO:0042325	Regulation of phosphorylation	7.44E-08	3.46E-05
	GO:0007167	Enzyme linked receptor protein signaling pathway	1.32E-07	5.26E-05
	GO:0042127	Regulation of cell proliferation	2.59E-07	9.04E-05
	GO:0007242	Intracellular signaling cascade	7.04E-07	2.18E-04
	GO:0045449	Regulation of transcription	7.22E-07	2.01E-04
	GO:0051173	Positive regulation of nitrogen compound metabolic process	1.37E-06	3.48E-04
	GO:0009891	Positive regulation of biosynthetic process	1.48E-06	3.45E-04
	GO:0009890	Negative regulation of biosynthetic process	2.52E-06	5.41E-04
	GO:0031327	Negative regulation of cellular biosynthetic process	3.08E-06	6.13E-04
	GO:0001568	Blood vessel development	3.22E-06	5.99E-04
	GO:0016265	Cell death	4.14E-06	7.21E-04
	GO:0031328	Positive regulation of cellular biosynthetic process	4.27E-06	7.02E-04
	GO:0012501	Programmed cell death	5.11E-06	7.92E-04
	GO:0006350	Transcription	5.14E-06	7.55E-04
	GO:0001944	Vasculature development	5.18E-06	7.22E-04
	GO:0043066	Negative regulation of apoptosis	5.43E-06	7.22E-04
	GO:0045935	Positive regulation of nucleobase, nucleoside, nucleotide and nucleic acid metabolic process	5.61E-06	7.11E-04
	GO:0008219	Cell death	6.85E-06	8.31E-04
	GO:0043069	Negative regulation of programmed cell death	7.43E-06	8.64E-04
	GO:0051338	Regulation of transferase activity	7.44E-06	8.30E-04
	GO:0060548	Negative regulation of cell death	7.89E-06	8.47E-04
	GO:0045859	Regulation of protein kinase activity	8.96E-06	9.26E-04
	GO:0010557	Positive regulation of macromolecule biosynthetic process	9.83E-06	9.79E-04

GO:0010558	Negative regulation of macromolecule biosynthetic process	1.65E-05	1.58E-03
GO:0048514	Blood vessel morphogenesis	1.73E-05	1.61E-03
GO:0043549	Regulation of kinase activity	1.85E-05	1.66E-03
GO:0051252	Regulation of RNA metabolic process	2.85E-05	2.48E-03
GO:0010604	Positive regulation of macromolecule metabolic process	3.22E-05	2.71E-03
GO:0045934	Negative regulation of nucleobase, nucleoside, nucleotide and nucleic acid metabolic process	3.23E-05	2.64E-03
GO:0006915	Apoptosis	3.27E-05	2.60E-03
GO:0007243	Protein kinase cascade	3.65E-05	2.82E-03
GO:0051172	Negative regulation of nitrogen compound metabolic process	4.46E-05	3.35E-03
GO:0007169	Transmembrane receptor protein tyrosine kinase signaling pathway	4.99E-05	3.66E-03
GO:0008285	Negative regulation of cell proliferation	5.56E-05	3.97E-03
GO:0016481	Negative regulation of transcription	5.85E-05	4.07E-03
GO:0051254	Positive regulation of RNA metabolic process	6.88E-05	4.67E-03
GO:0043065	Positive regulation of apoptosis	6.91E-05	4.58E-03
GO:0006355	Regulation of transcription, DNA-dependent	7.28E-05	4.71E-03
GO:0043068	Positive regulation of programmed cell death	8.02E-05	5.07E-03
GO:0010942	Positive regulation of cell death	8.73E-05	5.39E-03
GO:0045892	Negative regulation of transcription, DNA-dependent	9.42E-05	5.70E-03
GO:0060348	Bone development	9.45E-05	0.005594
GO:0010629	Negative regulation of gene expression	9.94E-05	0.005761
GO:0045893	Positive regulation of transcription, DNA-dependent	1.21E-04	0.00687
GO:0045860	Positive regulation of protein kinase activity	1.27E-04	0.007088
GO:0051253	Negative regulation of RNA metabolic process	1.28E-04	0.006991
GO:0051726	Regulation of cell cycle	1.33E-04	0.0071
GO:0010628	Positive regulation of gene expression	1.35E-04	0.007072
GO:0045941	Positive regulation of transcription	1.35E-04	0.006952
GO:0051347	Positive regulation of transferase activity	1.48E-04	0.007476
GO:0001503	Ossification	1.50E-04	0.007469
GO:0032570	Response to progesterone stimulus	1.60E-04	0.007822
GO:0045944	Positive regulation of transcription from RNA polymerase II promoter	1.91E-04	0.009167

[1]p-values were calculated using the Fischer's test.

[2]FDR corrections were calculated using the Benjamini-Hochberg procedure.
