# Supplementary material for: Helveticoside is a biologically active component of the seed extract of Descurainia sophia and induces reciprocal gene regulation in A549 human lung cancer cells
Source: BMC Genomics. 2015 Sep 18;16(1):713. doi: 10.1186/s12864-015-1918-1 (PMC4575430; doi:10.1186/s12864-015-1918-1)
Supplement: Additional file 7: — Enriched GO terms in the up-regulated pattern after treatment of A549 cells with helveticoside. (A) The network structure was constructed with non-redundant GO terms that were enriched in the up-regulated pattern (FDR < 0.01) after helveticoside treatment using the REIVGO program. The node color indicates the statistical significance, and the node size is proportional to the frequency of the GO term in the underlying GOA database. Highly correlated GO terms are linked by edges, the thickness of which indicates the degree of similarity. (B) Tree map structures composed of non-redundant GO terms (FDR < 0.01) enriched in the up-regulated pattern after helveticoside treatment were constructed. In the tree structures, closely related terms share the same color. The size of each GO term is proportional to its level of statistical significance. (PDF 88 kb) [file 12864_2015_1918_MOESM7_ESM.pdf]

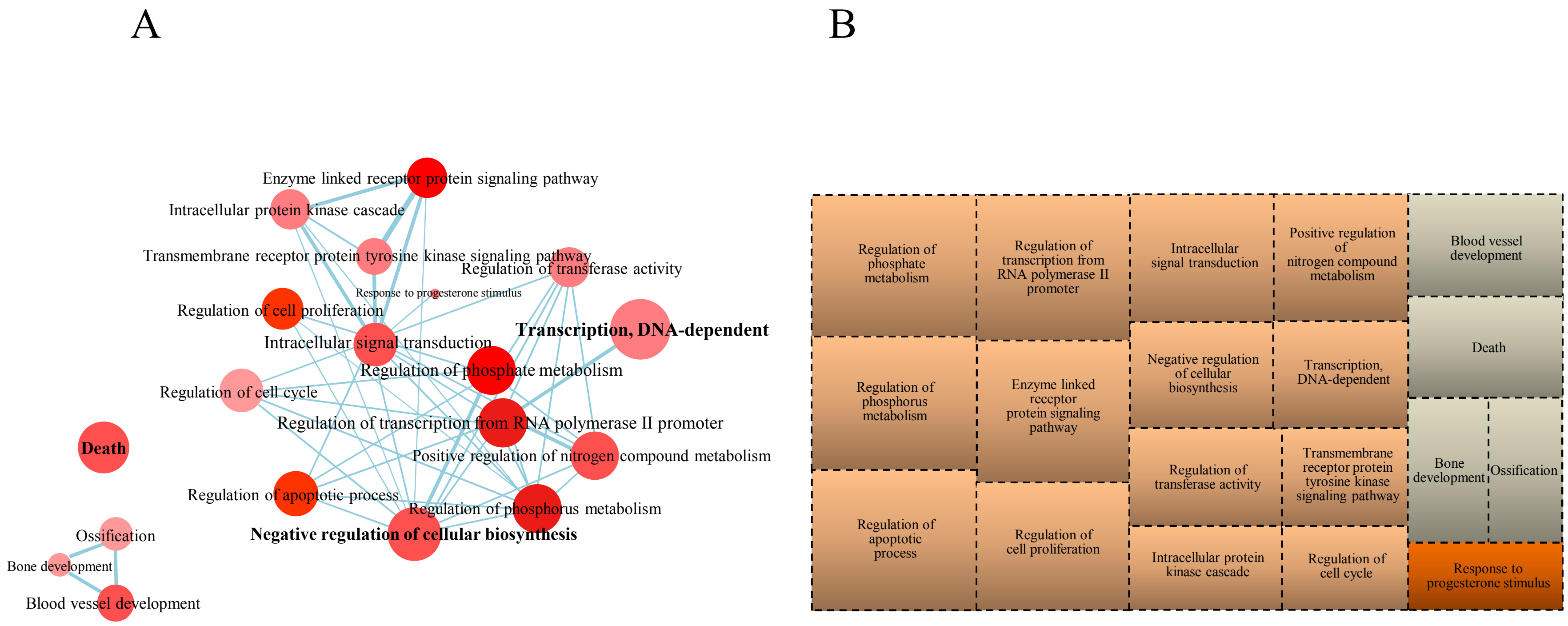

Additional file 7. Enriched GO terms in the up-regulated pattern after treatment of A549 cells with helveticoside. (A) The network of the non-redundant GO terms that were enriched in the up-regulated pattern (FDR<0.01) after helveticoside treatment was constructed using the REIVGO program. The node color indicates the statistical significance, and the node size is proportional to the frequency of the GO term in the underlying GOA database. Highly correlated GO terms are linked by edges, the thickness of which indicates the degree of similarity. (B) Tree map structures composed of non-redundant GO terms (FDR<0.01) enriched in the up-regulated pattern after helveticoside treatment were constructed. In the tree structures, closely related terms share the same color. The size of each GO term is proportional to its level of statistical significance.
