# Supplementary material for: Helveticoside is a biologically active component of the seed extract of Descurainia sophia and induces reciprocal gene regulation in A549 human lung cancer cells
Source: BMC Genomics. 2015 Sep 18;16(1):713. doi: 10.1186/s12864-015-1918-1 (PMC4575430; doi:10.1186/s12864-015-1918-1)
Supplement: Additional file 8: — Enriched GO terms in the down-regulated pattern after treatment of A549 cells with helveticoside. The network structure was constructed from the non-redundant GO terms enriched in the down-regulated pattern after helveticoside treatment using the REIVGO program. We lowered the input stringency threshold to p-value < 0.01 to increase the number of GO input terms. The node size and color intensity are proportional to the hierarchical status and statistical significance of each node, respectively. The edge thickness between nodes represents the closeness of the two nodes. In the tree structure, closely related terms share the same color. The size of each GO term is proportional to its level of statistical significance. (PDF 89 kb) [file 12864_2015_1918_MOESM8_ESM.pdf]

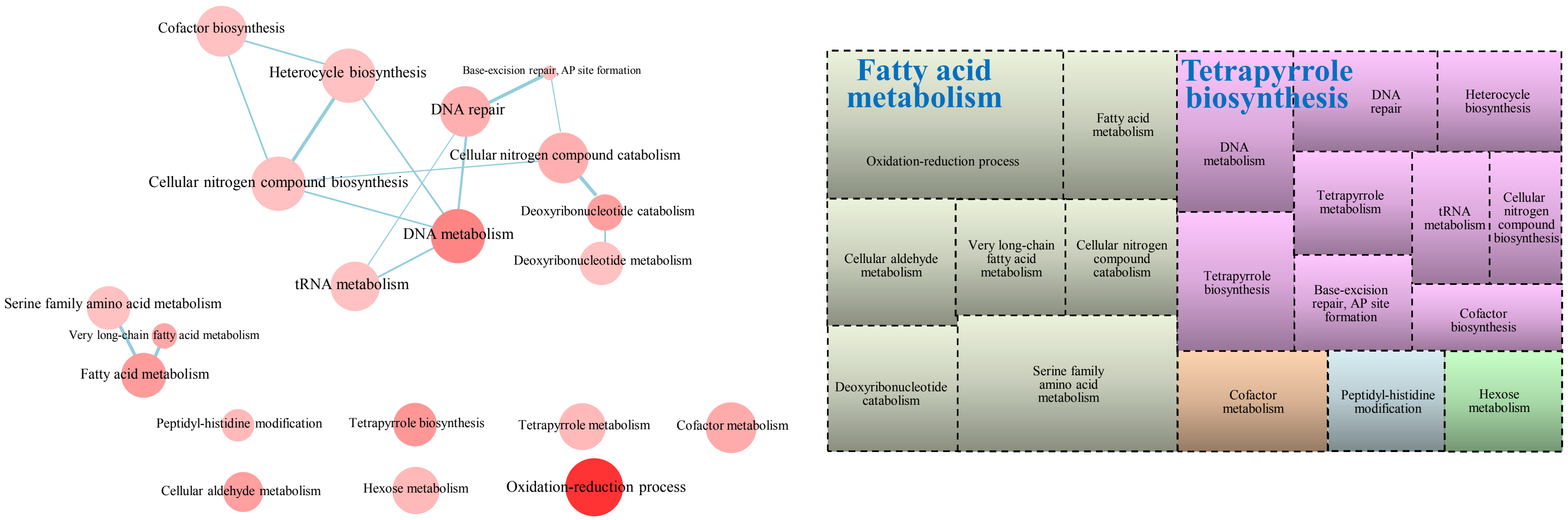

Additional file 8. Enriched GO terms in the down-regulated pattern after treatment of A549 cells with helveticoside. The network of the non-redundant GO terms enriched in the down-regulated pattern after helveticoside treatment was constructed using the REIVGO program. We lowered the input stringency threshold to a p-value of less than 0.01 to increase the number of GO input terms. The node size and color intensity are proportional to the hierarchical status and statistical significance of each node, respectively. The thickness of the edges between nodes represents the closeness of the two nodes. In the tree structure, closely related terms share the same color. The size of each GO term is proportional to its level of statistical significance.
