# Supplementary material for: Helveticoside is a biologically active component of the seed extract of Descurainia sophia and induces reciprocal gene regulation in A549 human lung cancer cells
Source: BMC Genomics. 2015 Sep 18;16(1):713. doi: 10.1186/s12864-015-1918-1 (PMC4575430; doi:10.1186/s12864-015-1918-1)
Supplement: Additional file 9: — Distribution of enriched GO terms (FDR < 0.01) across all of the samples, including our EEDS and helveticoside experiments and the 15 top-ranked drugs from the Connectivity map database. (PDF 86 kb) [file 12864_2015_1918_MOESM9_ESM.pdf]

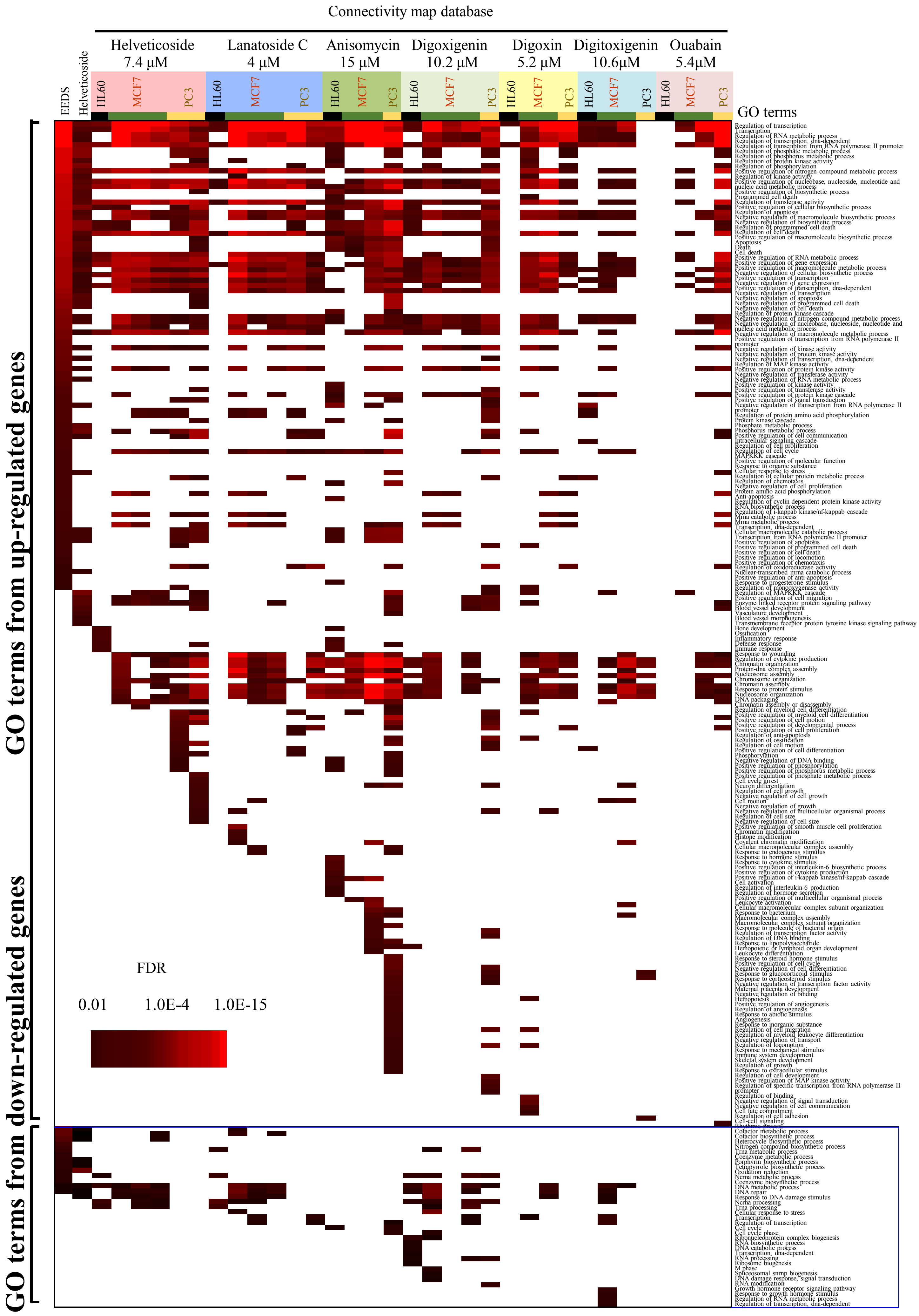

Additional file 9. Distribution of enriched GO terms (FDR<0.01) across all of the samples, including our EEDS and helveticoside experiments and the 15 top-ranked drugs from the Connectivity map database
