# Supplementary figures and images for: Helveticoside is a biologically active component of the seed extract of Descurainia sophia and induces reciprocal gene regulation in A549 human lung cancer cells
Source: BMC Genomics. 2015 Sep 18;16(1):713. doi: 10.1186/s12864-015-1918-1 (PMC4575430; doi:10.1186/s12864-015-1918-1)

Connectivity map database

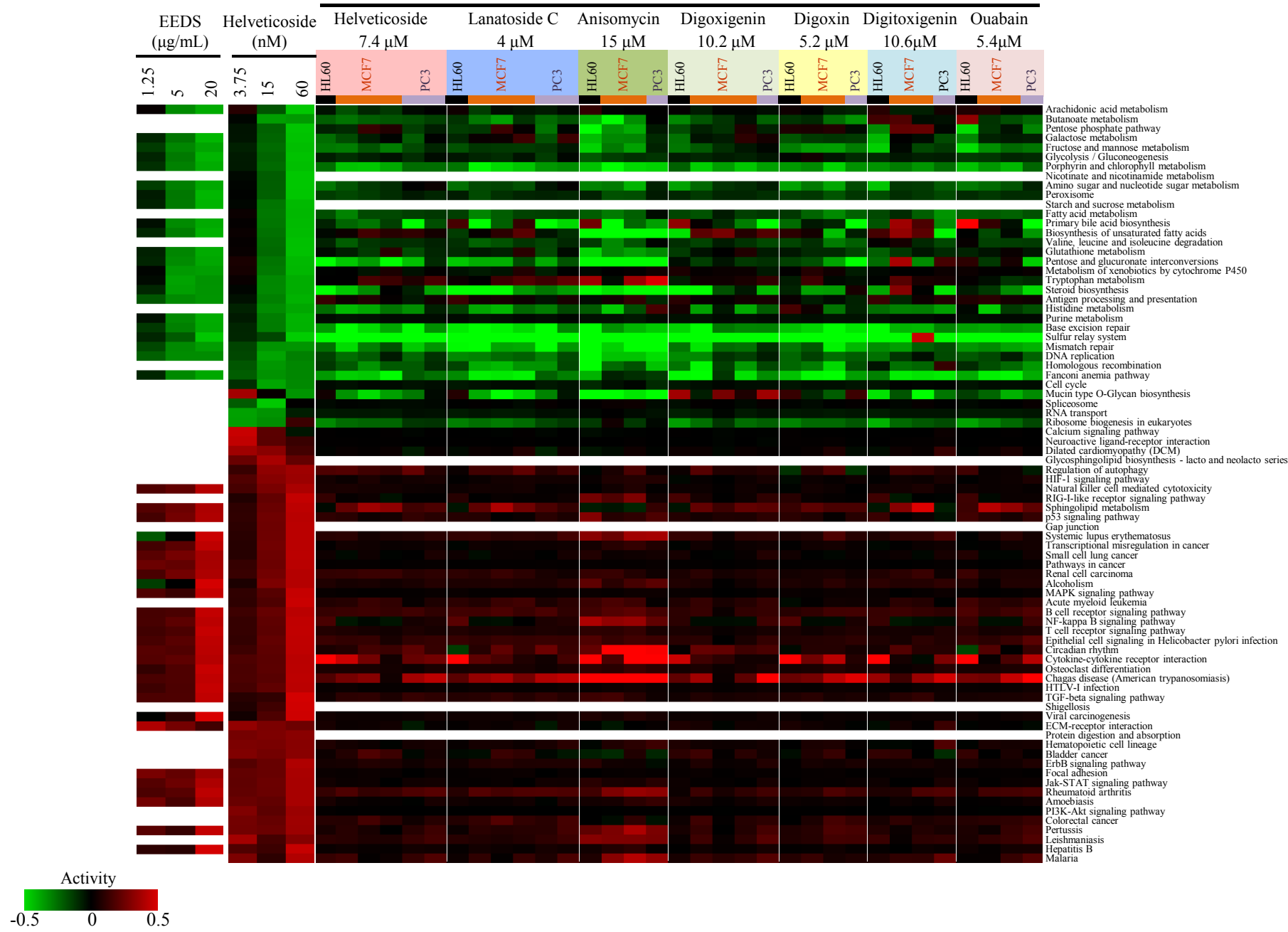

Supplement: Additional file 10: — Dose-dependent changes in pathway activity. (PDF 105 kb) [file 12864_2015_1918_MOESM10_ESM.pdf]
