# Supplementary material for: Helveticoside is a biologically active component of the seed extract of Descurainia sophia and induces reciprocal gene regulation in A549 human lung cancer cells
Source: BMC Genomics. 2015 Sep 18;16(1):713. doi: 10.1186/s12864-015-1918-1 (PMC4575430; doi:10.1186/s12864-015-1918-1)
Supplement: Additional file 11: — Full list of significantly enriched GO terms determined through Network Ontology Analysis (NOA). (PDF 77 kb) [file 12864_2015_1918_MOESM11_ESM.pdf]

Additional file 11. Full list of significantly enriched GO terms determined through network ontology analysis (NOA).

GO term		p-value	Corrected p-value
GO:0006396	RNA processing	1.90E-31	7.00E-29
GO:0008380	RNA splicing	1.90E-31	7.00E-29
GO:0006464	Protein modification process	3.60E-21	1.30E-18
GO:0019538	Protein metabolic process	9.90E-21	3.50E-18
GO:0016070	RNA metabolic process	2.20E-19	8.10E-17
GO:0044267	Cellular protein metabolic process	2.40E-19	8.60E-17
GO:0043170	Macromolecule metabolic process	7.20E-16	2.50E-13
GO:0044260	Cellular macromolecule metabolic process	5.40E-15	1.90E-12
GO:0043412	Macromolecule modification	9.10E-15	3.20E-12
GO:0010628	Positive regulation of gene expression	2.20E-14	8.00E-12
GO:0043687	Post-translational protein modification	5.60E-14	2.00E-11
GO:0051252	Regulation of RNA metabolic process	4.70E-13	1.60E-10
GO:0006355	Regulation of transcription, DNA-dependent	4.70E-13	1.60E-10
GO:0006354	RNA elongation	8.40E-13	3.00E-10
GO:0006368	RNA elongation from RNA polymerase II promoter	8.40E-13	3.00E-10
GO:0090304	Nucleic acid metabolic process	1.60E-12	5.80E-10
GO:0010604	Positive regulation of macromolecule metabolic process	2.70E-12	9.90E-10
GO:0044238	Primary metabolic process	2.80E-12	1.00E-09
GO:0045935	Positive regulation of nucleobase, nucleoside, nucleotide and nucleic acid metabolic process	3.30E-12	1.20E-09
GO:0051173	Positive regulation of nitrogen compound metabolic process	3.30E-12	1.20E-09
GO:0016567	Protein ubiquitination	7.70E-12	2.70E-09
GO:0032446	Protein modification by small protein conjugation	7.70E-12	2.70E-09
GO:0070647	Protein modification by small protein conjugation or removal	7.70E-12	2.70E-09
GO:0010557	Positive regulation of macromolecule biosynthetic process	8.60E-12	3.00E-09
GO:0044237	Cellular metabolic process	1.10E-11	4.20E-09
GO:0006139	Nucleobase, nucleoside, nucleotide and nucleic acid metabolic process	2.00E-11	7.50E-09
GO:0008152	Metabolic process	2.80E-11	1.00E-08
GO:0009893	Positive regulation of metabolic process	3.50E-11	1.20E-08
GO:0031328	Positive regulation of cellular biosynthetic process	5.00E-11	1.70E-08
GO:0034641	Cellular nitrogen compound metabolic process	5.50E-11	1.90E-08
GO:0006807	Nitrogen compound metabolic process	5.50E-11	1.90E-08
GO:0009891	Positive regulation of biosynthetic process	1.10E-10	4.10E-08
GO:0006357	Regulation of transcription from RNA polymerase II promoter	1.60E-10	5.90E-08
GO:0051246	Regulation of protein metabolic process	4.60E-10	1.60E-07
GO:0031325	Positive regulation of cellular metabolic process	5.80E-10	2.00E-07
GO:0016071	mRNA metabolic process	2.00E-09	7.30E-07
GO:0006367	Transcription initiation from RNA polymerase II promoter	2.00E-09	7.30E-07
GO:0006397	mRNA processing	2.00E-09	7.30E-07
GO:0045595	Regulation of cell differentiation	7.40E-09	2.60E-06

GO term		p-value	corrected p-value
GO:0060255	Regulation of macromolecule metabolic process	7.70E-09	2.70E-06
GO:0006352	Transcription initiation	1.30E-08	4.60E-06
GO:0031324	Negative regulation of cellular metabolic process	1.70E-08	6.10E-06
GO:0045893	Positive regulation of transcription, DNA-dependent	1.70E-08	6.10E-06
GO:0051254	Positive regulation of RNA metabolic process	1.70E-08	6.10E-06
GO:0051603	Proteolysis involved in cellular protein catabolic process	1.70E-08	6.30E-06
GO:0006508	Proteolysis	1.70E-08	6.30E-06
GO:0019219	Regulation of nucleobase, nucleoside, nucleotide and nucleic acid metabolic process	2.50E-08	9.00E-06
GO:0051171	Regulation of nitrogen compound metabolic process	2.50E-08	9.00E-06
GO:0010468	Regulation of gene expression	2.50E-08	9.10E-06
GO:0080090	Regulation of primary metabolic process	2.60E-08	9.60E-06
GO:0048523	Negative regulation of cellular process	3.40E-08	1.20E-05
GO:0048519	Negative regulation of biological process	4.70E-08	1.70E-05
GO:0010556	Regulation of macromolecule biosynthetic process	6.40E-08	2.30E-05
GO:0050896	Response to stimulus	9.00E-08	3.20E-05
GO:0010605	Negative regulation of macromolecule metabolic process	1.50E-07	5.50E-05
GO:0009892	Negative regulation of metabolic process	1.50E-07	5.50E-05
GO:0050793	Regulation of developmental process	2.20E-07	7.90E-05
GO:0045596	Negative regulation of cell differentiation	3.30E-07	1.10E-04
GO:0007219	Notch signaling pathway	3.30E-07	1.20E-04
GO:0006954	Inflammatory response	3.60E-07	1.20E-04
GO:0019941	Modification-dependent protein catabolic process	3.80E-07	1.30E-04
GO:0043632	Modification-dependent macromolecule catabolic process	3.80E-07	1.30E-04
GO:0006511	Ubiquitin-dependent protein catabolic process	3.80E-07	1.30E-04
GO:0048518	Positive regulation of biological process	3.80E-07	1.30E-04
GO:0048869	Cellular developmental process	5.10E-07	1.80E-04
GO:0019222	Regulation of metabolic process	8.10E-07	2.90E-04
GO:0031326	Regulation of cellular biosynthetic process	8.30E-07	2.90E-04
GO:0006950	Response to stress	8.70E-07	3.10E-04
GO:0048522	Positive regulation of cellular process	1.20E-06	4.40E-04
GO:0009889	Regulation of biosynthetic process	1.80E-06	6.70E-04
GO:0031323	Regulation of cellular metabolic process	2.50E-06	8.90E-04
