# Supplementary material for: Helveticoside is a biologically active component of the seed extract of Descurainia sophia and induces reciprocal gene regulation in A549 human lung cancer cells
Source: BMC Genomics. 2015 Sep 18;16(1):713. doi: 10.1186/s12864-015-1918-1 (PMC4575430; doi:10.1186/s12864-015-1918-1)
Supplement: Additional file 12: — Tree map structure of the GO terms enriched in each module of Fig. 7c . Closely related GO terms share the same color. The size of each GO term is proportional to its level of statistical significance. (PDF 143 kb) [file 12864_2015_1918_MOESM12_ESM.pdf]

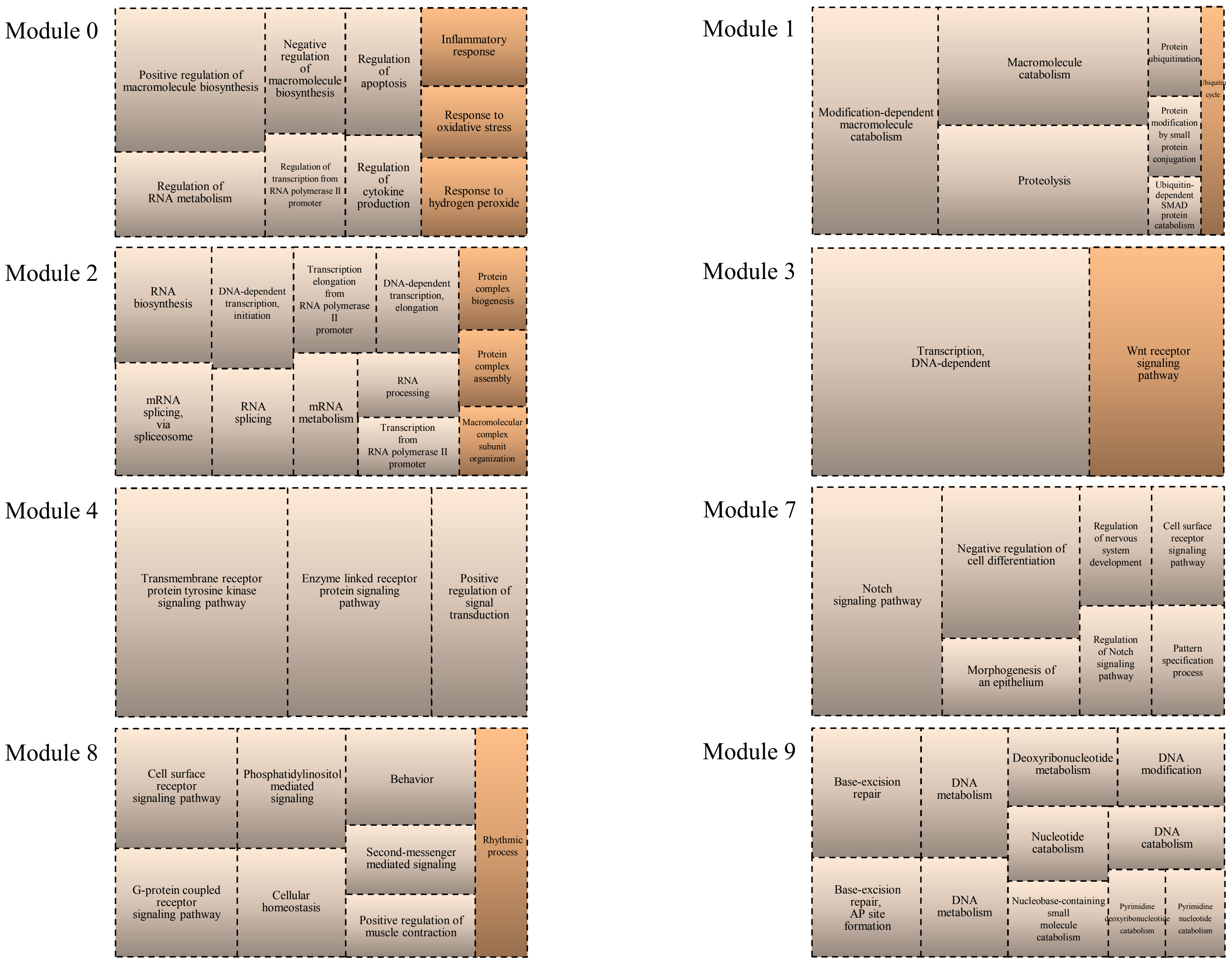

Additional file 12. Tree map structure of the GO terms enriched in each module presented in Figure 7C. Closely related GO terms share the same color. The size of each GO term is proportional to its level of statistical significance.
