# Supplementary material for: Helveticoside is a biologically active component of the seed extract of Descurainia sophia and induces reciprocal gene regulation in A549 human lung cancer cells
Source: BMC Genomics. 2015 Sep 18;16(1):713. doi: 10.1186/s12864-015-1918-1 (PMC4575430; doi:10.1186/s12864-015-1918-1)
Supplement: Additional file 13: — Genes included in each module of network structure. (PDF 121 kb) [file 12864_2015_1918_MOESM13_ESM.pdf]

Additional file 13. Genes included in each module of the network structure.

Module[1]	Name	Degree	Betweenness centrality	Closeness centrality	Clustering coefficient
0	AFAP1L2	3	0.000831	0.341	0.666
0	AKR1B1	3	0.000831	0.283	0.666
0	ANKRD1	2	0	0.332	1
0	AREG	3	0.000831	0.283	0.666
0	BATF3	1	0	0.322	0
0	CITED4	1	0	0.264	0
0	CXCL1	5	0.00355	0.338	0.8
0	CXCL2	3	0	0.277	1
0	DAK	1	0	0.322	0
0	EGR1	15	0.115	0.358	0.0761
0	FCER2	1	0	0.264	0
0	FOXF2	1	0	0.264	0
0	HOXA7	1	0	0.322	0
0	IER3	1	0	0.269	0
0	IMPDH2	1	0	0.264	0
0	JUN	26	0.735	0.474	0.0615
0	MAP3K14	3	0	0.277	1
0	NAT1	1	0	0.322	0
0	NFKB1	18	0.106	0.367	0.163
0	NFKBIB	4	0	0.277	1
0	NFKBIE	4	0	0.277	1
0	NNMT	1	0	0.322	0
0	PHB2	1	0	0.267	0
0	PPP1R15B	1	0	0.322	0
0	REL	8	0.00847	0.348	0.607
0	RELA	16	0.0727	0.364	0.233
0	RELB	7	0.00903	0.340	0.666
0	RRM2B	1	0	0.269	0
0	SERPINB9	1	0	0.264	0
0	SS18	1	0	0.322	0
0	TBX2	2	0	0.332	1
0	TGM2	1	0	0.267	0
0	TM4SF1	1	0	0.322	0
0	TNFRSF10A	2	0	0.331	1
0	TNS4	1	0	0.269	0
1	ASB10	15	0.000146	0.241	0.904
1	ASB9	15	0.000146	0.241	0.904

1	DET1	15	0.000146	0.241	0.904
1	DZIP3	15	0.000146	0.241	0.904
1	FBXO4	15	0.000146	0.241	0.904
1	KLHL21	15	0.000146	0.241	0.904
1	RNF111	15	0.000146	0.241	0.904
1	RNF41	15	0.000146	0.241	0.904
1	SMURF1	15	0.000146	0.241	0.904
1	SMURF2	16	0.234	0.305	0.791
1	SPSB2	15	0.000146	0.241	0.904
1	UBE2D4	11	0	0.239	1
1	UBE2Q2	11	0	0.239	1
1	UBE2R2	11	0	0.239	1
1	UBE2S	11	0	0.239	1
1	UBE2W	11	0	0.239	1
2	BRF2	2	0	0.214	1
2	CPSF7	6	0	0.215	1
2	ELL	3	0	0.214	1
2	GTF3A	3	0.193	0.346	0.333
2	PCF11	6	0	0.215	1
2	PHF5A	6	0	0.215	1
2	POLR2D	8	0.000483	0.216	0.678
2	POLR2I	8	0.000483	0.216	0.678
2	POLR2K	12	0.159	0.271	0.318
2	POLR3GL	3	0.00812	0.265	0.666
2	PTBP1	6	0	0.215	1
2	PTRF	1	0	0.213	0
2	TAF13	3	0	0.214	1
3	ADD2	1	0	0.282	0
3	ARID1A	2	0	0.351	1
3	ATOH8	1	0	0.282	0
3	BHLHE40	2	0.00949	0.302	0
3	FNIP1	1	0	0.282	0
3	HOXB13	1	0	0.282	0
3	IRAK1BP1	1	0	0.282	0
3	MC1R	1	0	0.282	0
3	NR4A2	2	0.0130	0.309	0
3	TCF7L2	14	0.3780	0.391	0.0219
3	TLE3	2	0	0.282	1
3	TLE4	2	0	0.282	1
4	CCDC6	1	0	0.252	0

4	EML4	1	0	0.252	0
4	EREG	2	0	0.252	1
4	GRB14	1	0	0.252	0
4	HBEGF	2	0	0.252	1
4	NRAS	8	0.121	0.336	0.0357
4	PTPRA	1	0	0.252	0
4	SHOC2	1	0	0.252	0
5	CDH1	7	0.0875	0.287	0.0952
5	CRYAB	1	0	0.223	0
5	CTNNAL1	2	0	0.224	1
5	KLF4	4	0.0474	0.318	0.166
5	KLF6	4	0.0698	0.347	0.333
5	KLRG1	1	0	0.223	0
5	LIMA1	1	0	0.223	0
5	PCDH1	2	0	0.224	1
6	APC2	2	0	0.259	1
6	ARPP19	1	0	0.259	0
6	MIS12	3	0	0.260	1
6	PPP2CB	9	0.104	0.348	0.194
6	RANGAP1	3	0	0.260	1
6	SKA2	3	0	0.260	1
6	TBL1Y	2	0	0.259	1
7	DLL3	5	0.0115	0.255	0.7
7	JAG1	5	0.0115	0.255	0.7
7	MIB1	3	0	0.204	1
7	NEURL	3	0	0.204	1
7	NOTCH2	6	0.0293	0.255	0.466
7	NOTCH3	4	0.102	0.334	0.5
7	PLK3	1	0	0.204	0
8	EDN2	1	0	0.204	0
8	GNAQ	6	0.0876	0.256	0
8	NMS	1	0	0.204	0
8	OXTR	2	0.102	0.334	0
8	P2RY6	1	0	0.204	0
8	RGS2	1	0	0.204	0
8	SLC9A3R1	1	0	0.204	0
9	APEX1	6	0.933	1	0.0666
9	LIG1	1	0	0.545	0
9	MBD4	1	0	0.545	0
9	MUTYH	1	0	0.545	0

9	NTHL1	1	0	0.545	0
10	MPG	2	0	0.6	1
10	XRCC1	2	0	0.6	1
0	AFAP1L2	3	0.000831	0.341	0.666
0	AKR1B1	3	0.000831	0.283	0.666
0	ANKRD1	2	0	0.332	1
0	AREG	3	0.000831	0.283	0.666
0	BATF3	1	0	0.322	0
0	CITED4	1	0	0.264	0
0	CXCL1	5	0.00355	0.338	0.8
0	CXCL2	3	0	0.277	1
0	DAK	1	0	0.322	0
0	EGR1	15	0.115	0.358	0.0761
0	FCER2	1	0	0.264	0
0	FOXF2	1	0	0.264	0
0	HOXA7	1	0	0.322	0
0	IER3	1	0	0.269	0
0	IMPDH2	1	0	0.264	0
0	JUN	26	0.735	0.474	0.0615
0	MAP3K14	3	0	0.277	1
0	NAT1	1	0	0.322	0
0	NFKB1	18	0.106	0.367	0.163
0	NFKBIB	4	0	0.277	1
0	NFKBIE	4	0	0.277	1
0	NNMT	1	0	0.322	0
0	PHB2	1	0	0.267	0
0	PPP1R15B	1	0	0.322	0
0	REL	8	0.00847	0.348	0.607
0	RELA	16	0.0727	0.364	0.233
0	RELB	7	0.00903	0.340	0.666
0	RRM2B	1	0	0.269	0
0	SERPINB9	1	0	0.264	0
0	SS18	1	0	0.322	0
0	TBX2	2	0	0.332	1
0	TGM2	1	0	0.267	0
0	TM4SF1	1	0	0.322	0
0	TNFRSF10A	2	0	0.331	1
0	TNS4	1	0	0.269	0
1	ASB10	15	0.000146	0.241	0.904
1	ASB9	15	0.000146	0.241	0.904

1	DET1	15	0.000146	0.241	0.904
1	DZIP3	15	0.000146	0.241	0.904
1	FBXO4	15	0.000146	0.241	0.904
1	KLHL21	15	0.000146	0.241	0.904
1	RNF111	15	0.000146	0.241	0.904
1	RNF41	15	0.000146	0.241	0.904
1	SMURF1	15	0.000146	0.241	0.904
1	SMURF2	16	0.234	0.305	0.791
1	SPSB2	15	0.000146	0.241	0.904
1	UBE2D4	11	0	0.239	1
1	UBE2Q2	11	0	0.239	1
1	UBE2R2	11	0	0.239	1
1	UBE2S	11	0	0.239	1
1	UBE2W	11	0	0.239	1
2	BRF2	2	0	0.214	1
2	CPSF7	6	0	0.215	1
2	ELL	3	0	0.214	1
2	GTF3A	3	0.193	0.346	0.333
2	PCF11	6	0	0.215	1
2	PHF5A	6	0	0.215	1
2	POLR2D	8	0.000483	0.216	0.678
2	POLR2I	8	0.000483	0.216	0.678
2	POLR2K	12	0.159	0.271	0.318
2	POLR3GL	3	0.00812	0.265	0.666
2	PTBP1	6	0	0.215	1
2	PTRF	1	0	0.213	0
2	TAF13	3	0	0.214	1
3	ADD2	1	0	0.282	0
3	ARID1A	2	0	0.351	1
3	ATOH8	1	0	0.282	0
3	BHLHE40	2	0.00949	0.302	0
3	FNIP1	1	0	0.282	0
3	HOXB13	1	0	0.282	0
3	IRAK1BP1	1	0	0.282	0
3	MC1R	1	0	0.282	0
3	NR4A2	2	0.0130	0.309	0
3	TCF7L2	14	0.378	0.391	0.0219
3	TLE3	2	0	0.282	1
3	TLE4	2	0	0.282	1
4	CCDC6	1	0	0.252	0

4	EML4	1	0	0.252	0
4	EREG	2	0	0.252	1
4	GRB14	1	0	0.252	0
4	HBEGF	2	0	0.252	1
4	NRAS	8	0.121	0.336	0.0357
4	PTPRA	1	0	0.252	0
4	SHOC2	1	0	0.252	0
5	CDH1	7	0.0875	0.287	0.0952
5	CRYAB	1	0	0.223	0
5	CTNNAL1	2	0	0.224	1
5	KLF4	4	0.0474	0.318	0.166
5	KLF6	4	0.0698	0.347	0.333
5	KLRG1	1	0	0.223	0
5	LIMA1	1	0	0.223	0
5	PCDH1	2	0	0.224	1
6	APC2	2	0	0.259	1
6	ARPP19	1	0	0.259	0
6	MIS12	3	0	0.260	1
6	PPP2CB	9	0.104	0.348	0.194
6	RANGAP1	3	0	0.260	1
6	SKA2	3	0	0.260	1
6	TBL1Y	2	0	0.259	1
7	DLL3	5	0.0115	0.255	0.7
7	JAG1	5	0.0115	0.255	0.7
7	MIB1	3	0	0.204	1
7	NEURL	3	0	0.204	1
7	NOTCH2	6	0.0293	0.255	0.466
7	NOTCH3	4	0.102	0.334	0.5
7	PLK3	1	0	0.204	0
8	EDN2	1	0	0.204	0
8	GNAQ	6	0.087	0.256	0
8	NMS	1	0	0.204	0
8	OXTR	2	0.102	0.334	0
8	P2RY6	1	0	0.204	0
8	RGS2	1	0	0.204	0
8	SLC9A3R1	1	0	0.204	0
9	APEX1	6	0.933	1	0.0666
9	LIG1	1	0	0.545	0
9	MBD4	1	0	0.545	0
9	MUTYH	1	0	0.545	0

9	NTHL1	1	0	0.545	0
10	MPG	2	0	0.6	1
10	XRCC1	2	0	0.6	1
0	AFAP1L2	3	0.000831	0.341	0.666
0	AKR1B1	3	0.000831	0.283	0.666
0	ANKRD1	2	0	0.332	1
0	AREG	3	0.000831	0.283	0.666
0	BATF3	1	0	0.322	0
0	CITED4	1	0	0.264	0
0	CXCL1	5	0.00355	0.338	0.8
0	CXCL2	3	0	0.277	1
0	DAK	1	0	0.322	0
0	EGR1	15	0.115	0.358	0.0761
0	FCER2	1	0	0.264	0
0	FOXF2	1	0	0.264	0
0	HOXA7	1	0	0.322	0
0	IER3	1	0	0.269	0
0	IMPDH2	1	0	0.264	0
0	JUN	26	0.735	0.474	0.0615
0	MAP3K14	3	0	0.277	1
0	NAT1	1	0	0.322	0
0	NFKB1	18	0.106	0.367	0.163
0	NFKBIB	4	0	0.277	1
0	NFKBIE	4	0	0.277	1
0	NNMT	1	0	0.322	0
0	PHB2	1	0	0.267	0
0	PPP1R15B	1	0	0.322	0
0	REL	8	0.00847	0.348	0.607
0	RELA	16	0.0727	0.364	0.233
0	RELB	7	0.00903	0.340	0.666
0	RRM2B	1	0	0.269	0
0	SERPINB9	1	0	0.264	0
0	SS18	1	0	0.322	0
0	TBX2	2	0	0.332	1
0	TGM2	1	0	0.267	0
0	TM4SF1	1	0	0.322	0
0	TNFRSF10A	2	0	0.331	1
0	TNS4	1	0	0.269	0
1	ASB10	15	0.000146	0.241	0.904
1	ASB9	15	0.000146	0.241	0.904

1	DET1	15	0.000146	0.241	0.904
1	DZIP3	15	0.000146	0.241	0.904
1	FBXO4	15	0.000146	0.241	0.904
1	KLHL21	15	0.000146	0.241	0.904
1	RNF111	15	0.000146	0.241	0.904
1	RNF41	15	0.000146	0.241	0.904
1	SMURF1	15	0.000146	0.241	0.904
1	SMURF2	16	0.234	0.305	0.791
1	SPSB2	15	0.000146	0.241	0.904
1	UBE2D4	11	0	0.239	1
1	UBE2Q2	11	0	0.239	1
1	UBE2R2	11	0	0.239	1
1	UBE2S	11	0	0.239	1
1	UBE2W	11	0	0.239	1
2	BRF2	2	0	0.214	1
2	CPSF7	6	0	0.215	1
2	ELL	3	0	0.214	1
2	GTF3A	3	0.193	0.346	0.333
2	PCF11	6	0	0.215	1
2	PHF5A	6	0	0.215	1
2	POLR2D	8	0.000483	0.216	0.678
2	POLR2I	8	0.000483	0.216	0.678
2	POLR2K	12	0.159	0.271	0.318
2	POLR3GL	3	0.00812	0.265	0.666
2	PTBP1	6	0	0.215	1
2	PTRF	1	0	0.213	0
2	TAF13	3	0	0.214	1
3	ADD2	1	0	0.282	0
3	ARID1A	2	0	0.351	1
3	ATOH8	1	0	0.282	0
3	BHLHE40	2	0.00949	0.302	0
3	FNIP1	1	0	0.282	0
3	HOXB13	1	0	0.282	0
3	IRAK1BP1	1	0	0.282	0
3	MC1R	1	0	0.282	0
3	NR4A2	2	0.0130	0.309	0
3	TCF7L2	14	0.378	0.391	0.0219
3	TLE3	2	0	0.282	1
3	TLE4	2	0	0.282	1
4	CCDC6	1	0	0.252	0

4	EML4	1	0	0.252	0
4	EREG	2	0	0.252	1
4	GRB14	1	0	0.252	0
4	HBEGF	2	0	0.252	1
4	NRAS	8	0.121	0.336	0.0357
4	PTPRA	1	0	0.252	0
4	SHOC2	1	0	0.252	0
5	CDH1	7	0.0875	0.287	0.0952
5	CRYAB	1	0	0.223	0
5	CTNNAL1	2	0	0.224	1
5	KLF4	4	0.0474	0.318	0.166
5	KLF6	4	0.0698	0.347	0.333
5	KLRG1	1	0	0.223	0
5	LIMA1	1	0	0.223	0
5	PCDH1	2	0	0.224	1
6	APC2	2	0	0.259	1
6	ARPP19	1	0	0.259	0
6	MIS12	3	0	0.260	1
6	PPP2CB	9	0.104	0.348	0.194
6	RANGAP1	3	0	0.260	1
6	SKA2	3	0	0.260	1
6	TBL1Y	2	0	0.259	1
7	DLL3	5	0.0115	0.255	0.7
7	JAG1	5	0.0115	0.255	0.7
7	MIB1	3	0	0.204	1
7	NEURL	3	0	0.204	1
7	NOTCH2	6	0.0293	0.255	0.466
7	NOTCH3	4	0.102	0.334	0.5
7	PLK3	1	0	0.204	0
8	EDN2	1	0	0.204	0
8	GNAQ	6	0.0876	0.256	0
8	NMS	1	0	0.204	0
8	OXTR	2	0.102	0.334	0
8	P2RY6	1	0	0.204	0
8	RGS2	1	0	0.204	0
8	SLC9A3R1	1	0	0.204	0
9	APEX1	6	0.933	1	0.0666
9	LIG1	1	0	0.545	0
9	MBD4	1	0	0.545	0
9	MUTYH	1	0	0.545	0

9	NTHL1	1	0	0.545	0
10	MPG	2	0	0.6	1
10	XRCC1	2	0	0.6	1

[1]Modules were determined by implementing Reactome FI application as shown in Fig.7.
