# Supplementary material for: Helveticoside is a biologically active component of the seed extract of Descurainia sophia and induces reciprocal gene regulation in A549 human lung cancer cells
Source: BMC Genomics. 2015 Sep 18;16(1):713. doi: 10.1186/s12864-015-1918-1 (PMC4575430; doi:10.1186/s12864-015-1918-1)
Supplement: Additional file 14: — Enrichment map analysis of the genes induced by helveticoside treatment in A549 cells. The network structure of the GO terms was constructed by implementing the Enrichment map plugin for Cytoscape. The simple GO enrichment results from 1,093 genes with the down-regulated pattern and 824 genes with the up-regulated pattern were used as input data. Each node represents a GO term sized proportionally to the number of genes encompassed by that GO term. The edge thickness signifies the closeness of the two nodes measured by their number of common genes. The representative biological functions for the groups of nodes are shown. (PDF 713 kb) [file 12864_2015_1918_MOESM14_ESM.pdf]

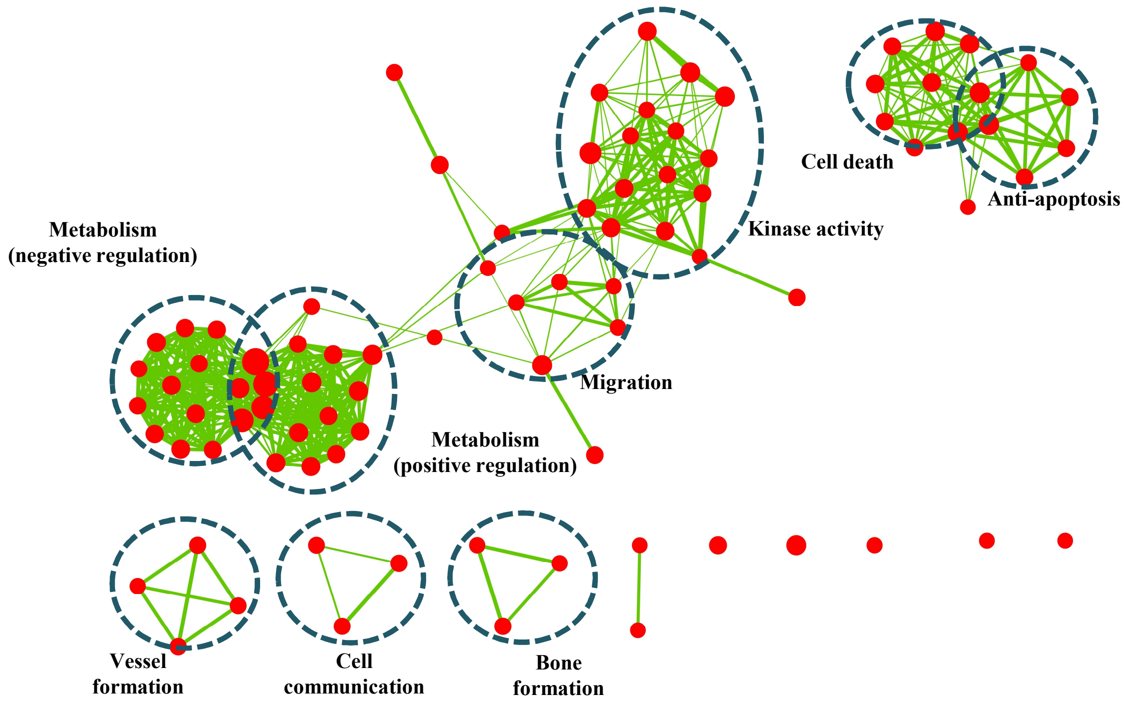

Additional file 14. Enrichment map analysis of the genes induced by helveticoside treatment in A549 cells. The network structure of the GO terms was constructed by implementing the Enrichment map plugin for Cytoscape. The simple GO enrichment results from 1,093 genes with the down-regulated pattern and 824 genes with the up-regulated pattern were used as input data. Each node represents a GO term sized proportionally to the number of genes encompassed by that GO term. The edge thickness signifies the closeness of the two nodes measured by their number of common genes. The representative biological functions for the groups of nodes are shown.
