# Supplementary material for: Helveticoside is a biologically active component of the seed extract of Descurainia sophia and induces reciprocal gene regulation in A549 human lung cancer cells
Source: BMC Genomics. 2015 Sep 18;16(1):713. doi: 10.1186/s12864-015-1918-1 (PMC4575430; doi:10.1186/s12864-015-1918-1)
Supplement: Additional file 15: — Association of GO terms with the cancer-related genes in the Enrichment map. The association of GO terms with the cancer-related genes is shown, and the blue diamond in the center represents the cancer-related genes obtained from the DiseaseHub database. A yellow node indicates a GO term with cancer-related genes, and the thickness of the purple edge is proportional to the number of genes. (PDF 6877 kb) [file 12864_2015_1918_MOESM15_ESM.pdf]

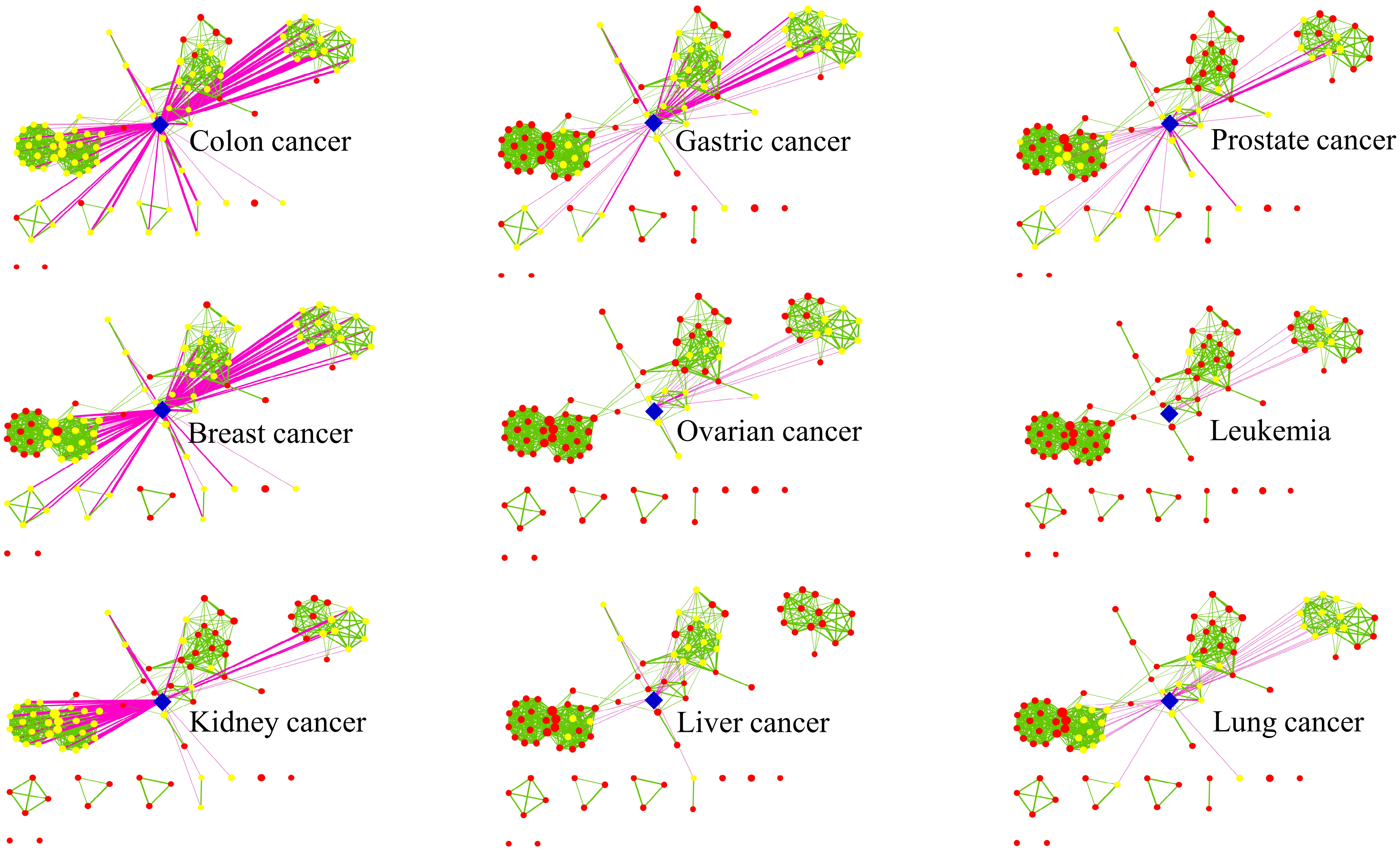

Additional file 15. Association of GO terms with the cancer-related genes in the Enrichment map. The association of GO terms with the cancer-related genes is shown, and the blue diamond in the center represents the cancer-related genes obtained from the DiseaseHub database. A yellow node indicates a GO term with cancer-related genes, and the thickness of the purple edge is proportional to the number of genes.
